# CRISPR Generated SIX6 and POU4F2 Reporters Allow Identification of Brain and Optic Transcriptional Differences in Human PSC-Derived Organoids

**DOI:** 10.3389/fcell.2021.764725

**Published:** 2021-11-16

**Authors:** Karl J. Wahlin, Jie Cheng, Shawna L. Jurlina, Melissa K. Jones, Nicholas R. Dash, Anna Ogata, Nawal Kibria, Sunayan Ray, Kiara C. Eldred, Catherine Kim, Jacob S. Heng, Jenny Phillips, Robert J. Johnston, David M. Gamm, Cynthia Berlinicke, Donald J. Zack

**Affiliations:** ^1^Shiley Eye Institute, University of California, San Diego, San Diego, CA, United States; ^2^Wilmer Eye Institute, Johns Hopkins University School of Medicine, Baltimore, MD, United States; ^3^Department of Cell Biology, Johns Hopkins University School of Medicine, Baltimore, MD, United States; ^4^Department of Neuroscience, Johns Hopkins University School of Medicine, Baltimore, MD, United States; ^5^Department of Molecular Biology and Genetics, Johns Hopkins University School of Medicine, Baltimore, MD, United States; ^6^Department of Ophthalmology and Visual Science, Yale School of Medicine, New Haven, CT, United States; ^7^Waisman Center, University of Wisconsin-Madison, Madison, WI, United States; ^8^Department of Ophthalmology and Visual Sciences, University of Wisconsin School of Medicine and Public Health, Madison, WI, United States; ^9^McPherson Eye Research Institute, University of Wisconsin-Madison, Madison, WI, United States; ^10^Department of Genetic Medicine, Johns Hopkins University School of Medicine, Baltimore, MD, United States

**Keywords:** optic, microenvironment, SIX6, Pou4f2, hypoxia, vesicle, organoid, retina

## Abstract

Human pluripotent stem cells (PSCs) represent a powerful tool to investigate human eye development and disease. When grown in 3D, they can self-assemble into laminar organized retinas; however, variation in the size, shape and composition of individual organoids exists. Neither the microenvironment nor the timing of critical growth factors driving retinogenesis are fully understood. To explore early retinal development, we developed a SIX6-GFP reporter that enabled the systematic optimization of conditions that promote optic vesicle formation. We demonstrated that early hypoxic growth conditions enhanced SIX6 expression and promoted eye formation. SIX6 expression was further enhanced by sequential inhibition of Wnt and activation of sonic hedgehog signaling. SIX6 + optic vesicles showed RNA expression profiles that were consistent with a retinal identity; however, ventral diencephalic markers were also present. To demonstrate that optic vesicles lead to bona fide “retina-like” structures we generated a SIX6-GFP/POU4F2-tdTomato dual reporter line that labeled the entire developing retina and retinal ganglion cells, respectively. Additional brain regions, including the hypothalamus and midbrain-hindbrain (MBHB) territories were identified by harvesting SIX6 + /POU4F2- and SIX6- organoids, respectively. Using RNAseq to study transcriptional profiles we demonstrated that SIX6-GFP and POU4F2-tdTomato reporters provided a reliable readout for developing human retina, hypothalamus, and midbrain/hindbrain organoids.

## Introduction

Retinal degenerative (RD) diseases range from genetically complex age-related macular degeneration (AMD), which is the most common cause of irreversible blindness in the elderly in the Western world, to Mendelian inherited orphan diseases such as retinitis pigmentosa (RP) and Leber Congenital Amaurosis (LCA). Although there have been important improvements in treatment approaches for the neovascular, or the “wet,” form of AMD, the more common atrophic, or “dry,” form of AMD and most forms of RP and LCA remain untreatable. Recent developments in stem cell biology and gene-editing offer improved models for the study of these diseases that could lead to novel cell-based treatment approaches. Particularly promising are 3D retinal organoids derived from human pluripotent stem cells (PSCs) which produce all five major neuronal cell types in the retina [i.e., photoreceptor (PRs), bipolar (BCs), horizontal (HCs), amacrine (ACs), and ganglion cells (RGCs)] (Meyer et al., 2009; Nakano et al., 2012; Kuwahara et al., 2015; Lowe et al., 2016; Volkner et al., 2016; Browne et al., 2017; Wahlin et al., 2017; Eldred et al., 2018; Capowski et al., 2019). In limited examples, they also respond to light (Zhong et al., 2014; Hallam et al., 2018).

The forced aggregate approach for generating retinal organoids is a gravity-based method whereby a defined number of PSCs seeded into a round bottom plate form solitary spheres that undergo neural induction and vesicle formation. Some vesicles become forebrain-like while others acquire a retinal identity. Much like the native retina, optic vesicles develop into organized laminar structures that emulate many of the temporal and spatial characteristics of *in vivo* development, including PR outer segment formation. Thus, the 3D retina system offers the possibility of being used for retinal disease modeling (Tucker et al., 2013; Deng et al., 2018; Huang et al., 2019; Lukovic et al., 2020). Both intrinsic and micro-environment factors affect organoid development (Zuber et al., 2003). Even technical details can impact organoid development. For example, a beneficial effect of low oxygen on cultured cells has been recognized for decades, yet most labs maintain cells under standard atmospheric [O_2_] conditions (Taylor et al., 1978; Bottenstein and Sato, 1985). Hypoxia participates in maintaining PSCs, neural progenitors and the developing nervous system (Abdollahi et al., 2011). Under hypoxia, PSCs divide faster, have less cleaved caspase-3, and exhibit fewer chromosomal abnormalities (Lim et al., 2011). In addition to stem cell maintenance, hypoxia can also promote early stages of retinal differentiation; mouse ES cells differentiated under hypoxia show elevated PR numbers (Bae et al., 2012; Garita-Hernandez et al., 2013; Chen T. et al., 2010; Chen et al., 2016). While the mechanisms involved are not well understood, several diffusible factors, including Sonic hedgehog (SHH) and VEGF are upregulated during hypoxia which might influence early neural patterning (Tsuzuki et al., 2000; Hwang et al., 2008). Despite the great potential of PSCs, differentiation protocols are not completely standardized (Meyer et al., 2011; Nakano et al., 2012; Zhong et al., 2014; Kuwahara et al., 2015; Singh et al., 2015; Zhou et al., 2015) and variability between organoids can exist.

To probe the role of the microenvironment on differentiation we used CRISPR-Cas9 genome-editing to develop a SIX6-GFP fluorescent reporter PSC line that allowed us to track eye field cells during early ocular development. SIX homeobox 6 (SIX6), also called OPTX2, is one of the earliest “optic genes” to be expressed *in vivo* and appears first in the anterior neural plate, and later in the ventral forebrain and optic vesicles (Toy and Sundin, 1999). Outside of the eye, it is only found in the hypothalamus and pituitary (Ozone et al., 2016). Within the eye, it is first expressed in early precursors where it activates retina-specific genes (Toy et al., 1998; Toy and Sundin, 1999). Using an unbiased high-content imaging approach, we exploited the SIX6-GFP reporter system to optimize microenvironment conditions that favored the generation of SIX6-GFP + structures. Among these, hypoxia, WNT signaling, and SHH signaling were all major contributors of early eye development. By optimizing these variables, the live-cell fluorescent readout of our system led to the generation of organoids in which most organoids formed SIX6-GFP + vesicles. We corroborated the identity of SIX6 + vesicles by introducing a second reporter, a reporter for retinal ganglion cells, POU4F2(BRN3B)-tdTomato, which is expressed only in bonafide retinas. By studying the transcriptional profiles of SIX6 + /POU4F2 +, SIX6 + / POU4F2-, and SIX6- structures we verified that SIX6-GFP labeled retina and hypothalamus, POU4F2-tdTomato labeled only retinas and SIX6- organoids were largely midbrain-hindbrain like. Thus, a SIX6 and POU4F2 dual color reporter is well suited for the sequential study and optimization of early stage human retinogenesis and identification of hypothalamic and MBHB brain structures.

## Materials and Methods

### Cells

Line IMR90.4 iPSCs were obtained from WiCell (Madison, Wisconsin). Cells were routinely tested for mycoplasma by PCR (Drexler and Uphoff, 2002). Pluripotency of cells was evaluated with antibodies for NANOG, OCT4, SOX2, and SSEA4. G-band karyotype analysis was carried out by Cell Line Genetics (Madison, WI, United States) (Supplementary Figure 1). PSCs were used with authorization from the JHU and UC San Diego Institutional Review Board Committee’s.

### PSC Maintenance

Stem cells were maintained antibiotic free on 1% (vol/vol) Matrigel-GFR (#354230; BD Biosciences) coated dishes at 37°C under hypoxic conditions (10% CO_2_/5%O_2_) in mTeSR1 (Stem Cell Technologies) (Ludwig et al., 2006; Yao et al., 2006; Chen et al., 2011; Wahlin et al., 2017). Cells were passaged every 4–6 days, with Accutase (#A6964; Sigma) for 8–10 min, dissociated to single cells, quenched with mTeSR1 plus 5 μM (-) blebbistatin (B; #B0560; Sigma), pelleted at 80 × *g* for 5 min, resuspended in mTeSR1 + B and plated at 5,000 cells per 35 mm dish (Walker et al., 2010). After 48 h, cells were fed without B.

### Cell Culture Medium

BE6.2-NIM (B27 + E6 at 2X concentration) (neural induction medium) consists of DMEM (#11965; Invitrogen) supplemented with 1% B27 vitamin A (-) (#12587010; Invitrogen) and 2X E6 supplement (38.8 mg/L insulin (#11376497001; Roche), + 128 mg/L L-ascorbic acid (#A8960; Sigma), 28 μg/L selenium (#S5261; Sigma), 21.4 mg/L transferrin (#T0665; Sigma) and 38.8 mg/L NaHCO3). Osmolarity was raised + 30 mOsm to ∼330–340 mOsm) by adding 0.88 g/L NaCl (Ludwig et al., 2006; Chen et al., 2011). LTR (Long-Term Retina) medium was a 3:1 mix of DMEM:F12 (#11965, #11765; Invitrogen) supplemented with 1% B27 (#17504044; Invitrogen), 10% heat inactivated qualified-grade FBS (#16140071; Invitrogen), 1 mM pyruvate (#11360; Invitrogen), 1xNEAA (#11140; Invitrogen), 1xGlutamax (#35050061; Invitrogen) and 1 mM taurine (#T-8691; Sigma). For fluorescent live cell imaging, we substituted DMEM (#11965; Invitrogen) with FluoroBrite DMEM (#A1896701; Invitrogen) + 2 mM glutamine.

### CRISPR-Cas9 Gene-Editing

A SIX6-GFP donor was synthesized by amplifying a 1,273-base pair (bp) homology arm fragment upstream of the SIX6 stop codon, a histone 2 binding domain (H2B) fused to an enhanced GFP cassette upstream, and a 1,222 bp fragment homology arm distal to the stop codon of the SIX6 gene; these were assembled by overlap extension PCR using Phusion polymerase and inserted into the ZeroBlunt TOPO vector (Invitrogen). For guide RNA construction, targeting was directed at the stop codon (**G**ACATCTGAGTTGCCCATCC**AGG**) and the guide RNA vector (Addgene #41824) synthesized as described by Mali et al. (2013) using the SIX6guide_F and SIX6guide_R primers (Supplementary Table 1). After transformation into chemically competent Stable *E. coli* (#C3040; NEB), colonies were miniprep, sequence verified, and plasmid DNA prepared for transfection using a Qiagen Endo-free Maxiprep kit. A POU4F2-p2A-tdTomato donor was generated by amplifying a 1,740 bp DNA donor fragment and a P2A-tdTomato that was inserted by Gibson assembly in-frame before the stop codon. A U6-guide-scaffold cassette was used for targeting the stop codon (**G**CCGGCATTTAGAAGACTCT**TGG**) of POU4F2. For transfection, PSCs were Accutase treated for 10 min, and 200,000 cells pelleted at 80 × *g*. For the SIX6-GFP single reporter line, electroporation was carried out in 10 μl’s of R-buffer containing 300 ng gRNA vector, 500 ng Cas9 vector (Addgene #41815) and 1 μg SIX6-GFP DONOR vector using a Neon transfection system (Invitrogen) with the following settings (1300 V, 20 ms, 1 pulse). After transfection, clonal iPSC colonies were lysed in Quick Extract buffer (Epicenter) and PCR verified using one flanking and one nested oligonucleotide primer (see supplement for list of oligos). Oligos flanking the homology arms were used to confirm homo- or heterozygosity and PCR products were sequence verified. For the SIX6-p2A-GFP/POU4F2-p2A-tdTomato dual reporter IMR90.4 iPSC line, the SIX6 and POU4F2 DNA donor plasmids were engineered with U6-gRNA cassettes on the same plasmid to facilitate transfection of each transgene. Single and dual reporters are from the same genetic background but underwent genetic modifications at different times. As before, integration was assessed by PCR and sanger sequencing.

### Optic Vesicle (OV) and Long-Term Differentiation of Retina Cups (RC)

See supplemental protocol for full details. Briefly, stem cells maintained under hypoxic conditions (5%O_2_/10%CO_2_), were passaged with Accutase for 12 min and 1,000–3,000 cells in 50 μl’s of mTeSR1 + B were seeded per well into a polystyrene 96-well U-bottom plate (#650180; Greiner) and placed back into hypoxia. This was designated as day 0 (D0). Over the first 4 days, aggregates were transitioned to neural induction medium (BE6.2-NIM) by adding 50 μl BE6.2 + 2% MG on day 1 and 50 μl BE6.2 + 1% MG each day thereafter. On D4–8 a 50% medium exchange (100 μl’s) was performed daily and every other day thereafter. NIM also contained 3 μM of the WNT antagonist (IWR-1-endo (IWR-1e); #681669; EMD Millipore) from D1–6. Although most experiments transitioned organoids to normoxia (20%O_2_/5%CO_2_) at day 5, organoids were also transitioned between D1–15 to test the importance of timing for this transition. For hypoxia experiments, feeding occurred in ambient air for approximately 5 min and returned to hypoxia for growth. Organoids were grown in BE6.2 + 300 nM Smoothened agonist (SAG; #566660; EMD Millipore) from D8–D14 and then LTR + SAG from D14–D18. For longer term experiments (e.g., RNAseq) we used sharpened tungsten needles to excise optic vesicles from D10–12 as previously described (Wahlin et al., 2017). For IHC and RNAseq of D65 organoids, excised vesicles were maintained in parallel and sorted for analysis after fluorescence detection. Organoids were maintained in suspension in LTR at low density (<24–36/10 cm untreated 10 cm polystyrene petri dishes) and fed every 2–3 days. Poorly defined vesicles were periodically removed. To increase survival and differentiation, 500 nM all-trans retinoic acid (ATRA; #R2625; Sigma) was added to LTR from D20 and 10 μM DAPT from D28–42.

### Live Cell Imaging, Measurements, and Statistics

A Cellomics ArrayScan High Content Screening (HCS) System (Thermo Fisher) was used to document and analyze fluorescence of forced aggregates in 96 well U-bottom plates (Greiner # 650180). To avoid edge effects, we eliminated the outer wells from experimental analysis. For analysis, GFP fluorescence arbitrary units (gfp a.u.) were used to reflect the mean total intensity per object. In later studies we used an ImageXpress Micro Confocal High-Content Imaging System (Molecular Devices) for image acquisition and the MetaXpress software package with a custom module to segment and mask GFP + areas, allowing us to measure the intensity per unit area. Samples were visually inspected in the rhodamine channel to rule out autofluorescence or bleed through. Quantitative measurements for brightness and intensity were imported into Prism GraphPad and plotted as histograms. For statistical analysis, significance was determined using one-way ANOVA with Tukey multiple comparisons test with an alpha cutoff of 0.05.

### Fixation and Immunohistochemistry (IHC)

Organoids were fixed in 4% paraformaldehyde (PFA) in 0.1 M phosphate buffer and 5% sucrose for 25 min. These were immersed sequentially in 6.75 and 12.5% sucrose in PBS for 1 h each, 25% sucrose-PBS overnight, 1 h in a 2:1 ratio of 25% sucrose-PBS and OCT Tissue-Tek (Ted Pella), and snap-frozen on dry ice/isopentane. Frozen sections (8 μm thick) were mounted onto Superfrost Plus slides (Thermo Fisher) and incubated overnight with 1:500 monoclonal anti-islet1 (39.4D5-c; DSHB) in PBS containing 2% normal horse serum (NHS) and 0.2% Triton X-100. Secondary antibodies were anti-mouse IgG’s (H + L) coupled to Alexafluor-647 (Invitrogen, 1:1,000). 10 μg/ml 4′, 6-diamidino-2-phenylindole (DAPI) was used to visualize cell nuclei and sections were processed without primary antibody as controls. Images were acquired with an ImageXpress Micro Confocal High-Content Imaging System, pseudocolored and merged in ImageJ. Adjustments in brightness and contrast were made using ImageJ (NIH^1^) and/or Affinity Designer.

### RNA Extraction and Real-Time RT-PCR (qPCR) Analysis

DNAse I treated RNAs from 10–12 pooled RCs per time point were harvested for RNA isolation in RLT buffer containing 2% beta-mercaptoethanol using the RNeasy Mini Kit (Qiagen #74104) and resuspended in nuclease free water. RNA concentration and OD260/280 ratio were determined using a Nanodrop 1000 (Thermo-Scientific). Reverse transcription of 1 μg of RNA was carried out using the High-Capacity cDNA kit (# 4368814; Applied Biosystems) for 10 min at 25°C, 2 h at 37°C, and 5 min at 85°C. A no RNA negative control reaction was run parallel. For qPCR, we used a 1:30 dilution of the synthesized cDNA. Oligonucleotide sequences are listed in Supplementary Table 1. qPCR was carried out using SsoAdvanced Polymerase (Biorad) and samples normalized using the geometric means of RPL27 and RPL30 as described (de Jonge et al., 2007; Thomas et al., 2014).

### RNA Sequencing (RNASeq)

RNA from day 0 pluripotent stem cells (*n* = 2) or from SIX6-GFP + (*n* = 2), or SIX6-GFP- (*n* = 2) organoids at day 35 (pools of 12 organoids each) was extracted using the RNeasy mini kit (Qiagen #74104) and RNA quality analyzed using RNA 6000 Nano kit on an Agilent 2100 Bioanalyzer. RNA with an integrity value (RIN) > 8 was used for library preparation with the NextEra library prep kit (Illumina). RNAseq was carried out on the Illumina HiSeq platform with 3.6 million reads per sample. Raw data was mapped to the Human Genome (GRCh37) using TopHat v2.0.12 (Trapnell et al., 2012, 2013). Differential gene expression based on the negative binomial generalized linear models was performed with DESeq2 (v1.24.0) in R (Bioconductor v3.5) (Love et al., 2014). Statistical tests were done with DESeq2. Hierarchical clustering heat maps and volcano plots were generated in R. Ingenuity pathway analysis (IPA; Qiagen) was used to analyze related gene networks and up/down regulated genes (Supplementary Tables 3–14). For D65 organoids grown under similar conditions to D35 organoids, total RNA was isolated using a Zymo RNA microprep kit per manufacturer’s instructions. RNA quantity and quality were analyzed with a NanoDrop 2000 (Thermo Scientific) and 4200 TapeStation (Agilent Technologies), respectively. RNAseq libraries were generated for D65 SIX6-, SIX6 + and SIX6 + /POU4F2 + organoids (*n* = 4 each) using rRNA depleted RNA samples and the TruSeq RNA Library Prep Kit v3 (Illumina). Sequencing was performed on a Novaseq 6000 (100 paired-end, Illumina) at the UCSD Institute for Genomic Medicine (IGM) Core to obtain 25 million reads per sample. Quality verification was performed using FASTQC (v0.72) and MultiQC (v1.8). Adaptor and quality trimming of reads were performed using Trimmomatic (v0.38). Reads were mapped to the human reference genome GRCh37.13 (hg19) using HISAT2 (v2.1.0). We also carried out differential sequence analysis using DESeq2 (v1.22.1) and EdgeR (v3.24.1), to obtain fold change (FC) differences between B3B + and SIX6-, B3B + and SIX6 + and SIX6 + and SIX6- samples (Robinson and Oshlack, 2010). Principal component analysis (PCA) was performed using DESeq2 while hierarchical clustered heatmaps were made using Pheatmap (v1.0.12). Volcano plots were made with ggplot2 (v3.3.3). For D65 volcano plots, genes significant genes had a FC > log1 and a false discovery rate (FDR) < 0.05. A Venn diagram was generated with jvenn (Bardou et al., 2014).

## Results

### SIX6 Reporter Cells Recapitulate Retinal Development

**FIGURE 1:**
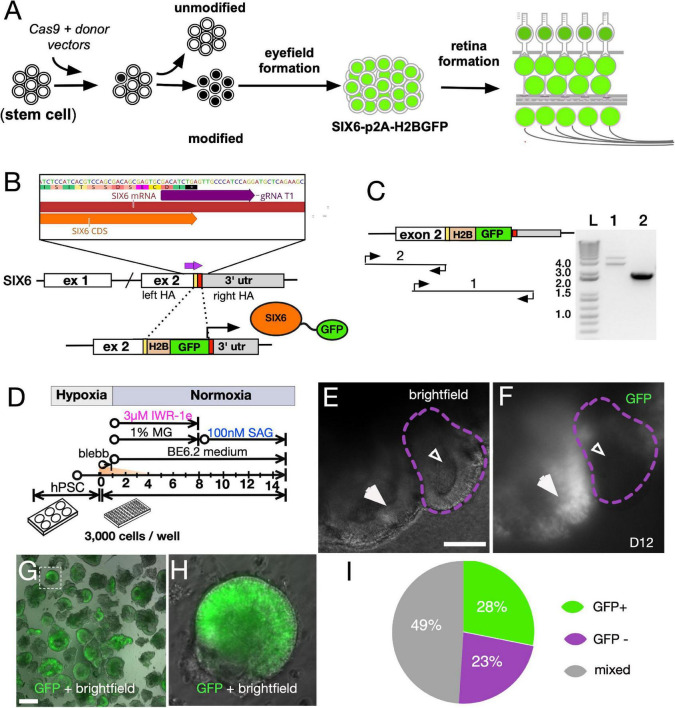
Directed differentiationdependence of SIX6 reporter PSCs into 3D retinas to validate reporter expression. **(A)** Schematic of stem cells modified with a SIX6-GFP reporter. **(B)** CRISPR/Cas9 targeting with a guide RNA spanning the stop site of the SIX6 gene. Donor fragment the H2B-GFP expression cassette with homology arms to facilitate homology directed repair (HDR). **(C)** Verification of the GFP cassette insertion by PCR amplification of genomic DNA with oligonucleotides that 1- flank the GFP insert (detects homo- and heterozygosity), and 2- flank the left arm and within the insert (detects insert). A 1Kb + DNA ladder (Invitrogen) was used for DNA band size resolution. **(D)** The experimental approach used for reporter validation. **(E)** Bright-field images and **(F)** reporter expression in some, but not all, vesicles at day 12. Large arrows **(E,F)** indicate GFP + vesicles while smaller arrows indicate GFP - vesicles. **(G)** Neural vesicles excised at D12 were live imaged for GFP fluorescence. μm **(H)** A magnified image from panel **(G)** (squared box) with GFP fluorescence overlaid onto a bright-field image. **(I)** Percentage of vesicles that exhibit GFP +, GFP – or mixed expression patterns following manual excision. Scale bars = **(E)** 175 μm, **(G)** 300 μm.

The vertebrate eye field first originates from the anterior neural plate and can be distinguished by the expression of *Six6*, *Pax6*, *Rx*, *Lhx2*, and *Vsx2* (Zuber et al., 2003). To monitor this early stage, we generated IMR90.4 induced pluripotent stem cell (iPSC) SIX6-H2B-GFP reporter cells using a CRISPR/Cas9 genome engineering approach (Figures 1A,B). A guide RNA (gRNA) targeting the stop codon of the *SIX6* gene was used with *S. pyogenes* Cas9 (SpCas9) to facilitate in-frame insertion of a nuclear localized histone 2B (H2B)-GFP just before the stop codon of *SIX6* (Figures 1B,C). Insertions were verified by PCR (Figure 1C) and Sanger sequencing. PCR was carried out with one oligonucleotide flanking the left homology arm and the other nested within the insert to detect insertion, while oligonucleotides flanking the insert were used to detect homo/heterozygosity. To rule out the possibility of mutagenesis at the “unedited” allele, we gel extracted and sequenced the lower band (Supplementary Figure 2). We next differentiated these cells toward a retinal lineage using a 3D retinal organoid approach as previously described (Wahlin et al., 2017; Figure 1D). After 10 days, SIX6-GFP + vesicles appeared as sporadic bulges near the surface and by 12 days, GFP was present along large areas of the neuro-epithelial surface (Figures 1E,F). Initially, auto-fluorescence from standard DMEM masked some of the SIX6-GFP signal so we substituted DMEM with FluoroBrite DMEM, which provided a greater signal to noise ratio. In addition to SIX6-GFP + vesicles (Figures 1E,F; large arrows), there were also GFP- vesicles (small arrows), confirming the co-existence of eye field and non-eye field neural structures. This is consistent with other reports demonstrating that optic vesicles can be identified by morphology (Meyer et al., 2009). To separate GFP + from GFP- neural structures, vesicles were mechanically excised using ultra-sharp tungsten needles, and visually inspected by morphology and GFP + signals (Figures 1G,H). At day 27, organoids showed significant variability in GFP expression despite a similar vesicle-like morphology (Supplementary Figure 3). Approximately 28% (27/96) of the excised vesicles were SIX6-GFP +, 23% (22/96) were negative for GFP, and the remaining 49% (47/96) had both GFP + and GFP- structures (Figure 1I), presumably due to the inherent variability during manual dissection. Manual selection of excised SIX6-GFP + vesicle made it possible to enrich for optic vesicle-like organoids for extended growth.

### Limiting Aggregate Size Leads to Elevated SIX6-GFP Expression

**FIGURE 2:**
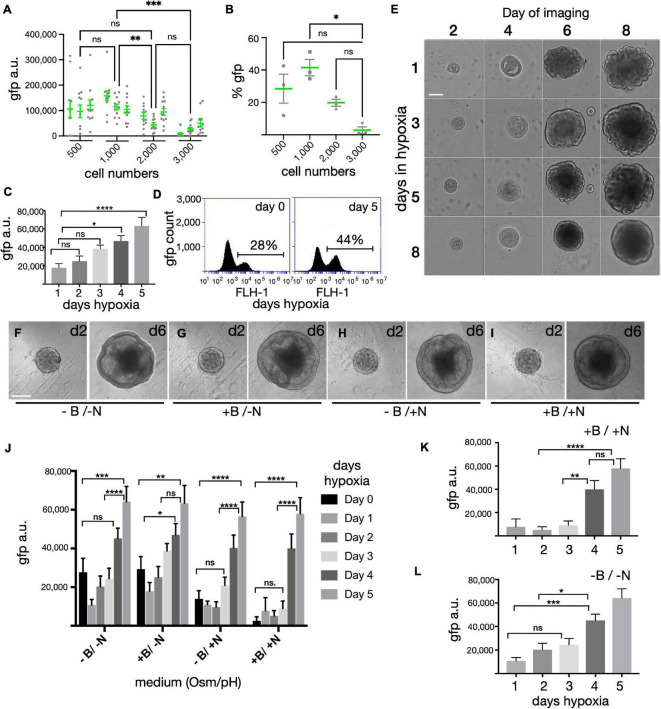
Optimization of organoid size, hypoxia and cell culture medium on SIX6-GFP expression. **(A)** Forced aggregates created by seeding 500–3,000 iPSCs per well in a U-bottom 96-well plate, were measured for GFP fluorescence at D15 by high-content imaging (*n* = 12/condition in triplicate; 144 total). **(B)** Organoids were pooled (*n* = 12/condition), dissociated and analyzed in triplicate by flow cytometry at D15 to correlate trends observed in panel **(A)**. **(C)** Fluorescence intensity of forced aggregates at D15 grown from 1,000 cells under hypoxia for 1, 2, 3, 4, or 5 days (*n* = 48 organoids; 240 total). **(D)** % of GFP cells by flow cytometry of organoids grown in hypoxia for 0 or 5 days (*n* = 48 pooled samples/condition). **(E)** Morphological assessment of organoids initiated under hypoxia for 1, 3, 5, or 8-days and imaged at 2, 4, 6, and 8-days. **(F–I)** Morphological assessment of organoids at 2- and 6-days post-aggregation after growth in high or low osmolarity medium (+ N vs. –N) with or without bicarbonate (+ B vs. –B). **(J)** Fluorescence intensity measurements of organoids after 15 days following growth in hypoxia for 0–5 days in media with different osmolarity and pH (*n* = 48 organoids; 1152 total). **(K,L)** The same values as in Panel **(J)**, but represented individually as N = sodium chloride (NaCl) and B = sodium bicarbonate (NaHCO3) (*n* = 48; 480 total). gfp(a.u.) = gfp arbitrary unit; Statistical significance determined by one-way ANOVA; * = statistically significant (*p* < 0.05), ** = statistically significant (*p* < 0.01), *** = statistically significant (*p* < 0.001), **** = statistically significant (*p* < 0.0001), n.s. = not significant. Scale bars **(E,F)** = 150 μm.

Aggregation of stem cells in U-bottom plates results in the formation of single spheres that are generally uniform in size. In previous work we titrated the seeding density from 3,000 to 9,000 cells per individual U-bottom well (Wahlin et al., 2017) and noted superior results with 3,000 cells - aggregates originating from 9,000 cells became overgrown with a necrotic core by day 10, while those from 3,000 cells yielded distinct neural vesicles, some of which produced retinas with outer segments. However, despite the retina-like appearance at later stages, it was often difficult to distinguish retinas at an early stage since both retina and non-retina tissues had a similar appearance. Our SIX6-GFP reporter allowed us to visualize early eye-field formation (Figures 1G–I) and test how initial seeding densities influenced optic development (Figures 2A,B). Spheroids initiated from 500–3,000 cells, were analyzed by high-content image analysis of SIX6-GFP + signals at day15 (Figure 2A). In terms of GFP signal intensity/unit area, 1,000 cells had a significantly higher GFP signal than those initiated from 3,000 cells (Figure 2A). 500 cells also yielded organoids with well-defined morphology; however, there was a wider range in GFP signals in individual organoids. To ensure that GFP intensity measurements by automated image analysis were reliable, we also dissociated and counted GFP + cells by flow cytometry from each condition in triplicate (Figure 2B). Like the imaging experiments, the % of GFP + cells was higher with 1,000 versus 3,000 cells. The option to use fewer cells for initiating organoids represents a significant technical advantage for high content applications requiring scalability for assay development and drug screening, though the need for manual cutting remains a bottleneck.

### Hypoxia Enhances SIX6-GFP Expression

We previously reported that PSCs maintained briefly under hypoxia yielded well-defined neural vesicles when organoids were initiated for 1 day under hypoxia; however, that determination was based on morphology (Wahlin et al., 2017). To expand on this with an eye field marker, we measured SIX6-GFP expression following varying lengths of hypoxia. Aggregates formed with 1,000 PSCs under extended hypoxia showed a time dependent response with elevated SIX6-GFP expression by day 10 and robust GFP levels by day 15 (Figures 2C,D). Image analysis of whole organoid live cell imaging revealed a greater than three-fold increase from 1 to 5 days of hypoxia (Figure 2C). Since this rise did not plateau during the initial 5 days, we expanded this study to include 8, 10, and 15 days (not shown). Cells grown under hypoxia for longer periods (8, 10, and 15 days) had a strong GFP signal but were smaller, rounded and became necrotic. Analysis of pooled samples by flow cytometry revealed that there were 28 and 44% SIX6-GFP + cells after 0 and 5 days of hypoxia, respectively (Figure 2D). Hypoxia also led to differences in organoid morphology (Figure 2E). After 1 day hypoxia (Figure 2E - top row), spheres exhibited more vesicles with many folded structures at day 8 compared to samples under hypoxia for the entire 8 days (Figure 2E - bottom row), which had smoother contours, fewer vesicles, and a smaller diameter. While cells under hypoxia for 3 or 5 days both showed intermediate levels of vesicle formation, 5 days was optimal for GFP expression (Figure 2C) and for generating visible neural vesicles (Figure 2E) amenable to manual dissection.

### Systematic Optimization of Media Conditions for Early Eye Field Growth

In previous work, retina structures were identified at early stages by morphology and at later stages when easily discernible outer segments began to form (>160 days). The absence of a fluorescent readout at early stages made it difficult to optimize the differentiation process; thus, early stage differentiation conditions were likely to be sub-optimal for retinal growth. The SIX6 fluorescent reporter allowed us to monitor the effects of growth media on differentiation. Using GFP as a readout, we optimized osmolarity, pH and hypoxia (Figures 2F–L). Salt concentrations in different basal media vary with DMEM, ACSF and BrainPhys each containing 120 mM sodium chloride (NaCl) (∼290 mOsm), and Neurobasal containing 70 mM NaCl (220 to 250 mOsm) (Bardy et al., 2015). While stem cells thrive in higher osmolarity (330–340 mOsm) (Ludwig et al., 2006), neurons grow better in lower osmolarity (Brewer et al., 1993; Bardy et al., 2015). One day after aggregation in mTeSR1, organoids were supplemented with an equal volume of BE6.2 neural induction medium (BE6.2 NIM) comprised of DMEM, B27 (w/o Vitamin A), and Essential 8 (E8) supplement (minus FGF2 and TGF-beta) at twice the normal concentration. To mimic the higher osmolarity of stem cell medium and prevent osmotic stress during the transition to BE6.2 NIM, we raised the osmolarity by 30 to 330 mOsm. After 2 and 6 days of development we compared the morphology of spheroids maintained in lower (-N) and higher (+ N) osmolarity growth medium and observed no clear difference in morphology (Figures 2F–I, respectively). However, when evaluating SIX6-GFP at day 15, we noted significantly more GFP in lower osmolarity medium, particularly after 1–3 days hypoxia (Figure 2J). Since we previously grew PSCs under hypoxia (10%CO2/5%O2) and later differentiated them under normoxia (5%CO2/20%O2), another potential source of variation was the pH of the medium (Wahlin et al., 2017). To assess the impact of pH, we supplemented the media with 1.0 g/L of NaHCO3. While there were no differences in morphology between low (-B) and high pH (+ B) at early stages (Figures 2F–I), we did observe significant differences in GFP signals at day 15 (Figures 2J–L). Higher NaHCO_3_ resulted in elevated SIX6-GFP under lower osmolarity (+ B/-N) and reduced GFP under higher osmolarity (+ B/ + N). While modifying the pH was helpful, the greatest effect came from extended hypoxia. Overall, we found the optimal conditions for promoting SIX6 expression to be those with lower osmolarity, higher pH and extended hypoxia.

### Early Sustained WNT Inhibition Enhances SIX6 Expression

**FIGURE 3:**
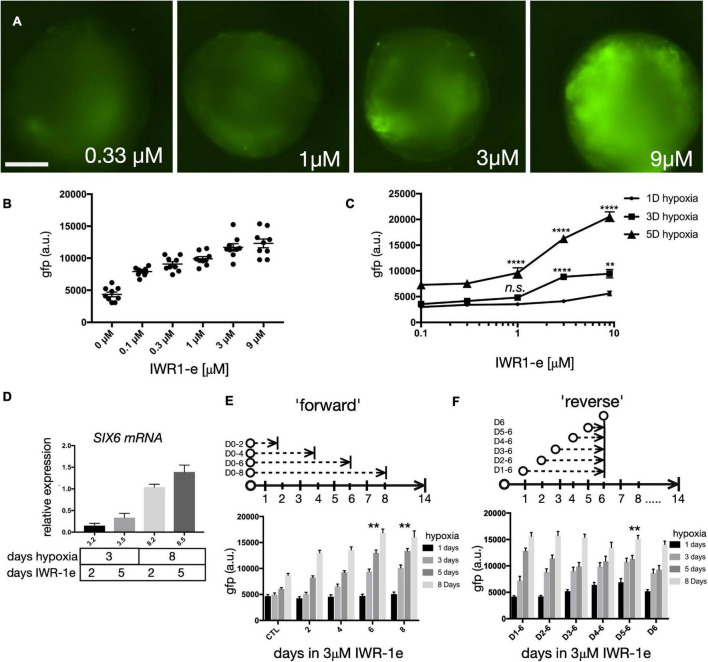
Dose and time of WNT signaling on eye field development. **(A)** SIX6-GFP fluorescence imaging of live organoids at day 14 that were grown under hypoxia for 5 days and treated with 0.33, 1, 3, and 9 μM of IWR1e from days 1–6. **(B)** Average intensity fluorescence of SIX6-GFP organoids following a dose response of IWR1e (0.11, 0.33, 1.0, 3.0, and 9.0 μM; *n* = 9 individual organoids; 54 total). **(C)** The influence of a dose response of IWR1-e on fluorescence intensity for cells grown under different hypoxic conditions (1-, 3-, and 5 days) and imaged at D14 (*n* = 10; 180 total). **(D)** Quantitative PCR (qPCR) showing relative expression of SIX6 in organoids grown under hypoxia for 3 and 8-days of hypoxia and treated with 3.0 μM IWR1e for 2 or 5 days (*n* = 3; 12 total). **(E)** GFP intensity measurements at day 14 following a forward time course in which IWR1e was added for 0, 2, 4, 6, or 8 days under varying days of hypoxia (*n* = 10 individual organoids; 200 total). **(F)** GFP intensity measurements at day 14 following a reverse treatment scenario, in which IWR-1e was added during days 1–6, 2–6, 3–6, 4–6, 5–6, and only on D6 after variable lengths of hypoxia (*n* = 10; 240 total). gfp a.u. = GFP arbitrary unit; Statistical significance determined by one-way ANOVA; ** = statistically significant (*p* < 0.01), **** = statistically significant (*p* < 0.0001), n.s = not significant. Scale bar = 300 μm.

WNT inhibition promotes anterior neural development (Glinka et al., 1998; Brafman and Willert, 2017) and IWR-1-endo (IWR-1e) is a small molecule that inhibits WNT by stabilizing the β-catenin destruction complex component AXIN2 (Chen et al., 2009). IWR-1e has been used in PSC generated retina differentiation protocols; however, the optimal dose and timing has not been optimized (Nakano et al., 2012; Wahlin et al., 2017). To address this, we carried out a dose-response with IWR-1e beginning at day 1 using 0.11, 0.33, 1.0, 3.0, and 9.0 μM IWR-1e for 6 days with cells maintained for 5 days under hypoxia (Figures 3A,B). Compared with 0.11 μM IWR-1e that produced only weak SIX6-GFP expression at day 14, 9 μM IWR-1e produced a much stronger response. GFP images were acquired using an ImageXpress high-content microscope and quantified by measuring the GFP intensity of retinal organoids per sample and across replicates. To rule out non-specific fluorescence, organoids were also imaged in the Texas Red channel with minimal fluorescence bleed through observed. Since previous experiments showed a correlation between hypoxia and SIX6-GFP expression, we also tested a dose response of IWR-1e after 1, 3, and 5 days of hypoxia (Figure 3C). The combined effects of WNT inhibition and hypoxia were most pronounced when IWR-1e was combined with 5 days of hypoxia and 9 μM IWR-1e which showed a 3.66-fold increase in GFP expression compared with 1 day hypoxia. To verify the role of hypoxia at the RNA level, we grew aggregates for 3 or 8 days in hypoxia with 3.0 μM IWR-1e for either 2 or 5 days and performed reverse transcription quantitative PCR (RT-qPCR). Longer hypoxic treatment and IWR-1e resulted in higher SIX6 transcript levels relative to the RPL27 and RPL30 housekeeping genes (Figure 3D). We next defined the optimal window for WNT inhibition by treating organoids with 3 μM IWR-1e for varying intervals in “forward” and “reverse” dosing schemes designed to identify when a treatment should begin and end, respectively. The “forward” time course included treatments starting at day 0 for 2, 4, 6, or 8 days under 1, 3, 5, or 8-days hypoxia (Figure 3E). Relative to non-treated controls, longer IWR-1e treatments induced greater SIX6-GFP expression. In addition, organoids initiated in hypoxia for 1 day showed lower GFP levels compared to 3, 6 and, 8-day timepoints that showed progressively more GFP. Having established that blocking WNT for extended time enhances SIX6-GFP expression in a dose-dependent fashion, we next optimized when WNT inhibition should begin. A “reverse” time course consisted of 3 μM IWR-1e treatments from days 1–6, 2–6, 3–6, 4–6, 5–6 or a single dose on day 6 under 1, 3, 5, or 8-days hypoxia (Figure 3F). While subtle differences were observed with respect to IWR-1e time windows, the most notable changes occurred from hypoxia. A short hypoxic exposure led to low GFP + signals at D14 across all IWR-1e treatment windows, and longer hypoxic exposure (3, 5, and 8 days) led to progressively higher SIX6-GFP levels, particularly from D5–6. Thus, SIX6-GFP expression is enhanced by the combined effects of WNT inhibition and hypoxia.

### Sonic Hedgehog Signaling Enhances SIX6 Expression

**FIGURE 4:**
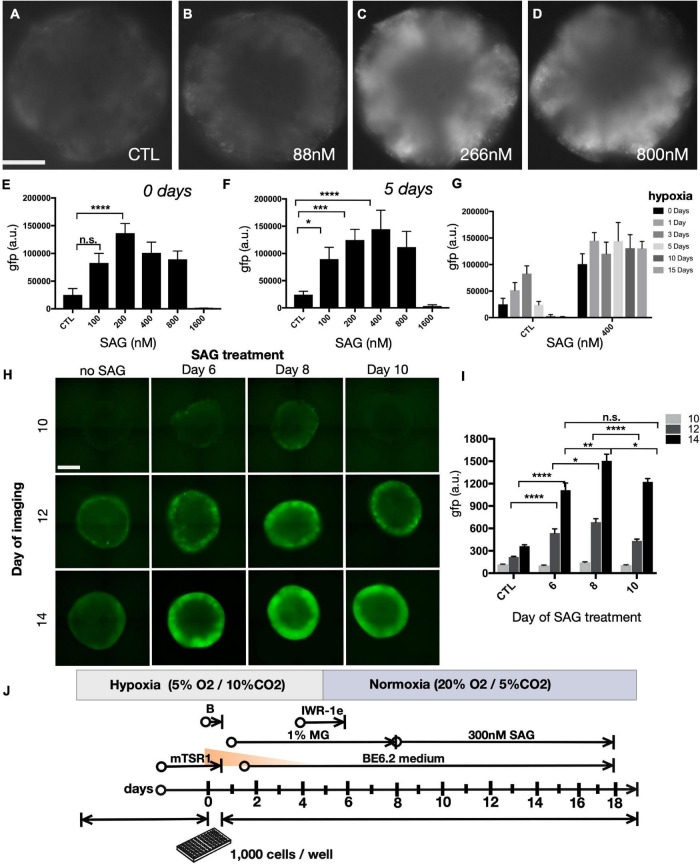
The effect of Shh agonist on SIX6-GFP expression during prolonged hypoxia. **(A–D)** Widefield images of SIX6-GFP fluorescence at day 15 in live organoids following treatment with smoothened agonist (SAG) up to 800 nM. **(E–G)** Fluorescence intensity measurements following treatment with an extended range of SAG up to 1600 nM. **(E,F)** The right side of the parabolic curve at the upper range of treatment reflects the inhibitory action of SAG at high concentrations (*n* = 12; 72 total). **(G)** SIX6-GFP fluorescence measurements at day 15 in control (untreated) and 400 nM SAG treated organoids exposed to varying lengths of hypoxia (0, 1, 3, 5, 10, and 15 days; *n* = 12; 72 total). **(H)** Fluorescence microscopy of live organoids at days 10, 12, and 14 following SAG treatments initiated at 6, 8, or 10 days and **(I)** their corresponding fluorescence intensity measurements (*n* = 15; 180 total). **(J)** Summary of optimized conditions for optic vesicle formation. Statistical significance determined by one-way ANOVA; gfp a.u. = GFP arbitrary unit; * = statistically significant (*p* < 0.05), ** = statistically significant (*p* < 0.01), *** = statistically significant (*p* < 0.001), **** = statistically significant (*p* < 0.0001), n.s. = not significant. Scale bars = 300 μm.

We also sought to optimize the effect of smoothened agonist (SAG), a potent sonic hedgehog (SHH) agonist that enhances retinal progenitor expansion (Frank-Kamenetsky et al., 2002). We evaluated SIX6-GFP following SAG treatment beginning at day 8 by imaging GFP expression at day 15. We observed maximal GFP levels at 266 nM (Figures 4A–D). We next expanded the SAG dose range from 100 nM to 1.6 μM in either normoxia (Figure 4E) or 5 days hypoxia (Figure 4F). Under normoxia, GFP levels were highest following a 200 nM SAG dose, while for 5 days hypoxia the optimal dose was 400 nM. Beyond 800 nM, SAG was inhibitory in both 0 and 5-day hypoxic conditions (Figures 4E–G). The completely diminished GFP signals at 1.6 μM in both conditions is consistent with inhibitory effects in the micromolar range previously reported (Chen et al., 2002). Thus, a 200–400 nM range of SAG was suitable across a range of hypoxic conditions for SIX6-GFP expression (Figure 4G). We next sought to identify the optimal timing for hedgehog agonists by initiating organoids in hypoxia for 5 days followed by treatment with 300 nM SAG starting at days 6, 8, or 10 (Figures 4H,I). Compared with DMSO controls that showed little GFP expression, treatments beginning at days 6 and 8 showed patchy expression at day 10 and robust expression by day 14 (Figures 4H,I). By contrast, treatment at day 10 rose by day 14 but not to the same extent as earlier treatments. Considering that other published protocols (Nakano et al., 2012; Wahlin et al., 2017) treated organoids with a lower dose of SAG beginning later at day 10, our data suggests that treating with a higher dose of SAG at an earlier point might be beneficial for enhancing SIX6 expression. Based on these results, an optimized set of conditions was determined to be 300 nM SAG beginning at day 8 under hypoxia for the first 5 days (Figure 4J).

### Differential Gene Expression in Early Stage SIX6-GFP + and GFP- Organoids

**FIGURE 5:**
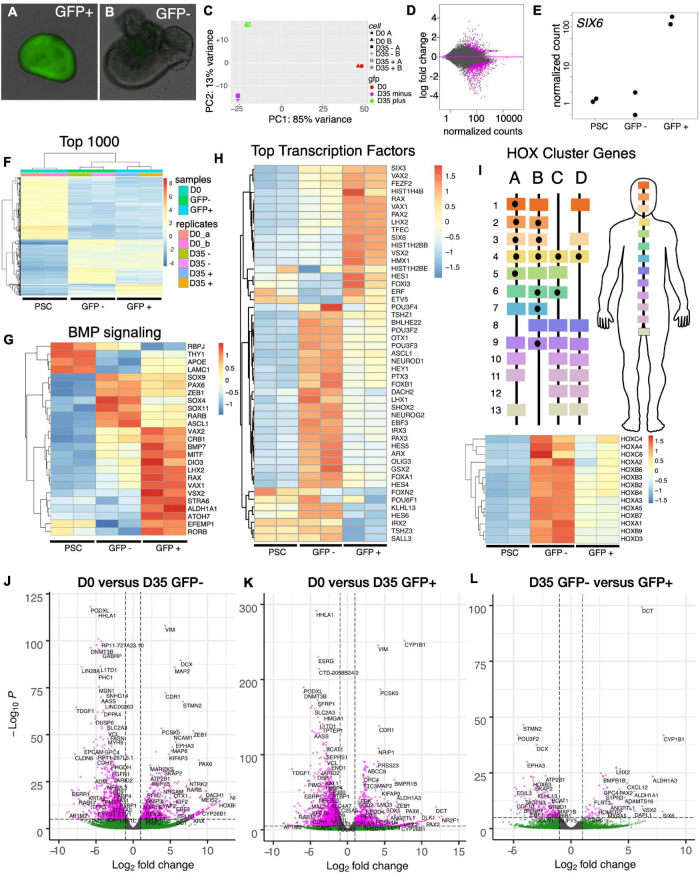
Transcriptional profiling of SIX6-GFP positive and negative neural vesicles. Analysis of undifferentiated IMR90.4 PSCs (day 0) and pooled SIX6-GFP + and - organoids (day 35) differentiated under conditions that promote anterior neural development. **(A,B)** SIX6-GFP + or – organoids imaged at D27 were pooled (*n* = 12) and RNA extracted at D35. **(C)** Principal component analysis (PCA) of undifferentiated PSCs (red), D35 SIX6-GFP positive (green), and D35 SIX6-GFP negative (magenta) organoids showing tight clustering between replicates. **(D)** MA-plot highlighting genes with altered expression (magenta). **(E)** Normalized counts of SIX6 expression in PSCs and SIX6-GFP + or - organoids. **(F)** A heat-map of the top 1,000 genes in PSCs and D35 organoids showing clustering of genes between replicates and differences between PSCs and vesicles (GFP + and –). **(G)** Ingenuity Pathway analysis (IPA) of genes linked to BMP signaling and hierarchical clustering of the top 25 genes in this category. **(H)** The top 50 transcription factor genes that are differentially expressed in PSCs, optic vesicles, and presumptive forebrain. **(I)** Homeobox (HOX) cluster gene enrichment in D35 SIX6-GFP negative organoids relative to PSCs and SIX6-GFP + vesicles. **(J–L)** Volcano plots showing transcriptional differences between **(J)** PSCs and D35 SIX6-GFP- organoids, **(K)** PSCs and D35 SIX6-GFP + organoids and **(L)** D35 SIX6-GFP- and + organoids with genes plotted that pass the thresholds of statistical significance (pCutoff ≤ 10e-6, and FCcutoff ≥ 1.0 log_2_).

To identify the cellular composition of SIX6-GFP + organoids, we explored the transcriptional differences between undifferentiated stem cells (PSCs), differentiated SIX6-GFP + organoids and SIX6-GFP- organoids. At day 35, organoids with cup-like morphologies (Figures 5A,B) were selected based on GFP fluorescence and mRNA transcripts were profiled by RNAseq. Principal component analysis (PCA) showed that replicate PSCs, or pools of GFP + and GFP- organoids clustered as discrete groups (Figure 5C). An MA-plot created by transforming the data onto M (log2 fold-change) and A (mean average) scales showed that expression of many genes (magenta) was significantly different between GFP + and - organoids (Figure 5D). To verify that the SIX6-GFP reporter correctly indicated *SIX6* expression, we compared normalized expression of *SIX6* transcripts in GFP + and - organoids, with undifferentiated PSCs (D0) as controls (Figure 5E). *SIX6* was highly expressed in GFP + samples, showing > 100-fold increase compared to undifferentiated PSCs or SIX6-GFP - organoids. To assess global differences in gene expression, hierarchical clustered heatmaps were then used to visualize the top 1,000 genes that were enriched in SIX6 + organoids versus SIX6- and PSC controls (Figure 5F). Replicates clustered together, with large differences evident between D0 PSCs and D35 GFP + and - organoids. Although less pronounced, there were also clusters that were different between D35 GFP + and - samples. The stem cell enriched genes *FGF2*, *POU5F1* (*OCT4*), *NANOG*, *LIN28A*, *PODXL*, and *MYC*, were highly enriched in PSCs but absent, or low in abundance, in organoids (Supplementary Figure 4). GFP + organoid enriched genes were *SIX6*, *CYP1B*, *VSX2*, *HMX1*, *RAX*, *LHX2*, *PAX2*, and *VAX1* (Supplementary Figure 5). Qiagen’s Ingenuity Pathway Analysis (IPA) was used to explore genes linked to various functional groupings. BMP signaling, which plays an important role in eye development, was linked with the genes *ALDH1A1, CRB1, DIO3, LHX2, MITF, RAX, STRA6, TFEC*, and *VSX2*, which were all elevated in SIX6 + organoids relative to SIX6 - organoids (Figure 5G). *ASCL1, RARB, PAX6, SOX4, SOX9*, and *SOX11* were highest in non-retinal organoids, while PSCs showed enrichment of *RBPJ*, *THY1*, *APOE*, *LAMC1*. There was also greater diversity of differentially expressed transcription factors in brain-like organoids compared with retinal organoids (Figures 5H,I). Notably, GFP- organoids were enriched for *ARX*, *GSX2*, *LHX1*, *OLIG3*, *OTX1*, and the *HOXA-D* family of genes (Figures 5H,I and Supplementary Figure 6). *HOX* genes are conveniently positioned along the anterior-posterior body axis with low numbered genes (e.g., *HOXA1, B1, etc.*) expressed at the anterior aspect of the hindbrain and high numbered genes (e.g., *HOXA13, C13*, etc.) expressed posterior in the spinal cord (Figure 5I). Statistically significant differences (pCutoff ≤ 10e-6, and log2FCcutoff ≥ 1.0) between undifferentiated PSCs and D35 GFP- organoids (Figure 5J; 33,565 datapoints), PSCs and D35 GFP + organoids (Figure 5K; 35,923 datapoints), and D35 SIX6-GFP + and – organoids (Figure 5L; 37,122 datapoints) were displayed as volcano plots. The stem cell markers *PODXL*, *LIN28*, *HHLA1*, *JARID2*, and *DPPA4* showed enrichment in stem cells (Figure 5J) while other genes such as *PAX6*, *CYP26B1*, *NR2F1*, and vimentin were enriched in both SIX6-GFP + and - organoids (Figures 5J,K) reflecting a general role in neural development. Other forebrain expressed genes (e.g., *ARX*, *MEIS2*, *POU3F2* [*BRN2*], and *HOXB5)* were differentially expressed in SIX6-GFP- organoids (Figures 5J,L) while early retinal genes (e.g., *ALDH1A3, CYP1B1, LHX2, SIX6, and VSX2*) were enriched in SIX6-GFP + organoids (Figure 5K). These data suggest that SIX6-GFP is useful for identifying optic vesicle organoids.

Next, we used pathway analysis to identify up/down regulated pathways in SIX6-GFP + and - organoids. These were sorted into the categories: cellular development, embryonic development, eye-related, nervous system development and function, and tissue and organ development (Supplementary Tables 3–14). The upregulated functional annotations with the highest *p*-value scores included sensory system development, formation of eye, development of sensory organs, and morphology of eye. Not surprisingly, many genes identified in the “formation of eye” category correlated with *ALDH1A1*, *CRB1, MITF*, *RAX*, *SIX3*, *STRA6, THY1, VAX1*, *VAX2*, and *VSX2*, which were all significantly upregulated in SIX6-GFP + vesicles. Genes categorized with the terms “formation of eye” (Supplementary Table 8) and “formation of brain” (Supplementary Table 11) correlated with SIX6-GFP + and - organoids, respectively. Genes that were downregulated relative to SIX6-GFP + organoids were associated with brain development, cerebral cortex and forebrain suggesting that the SIX6-GFP- structures were more brain-like than retina-like (e.g., *ARX*, *CBLN1*, *HOXA-D*, and *POU3F2*). Several genes from SIX6 + samples were also linked to a variety of developmental eye disorders (Supplementary Tables 4, 10). COL2A1, *WDPCP* and *HMX1* are linked with syndromic and systemic retinopathy and autosomal dominant or recessive retinal degeneration; *CRB1* is linked to early onset Leber congenital amaurosis (LCA8), and a severe form of autosomal recessive retinitis pigmentosa (RP12); and *EFEMP1* mutations affect RPE cells and are associated with both Malattia Leventinese and Doyne honeycomb retinal dystrophy. Mutations in other genes, including *VSX2* and *MITF*, can lead to abnormal development including defects in eye size and pigmentation (Moore, 1995; Reis et al., 2011). Mutations in the retinol binding protein *STRA6* can also cause isolated malformations and congenital anomalies of the eye (Casey et al., 2011).

### Identification of Bona Fide Retinas Using a SIX6 and POU4F2 Dual Reporter

**FIGURE 6:**
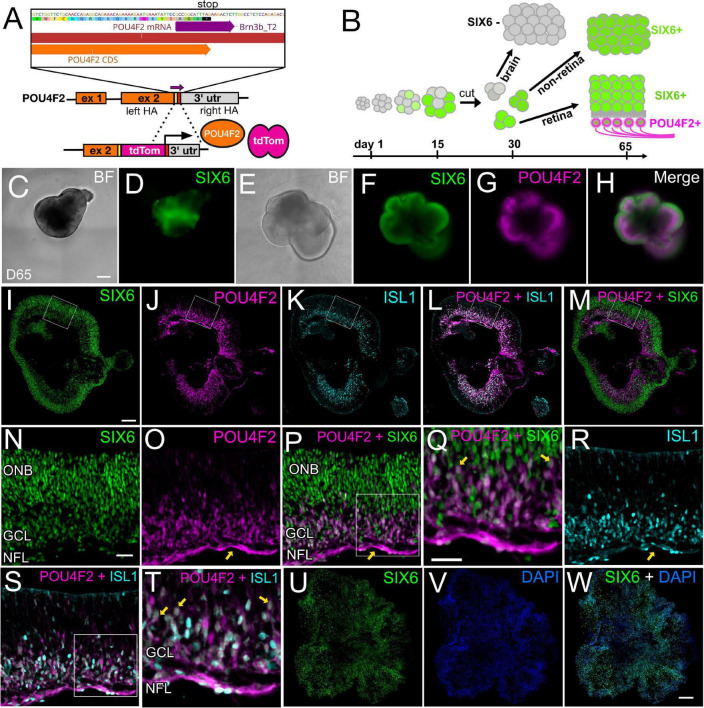
A SIX6-GFP and POU4F2-tdTomato dual color reporter for live-cell imaging of bona fide retinal organoids. **(A)** Diagram showing the targeting of a p2A-tdTomato cassette into the endogenous *POU4F2* locus. **(B)** Workflow differentiating different tissues and identification of retina or brain region based on fluorescence. **(C,D)** Live cell bright field and fluorescence imaging of a day 65 organoids expressing SIX6-GFP, but lacking POU4F2-tdTomato. **(E)** Live cell bright field image of retinas at the same age expressing **(F)** SIX6GFP, **(G)** POU4F2, and **(H)** a SIX6/POU4F2 merged image. Frozen cryosections of POU4F2 + organoids with images of **(I)** SIX6-GFP, **(J)** POU4F2-tdTomato, **(K)** ISL1, **(L)** POU4F2/ISL1, and **(M)** POU4F2/SIX6. High magnification images of **(N)** SIX6 label the entire retina with the strongest signals in the outer neuroblast (ONB) layer. **(O)** POU4F2 is localized to the inner aspect of the retina near the nerve fiber layer (NFL) (arrow), **(P)** POU4F2 and SIX6 merged, **(Q)** POU4F2 and SIX6 merged at high magnification, **(R)** immunostained with ISL1, **(S)** POU4F2 and ISL1 merged, **(T)** POU4F2 and ISL1 merged at high magnification, **(U)** SIX6 in non-retinal organoids, **(V)** DAPI counterstained, **(W)** SIX6 and DAPI merged. Scale bars in panels **(C–H)** = 300 μm; panels **(I–M,U–W)** = 200 μm; panels **(N–P,R,S)** = 33 μm, panels **(Q,T)** = 33 μm.

At day 35, SIX6 + organoids had gene expression profiles that included many classic retina markers (Figures 5G,H,K,L); however, SIX6 is also expressed in the hypothalamus and pituitary. To determine if the observed *SIX6* expression was present in retina, hypothalamus, and/or pituitary-like organoid structures, we generated a SIX6-GFP/POU4F2-tdTomato dual reporter iPSC line capable of distinguishing between bona fide retinas and other SIX6 + structures (Figures 6A,B). SIX6-GFP + signals emerged between days 12–14, while SIX6 + /POU4F2 + cells emerged after 35 days (not shown). All organoids appeared to be in an active state of proliferation as indicated by Click-It EdU (5-ethynyl-2′-deoxyuridine) incorporation which showed that most cells had incorporated EdU after an 18-h pulse (Supplementary Figure 7). At day 65, live-cell imaging of neural vesicles revealed three distinct populations: GFP + /tdTomato +, GFP + / tdTomato-, and GFP-/tdTomato- organoids. SIX6-GFP + organoids that did not express POU4F2 (Figures 6C,D) typically had smooth borders but were slightly darker in appearance by brightfield microscopy. Their GFP + signals were less organized and streaky in appearance suggesting a different tissue type. Importantly, these lacked POU4F2-tdTomato expression suggesting that they were not retinas. On the other hand, SIX6 + /POU4F2 + organoids (Figures 6E–H) had a classic optic vesicle-like appearance that was cup-shaped with translucent borders (Figure 6E) and had a more continuous GFP distribution throughout (Figures 6F,I). High magnification of tissue sections revealed that SIX6/POU4F2 dual + organoids expressed GFP in all retinal cells with the highest levels in the presumptive outer neuroblastic layer (ONB) (Figures 6N,P). Soma of SIX6 + /POU4F2 + cells were readily apparent in the prospective inner retina RGC layer (Figures 6G,H,J,L,M,O–Q,S,T) while axons were present in a rudimentary nerve fiber layer (Figures 6O,P; arrows) suggesting that these were retina-like. POU4F2-tdTomato + signals also co-localized by immunohistochemistry with ISLET-1 (ISL1; Figures 6K,L,R–T), a known RGC marker at this stage. In contrast, SIX6 + organoids without POU4F2 expression had no discernible retinal lamination (Figures 6U–W). Together, this data suggested that SIX6 labeled both retina and non-retina populations.

### Global Comparison of Organoids by RNA Sequencing

**FIGURE 7:**
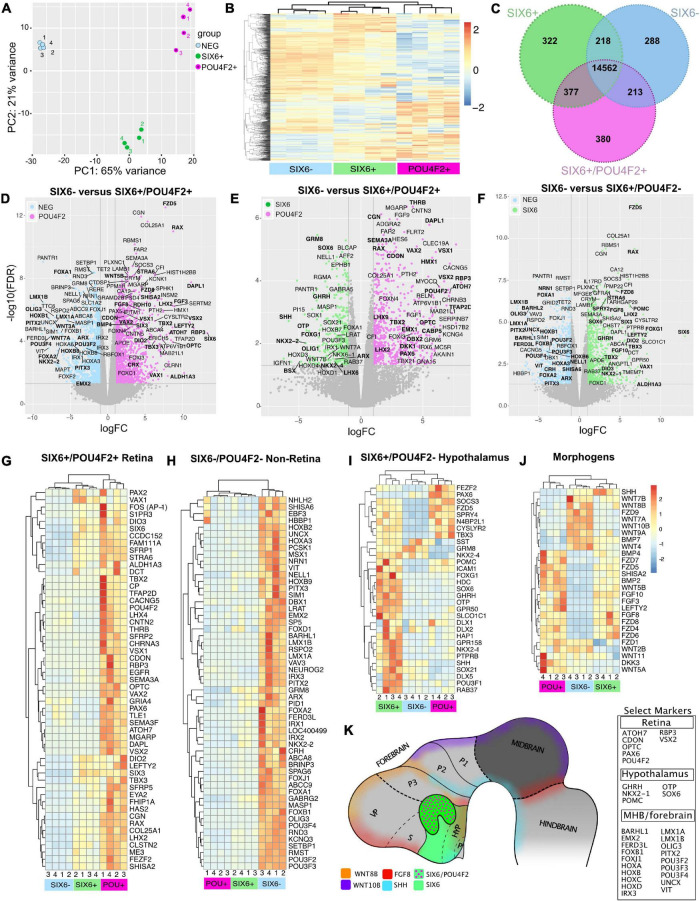
Gene expression signatures in SIX6-, SIX6 +, and SIX6 + /POU4F2 + organoids after 65 days. **(A)** A PCA plot showing clustering within organoid types. **(B)** A heatmap of the top 1,000 expressed genes in SIX6 + /POU4F2 + organoids compared with SIX6 + /POU4F2- and SIX6- organoids. **(C)** A Venn diagram showing the intersection of significant genes expressed by organoid types. **(D–F)** Volcano plots of significantly different genes between **(D)** SIX6- and SIX6 + /POU4F2 + organoids, **(E)** SIX6 + /POU4F2- and SIX6 + /POU4F2 + organoids, **(F)** and SIX6- and SIX6 + /POU4F2- organoids. Differences plotted as logFC vs. -log10(FDR) with significance set as FDR < 0.5 and a FClog2 > 1. **(G–J)** Hierarchical clustered heat maps show genes enriched in **(G)** SIX6 + /POU4F2 + retina, **(H)** SIX6-/POU4F2- non-retina, **(I)** SIX6 + /POU4F2- hypothalamic organoids and **(J)** key developmental morphogens. **(K)** A conceptual diagram of key markers as diagnostic markers for different brain territories.

To explore the identity of SIX6-, SIX6 + and SIX6 + /POU4F2 + organoids we carried out RNA sequencing on day 65 organoids collected as biological replicates (*n* = 4) from pooled organoids (*n* = 3/replicate). Principal component analysis (PCA) using DESeq2 demonstrated that replicates from each category clustered together by organoid type (Figure 7A), thereby demonstrating the utility of our reporter system for identifying unique cell populations. Global differences were also analyzed by visualizing hierarchical clustered heatmaps based on sorting of the top 1,000 expressed genes in SIX6 + /POU4F2 + organoids (Figure 7B). Clustering of genes was apparent by organoid type. We also identified differentially expressed genes (DEGs) using EdgeR cross sample analysis. Using a stringent false discovery rate (FDR) pCutoff < 0.05 and log2FCcutoff > 1, we identified many genes that were up/down regulated between SIX6 + /POU4F2 + and SIX6- (1059up/837down), SIX6 + /POU4F2 + and SIX6 + (311up/499down), and SIX6 + and SIX6- (717up/535 down) organoids. A Venn diagram of DESeq2 normalized counts showed that while 14,562 expressed genes were common to all organoid types, 288, 322 and 380 genes were uniquely expressed in SIX6-, SIX6 + and SIX6 + /POU4F2 + samples, respectively, (Figure 7C). Thus, clear global differences in gene transcription existed by organoid type.

### Retinal Gene Expression in POU4F2 + Organoids

Differences between SIX6-, SIX6 + / POU4F2-, and SIX6 + /POU4F2 + organoids were visualized with volcano plots using a significance cutoff of log2FC > 1 and FDR < 0.05 (Figures 7D–F). The retina enriched markers *ATOH7*, *CDON*, *CRX, LHX2*, *OPTC, PAX6*, *RAX*, *RBP3, SEMA3F, SIX3*, *SIX6, THRB* and *VSX2* were upregulated in SIX6 + /POU4F2 + organoids relative to SIX6- organoids (Figure 7D; Supplementary Figure 8). This contrasts with *LMX1A*, *LMX1B*, *PITX2*, and *POU3F4* (*BRN4*), that were upregulated in SIX6- organoids. When SIX6 + /POU4F2 + organoids were compared to organoids expressing SIX6 + alone (Figure 7E), retina expressed genes (e.g., *SIX6*, *RAX*, etc.) were still detected; however, organoids expressing SIX6 alone differed in that they also expressed *SHH*, *OTP*, *NKX2-1*, *NKX2-2*, *OLIG1*, and *GHRH* (Figures 7E,F). SHH ventralizes the developing nervous system, including the retina (Echelard et al., 1993; Jensen and Wallace, 1997) and since SAG was used in our protocol to mimic SHH signaling, we explored whether retinal organoids were specified with dorsal (e.g., *TBX-2, -3*, and *-5*) and/or ventral (e.g., *VAX1*, *VAX2*, and *PAX2*) features. *TBX2*, *TBX3*, *VAX1*, and *VAX2* were all abundantly expressed in SIX6 + /POU4F2 + retinas at day 65 (Figures 7D,E), suggesting that both dorsal and ventral retina features were present. PAX2 is present in the developing optic stalk where it plays a key role in optic nerve formation. It was detected at significant levels in SIX6 + /POU4F2- and SIX6 + /POU4F2 + organoids (Figure 7G) suggesting a ventral fate. *FOXG1* which promotes optic fissure closure in mice through suppression of Wnt8b in the optic stalk, was also expressed (Smith et al., 2017). Heatmap analysis of significant retinal genes (*ATOH7*, *POU4F2, VSX1, VSX2*, and *VAX2*) showed high expression levels in SIX6 + /POU4F2 + organoids relative to other organoids (Figure 7G). *TBX5*, which is expressed in the human dorsal retina at embryonic stages (Sowden et al., 2001), however, was not detected. Lastly, the expression counts for key retinal genes was visualized by plotting gene counts (in counts per million) in each organoid category (Supplementary Figure 8). Collectively, this data suggested that SIX6 + /POU4F2 + organoids were bona fide retinas with both dorsal and ventral features which is consistent with previous reports (Hasegawa et al., 2016).

### Identification of Hypothalamic-Like Organoids

Outside the eye, SIX6 is expressed in the hypothalamus and pituitary, and the identification of two hypothalamic markers, *NKX2-1* and *GHRH*, suggested that SIX6 + organoids lacking POU4F2 might represent hypothalamus. Volcano plots comparing SIX6 + (with or without POU4F2) and SIX6- organoids showed that *SIX6* was the most highly differentially expressed gene (Figures 7D,F,G). *SIX6-GFP* + organoids had elevated *ALDH1A3*, *FZD5*, *HAP1*, *HDC*, *ISL1*, *GHRH*, *GPR50*, *LHX2*, *NKX2-1*, *NKX2-2*, *OTP, POMC*, *RAX*, *SIX3*, *SIX6*, *SST, TBX2*, *TBX3*, *SOX1, SOX6, RAB37*, and *VAX1* (Figures 7E,F,I; Supplementary Table 16 and Supplementary Figure 9). While their expression in the hypothalamus is well documented, some of these are also present in the retina (e.g., *LHX2*, *RAX*, *SIX3*, and *SIX6*). GHRH is a main neuroendocrine factor expressed in the arcuate nucleus of the hypothalamus (Plotsky and Vale, 1985) and was expressed in SIX6 + /POU4F2- organoids (Figure 7I). Histidine decarboxylase (HDC), which converts histidine to histamine in the hypothalamus (Eriksson and Mignot, 2009), was also abundant in SIX6 + /POU4F2- organoids as were transcripts for the orphan receptor *GPR50* (Figure 7I; Supplementary Figure 9), which regulates adaptive thermogenesis and torpor (Bechtold et al., 2012). *HAP1* coordinates with glucocorticoid receptors (GR) in the hypothalamus to regulate neuronal survival, food intake, body weight and stress (Fujinaga et al., 2004; Sheng et al., 2006). ISL1 controls *POMC* expression in the arcuate nucleus of the hypothalamus and was highly expressed in SIX6 + /POU4F2- organoids and to a lesser extent in SIX6 + /POU4F2 + retinas (Figure 7I; Supplementary Figure 9). Corticotropin-releasing hormone (CRH), which is synthesized in the hypothalamus to control ACTH release from the pituitary, was not detected in SIX6 + /POU4F2- organoids, but rather in SIX6- organoids (Figures 7F,H). The reason for this discrepancy is unknown. *NKX2-2* and *SHH* are typically found in adjacent stripes running along the antero-posterior axis (Dominguez et al., 2011). While *NKX2-2* transcripts were higher in SIX6- samples, they were also present in non-retinal SIX6 + samples (Figure 7H). *SHH*, on the other hand, was primarily found in SIX6 + /POU4F2- organoids. Several key pituitary markers were also observed in SIX6 + /POU4F2- organoids. Proopiomelanocortin (POMC), a precursor to adrenocorticotropin hormone (ACTH), is synthesized in the anterior pituitary, however, it is also made in the arcuate nucleus of the hypothalamus (Figure 7I; Supplementary Figure 9). Notably, we did not detect several important anterior (*CGA*, *CYP17A1, FSHB, GH1, GNRH, IGF-1, LHB, PRL*, and *TSHB*) and posterior (oxytocin and arginine vasopressin) pituitary expressed genes. In addition, *PITX* genes, which are required for pituitary development (Dutta et al., 2005), were only weakly expressed in SIX6 + /POU4F2- organoids. Two transcription factors with opposing activities in the developing pituitary are HESX1 and PROP1 (Perez Millan et al., 2016). *HESX1* is found in Rathke’s pouch, and while its presence was detected in SIX6 + /POU4F2- organoids and may be indicative of early pituitary growth, *PROP1* was not. Overall, this data confirmed that non-retinal SIX6 + /POU4F2- organoids expressed many hypothalamic markers; however, a lack of many other pituitary expressed genes does not support significant pituitary involvement.

### Organoids With Midbrain, Hindbrain, and Forebrain Gene Signatures

SIX6- organoids also exhibited gene expression profiles reminiscent of the midbrain-hindbrain (MBHB) region. Prominent among these genes were *FOXA1, FOXB1, HOXA3, HOXB6, HOXB8, IRX1, IRX2, IRX3, LMX1A, LMX1B*, and *PITX2* (Figures 7D,F,H; Supplementary Table 17 and Supplementary Figure 10). *LMX1A* and *LMX1B* are expressed in midbrain mesencephalic dopamine neurons (mesDA) and the cortical hem of the dorsomedial telencephalon and ventromedial diencephalon (VMD) (Andersson et al., 2006). *PITX2* which is normally required for mouse subthalamic nucleus and midbrain development (Martin et al., 2004), was abundant only in SIX6- organoids (Figures 7D,F,H). The HOXA-D family is an evolutionarily conserved group of homeobox transcription factors that imparts positional identity and compartmentalization along the body axis across many species from the MBHB boundary through the spinal cord (Figure 7H; Supplementary Figures 11A,B). Although some HOX expression was weakly detected in SIX6 + /POU4F2 + retina and SIX6 + /POU4F2- non-retina samples, the vast majority was in SIX6- samples (Supplementary Figure 11C) suggesting a hindbrain identity. Particularly abundant were *HOXA1-*7, *HOXB2-9*, *HOXC4-6*, and *HOXD3-8*. Although MBHB-like markers were most readily apparent in SIX6- organoids, forebrain-like genes were also detected. *EMX2*, which is strongly expressed in the dorsal telencephalon, posterior diencephalon, and developing cerebral cortex (Yoshida et al., 1997; Mathieu et al., 2002; Figures 7D,H), was present in SIX6- organoids. *FOXG1*, which is considered a telencephalic marker, was higher in SIX6 + /POU4F2- organoids (Figures 7F,I). This is surprisingly since a previous study showed that human ESC derived ventral diencephalon was *NKX2.1* + *FOXG1*– (Wang et al., 2015). FOXG1 also regulates *FGF8* which we observed to be higher in SIX6 + /POU4F2- and SIX6 + /POU4F2 + organoids relative to SIX6- organoids (Figure 7J). Although a strong MBHB profile existed, the presence of other anterior expressed genes suggested that SIX6- structures were not solely mid- or hindbrain-like. In future studies, it will be important to develop additional real-time markers to identify regionally patterned areas of the brain as they form.

### Morphogen Expression in Region Specific Tissues

Diffusible morphogens, including members of the BMP, WNT and FGF families, play critical roles in CNS patterning and RNA sequencing revealed unique morphogen patterns specific to each organoid type (Figure 7J). *WNT–2B*, –*3A*, –*4*, –*7B*, *–8B*, *–9A*, and *–10B* were most abundant in SIX6- organoids, consistent with their posterior expression across species (Hollyday et al., 1995; Ungar et al., 1995; Kunz et al., 2004; Fang et al., 2019; Poulin et al., 2020). Several, including *WNT3A* and *WNT10B* have prominent roles in MBHB patterning in other species (Lekven et al., 2003). *WNT9A*, whose chick ortholog is expressed in the ventral mesodiencephalic region of the brain and the dorsal midline of the anterior neural tube (fore-, mid-, hindbrain) in mice (Klafke et al., 2016), was also enriched in SIX6-organoids. Conversely, *WNT2B*, which regulates peripheral retinal growth (Cho and Cepko, 2006), was expressed in SIX6 + /POU4F2- and SIX6 + /POU4F2 + organoids. *DKK3* which antagonizes WNT signaling and has high expression in the eye (El Nakamura et al., 2007), was most highly expressed in SIX6 + /POU4F2 + retinal organoids. In mice, Fzd5 is expressed in the hypothalamus, thalamus, pituitary, optic vesicle and stalk, and the developing retina (Burns et al., 2008; Liu et al., 2008). In organoids we observed high levels of *FZD5* in SIX6 + /POU4F2 + retinas and lower expression in SIX6 + /POU4F2- hypothalamic organoids. On the other hand, several FGF’s (*FGF1, –3*, *–8*, *–9*, *–10*, and *–19*) were enriched in presumptive retinas. *FGF8* was only expressed in putative SIX6 + /POU4F2- and SIX6 + /POU4F2 + retinal organoids despite its reported expression in the MBHB *in vivo* (Figures 7E,F,H). FGF3 and -8 have been shown to control retinogenesis in chick and fish by regulating *Math5 (ATOH7)* (Martinez-Morales et al., 2005). *FGF12* and –*14* showed higher levels in SIX6- organoids and *FGF2*, –*11*, and –*13* showed high levels across all organoid types. SHISA2 is a WNT and FGF receptor antagonist that blocks key elements of their maturation and transport and is highly expressed in early chick retina (Hedge and Mason, 2008). Although weakly expressed in SIX6- organoids, *SHISA2* was over 20-fold higher in SIX6 + /POU4F2- and close to 40-fold higher in SIX6 + /POU4F2 + retinas, suggesting it’s importance for morphogen signaling in putative hypothalamus and retina organoids. While BMP–2 and –4 showed markedly higher levels in SIX6 + /POU4F2 + organoids, BMP7 was only enriched in SIX6- organoids. Together, these results demonstrate that organoids utilize unique combinations of morphogens to coordinate CNS patterning (Figure 7K).

## Discussion

Human retinal development spans a 9-month gestation period to form basic retinal anatomy and several years to form a mature fovea. Although development occurs slightly faster *in vitro*, this lengthy process poses technical hurdles for *in vitro* modeling. Furthermore, since retinal organoids develop as just one component of a more general anterior neural program the purity of organoid cultures is often called into question. Generation of a SIX6-GFP reporter line allowed us to visualize optic vesicles while also distinguishing them from SIX6- brain organoids, thereby providing a solution to reduce non-retina contaminants. By building a second SIX6-GFP/POU4F2-tdTomato dual reporter line, we further demonstrated that *SIX6* + organoids recapitulated structures resembling retina and hypothalamus. Several protocols already exist for the generation of human PSC derived hypothalamus (Wang et al., 2015; Huang et al., 2021) and anterior pituitary (Ozone et al., 2016); however, no live cell reporter currently exists for the early identification of such structures. Surprisingly, when we treated organoids with SAG at high doses analogous to what was used in those papers, we saw a loss of SIX6-GFP expression, not an increase as might be expected for hypothalamic growth. Revisiting these studies with SIX6-GFP iPSCs might shed additional light on this observation. Interestingly, POU4F2 is not exclusive to the retina as it is also known to be expressed in subpopulations of the superior colliculus (Byun et al., 2016), suprachiasmatic nucleus, lateral geniculate nucleus (Simmons et al., 2016), and cells of the marginal migratory stream that end up in the external cuneate nuclei and lateral reticular nuclei (Benzing et al., 2011). We occasionally detected sporadic POU4F2-tdTomato + cells in otherwise SIX6- organoids by live cell imaging, however, these levels were infrequent and difficult to reproduce, thus they were not studied further. We also observed some SIX6-GFP + organoids with only weak expression of POU4F2-tdTomato which we assumed to be low quality retinas. It is possible that poorly organized organoids with occasional POU4F2 + cells are non-retinal. Given that our approach isolated, and enriched organoids based on morphology and reporter fluorescence it is entirely possible that other unique populations, including the SC, LGN, SCN exist but were overlooked.

Media composition plays an important, yet often underappreciated, role in cell culture and SIX6-GFP iPSCs allowed us to optimize this in real-time. In general, PSCs thrive at higher osmolarity while neurons prefer the opposite (Bardy et al., 2015). The basal media used during stem cell differentiation (e.g., DMEM, DMEM/F12, Neurobasal) varies considerably between current retinal organoid differentiation protocols but tends to be lower in osmolarity. Initially, we hypothesized that using a higher osmolarity “stem cell-like” media would produce healthier spheroids and facilitate early neural induction, leading to improved retinas. However, we saw that it was the lower osmolarity that favored *SIX6* expression in retinal organoids. This is consistent with early studies in which neurons grown in Neurobasal, which has a lower osmolarity, had improved differentiation (Brewer et al., 1993). More recently, BrainPhys, which has even lower osmolarity, was shown to support improved electrical activity in cortical neurons (Bardy et al., 2015). Although not yet tested, it may confer a similar benefit for retinal organoids too.

Although O_2_ is historically known to influence cell physiology, a large gap exists in our understanding of how hypoxia affects routine cell culture (Taylor et al., 1978; Bottenstein and Sato, 1985). Our work on hypoxia in optic vesicle morphogenesis showed a synergistic role with hedgehog signaling. In support of a beneficial role for hypoxia is that mouse ES cells differentiated under hypoxia produced increased PR numbers (Bae et al., 2012; Garita-Hernandez et al., 2013; Chen et al., 2016). Precisely how hypoxia does this in the eye, however, is still not well understood. In cardiac cells, SHH is upregulated under hypoxia (Hwang et al., 2008) and a similar situation may exist in the eye. Proliferation, multi-potency, and differentiation of neural progenitors is significantly enhanced in low O_2_ and these can differentiate in the presence of various stimuli, such as BMP2 (Morrison et al., 2000; Studer et al., 2000; Santilli et al., 2010). In normoxia, p53 phosphorylation results in cell-cycle arrest, decreased proliferation, and differentiation of neural stem cells (NSCs) toward a glial lineage (Pistollato et al., 2007). Conversely, hypoxia enhances proliferation of NSCs through elevated cyclin D1 (Chen X. et al., 2010; Rodrigues et al., 2010). Soluble factors (e.g., erythropoietin and VEGF) are also induced by hypoxia and may enhance NSC proliferation (Youssoufian et al., 1993). VEGF alone can increase adult neurogenesis by influencing neuronal migration, survival, and axon guidance (Jin et al., 2002; Mackenzie and Ruhrberg, 2012). Lastly, HIF1-α-deficient mouse embryonic stem cell (ESC) derived embryoid bodies show increased neuronal characteristics accompanied by disruption of β-catenin signaling (Vecera et al., 2017). Mechanistic studies exploring the role of these factors could clarify the role of oxygen in human optic development.

Morphogens are important for normal development in that they can shape tissue development and body axis formation. Blocking WNT signaling at early stages promotes anterior neural development (Kiecker and Niehrs, 2001; Nakano et al., 2012), while activation at later stages contributes to optic cup development, dorsal retinal patterning and ciliary margin growth (Veien et al., 2008; Zhou et al., 2008; Hagglund et al., 2013). IWR-1e was used in prior work to block WNT during early stages of human optic cup formation (Nakano et al., 2012), however, the optimal timing was not clear. We were able to explore this question by using a SIX6 reporter at an early stage in neural development. Interestingly, at later stages retinal organoids exposed to the WNT agonist CHIR99021 formed sheets of continuous RPE, with very little retina (Kuwahara et al., 2015). Conversely, RPE induction by BMP4 followed by a short treatment with CHIR99021 (WNT activator) and SU5402 (FGFR inhibitor) led to reversal of both RPE and VSX2 + neural retina. The importance of WNT signaling was also highlighted by observations that VSX2 null retinas upregulate WNT signaling and retinal pigmented epithelium (RPE) layer duplication (Rowan et al., 2004; Capowski et al., 2016). Thus, VSX2 is a regulator of WNT signaling that maintains neural retina at the expense of RPE. Our own results showed that small molecule drugs modulating SHH and WNT were important in a time dependent fashion. Overexpression of Hh induces optic stalk expressed genes such as *pax2* and *vax1*, while inhibiting retinal genes such as *pax6* and *Rx* (Ekker et al., 1995; Macdonald et al., 1995; Hallonet et al., 1999). The presence of all four gene products in our retinas underscores the overall complexity of stem cell derived retinas and highlights the need for further study.

Although prior studies have shown that brain organoids can grow as light responsive structures (Quadrato et al., 2017), the converse is also true that retinal organoids can form alongside anterior forebrain structures (Meyer et al., 2009). In early studies of optic development, brain structures were primarily forebrain-like as opposed to the brain structures in our system that also included posterior brain regions. Although the reason for this difference is unclear, it is possible that the nutrient rich mixture in our current protocol does not select for anterior structures in the same way. The current study also differs from prior work in that we used stage and retina cell type specific reporters to sequentially tag SIX6-GFP and POU4F2-tdTomato + vesicles for the enrichment of retina tissues and this helped in the optimization of early optic development by hypoxia, WNT inhibition and SHH activation. Together, this approach made it possible to obtain large numbers of SIX6-GFP + vesicles, many of which developed into bona fide “retinas,” that were verified by RNA sequencing.

## Conclusion

In this study we developed single and dual color reporters for the longitudinal tracking of eye field and early retinal cells in 3D organoids. This approach allowed for the optimization of optic vesicle development by altering the timing and concentration of hypoxia, sonic hedgehog and Wnt signaling. Lastly, we leveraged dual reporter organoids to visually identify retina from non-retina organoids and in so doing were able to study transcriptional profiles of developing human retina, hypothalamus, and midbrain/hindbrain organoids.

## Data Availability Statement

RNA sequencing datasets are available as FASTQ files accessible at the Gene Expression Omnibus (GEO) database (https://www.ncbi.nlm.nih.gov/geo; GSE148168, GSE179260).

## Author Contributions

KW: conception and design, collection and assembly of data, data analysis and interpretation, manuscript writing, and final approval of manuscript. JC and SJ: collection and/or assembly of RNAseq data and final approval of manuscript. MJ: analysis of RNAseq data and final approval of manuscript. AO, NK, SR, CK, KE, and CB: collection and/or assembly of data and final approval of manuscript. JH, JP, and DG: collection of data and final approval of manuscript. RJ: data analysis and interpretation and manuscript writing. DZ: conception and design, data analysis and interpretation, manuscript writing, and final approval of manuscript. All authors contributed to the article and approved the submitted version.

## Conflict of Interest

The authors declare that the research was conducted in the absence of any commercial or financial relationships that could be construed as a potential conflict of interest.

## Publisher’s Note

All claims expressed in this article are solely those of the authors and do not necessarily represent those of their affiliated organizations, or those of the publisher, the editors and the reviewers. Any product that may be evaluated in this article, or claim that may be made by its manufacturer, is not guaranteed or endorsed by the publisher.

## Footnotes

1
http://rsb.info.nih.gov/ij/
