# Supplementary material for: CRISPR Generated SIX6 and POU4F2 Reporters Allow Identification of Brain and Optic Transcriptional Differences in Human PSC-Derived Organoids
Source: Front Cell Dev Biol. 2021 Nov 16;9:764725. doi: 10.3389/fcell.2021.764725 (PMC8635054; doi:10.3389/fcell.2021.764725)
Supplement: Supplementary file 1 [file Data_Sheet_1.PDF]

Supplemental Material (Wahlin et al.)

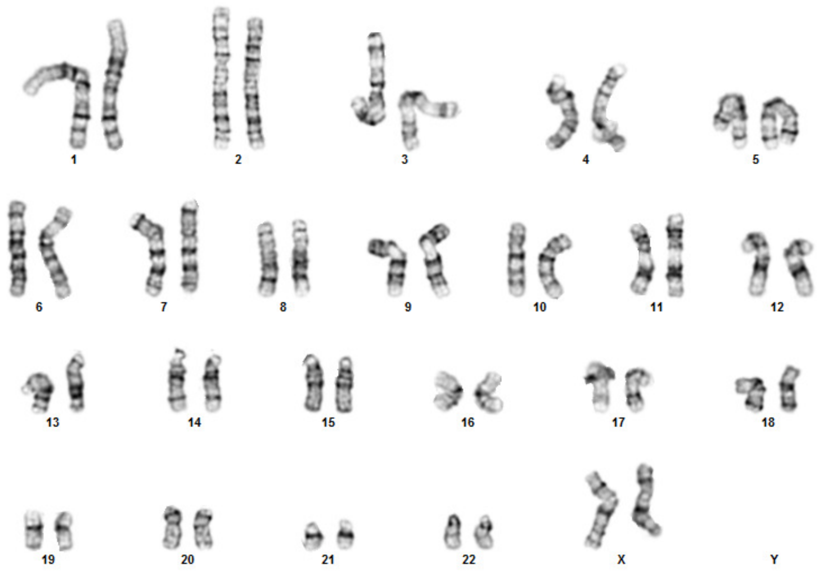

Fig. S1. G-banding karyotype analysis. Cytogenetic analysis was performed on twenty G-banded metaphase cells from human cell line IMR90.4-S34 towards the end of experiments. Eighteen cells demonstrated a normal female karyotype; however, two cells demonstrated a possible abnormal karyotype with possible trisomy 12.

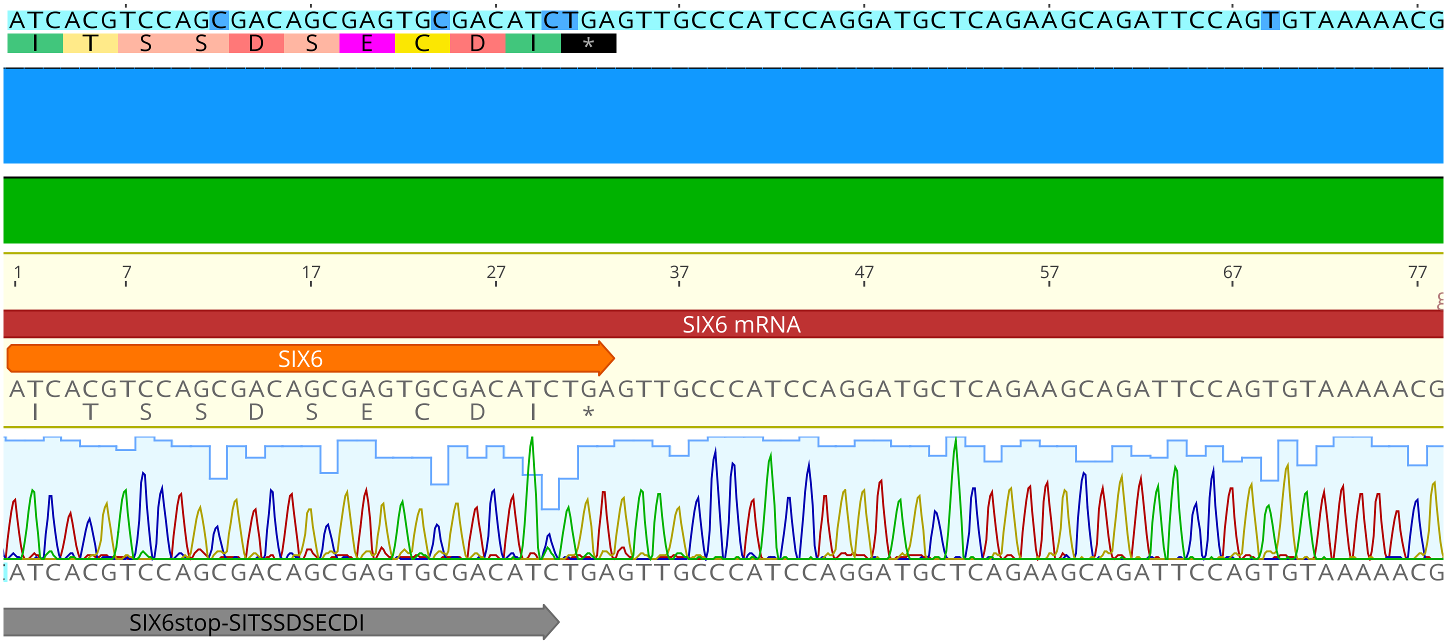

Fig. S2. Sanger sequencing of the unmodified SIX6 allele. To rule out unwanted mutagenesis in the unmodified SIX6 allele in the SIX6-p2A-eGFP single reporter line Sanger sequence was performed on gel extracted PCR products.

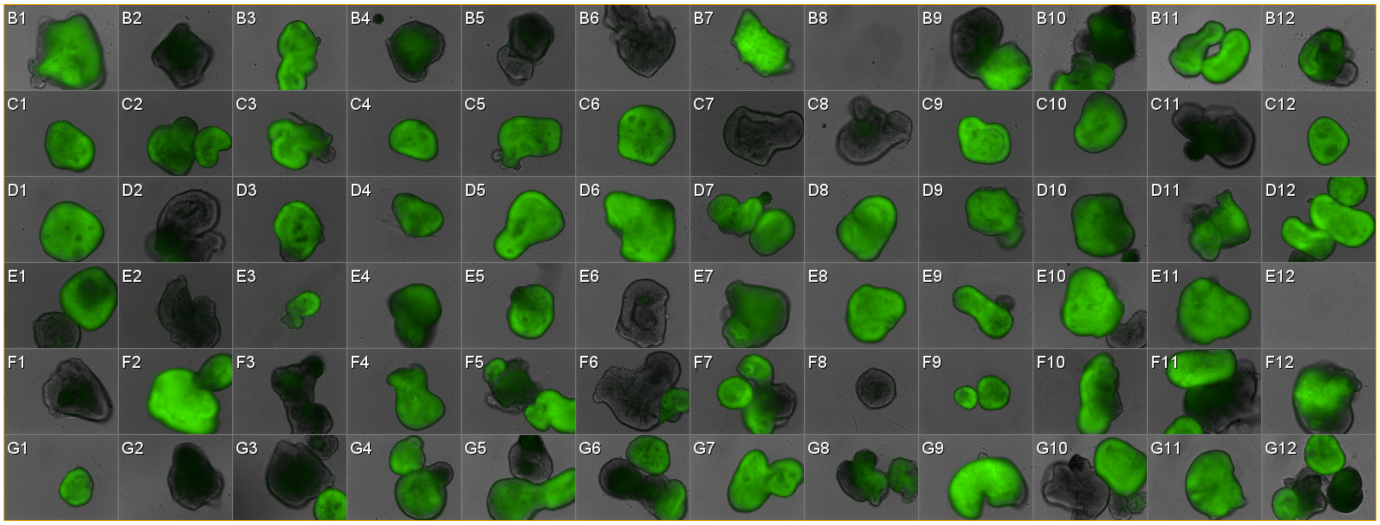

Fig. S3 Organoids with or without SIX6-GFP fluorescence at 27 days. Organoids were manually dissected and transferred to individual wells on a 96 well plate. This enrichment step allowed for the isolation of SIX6-GFP positive or negative organoids for further study by RNAseq at day 35.

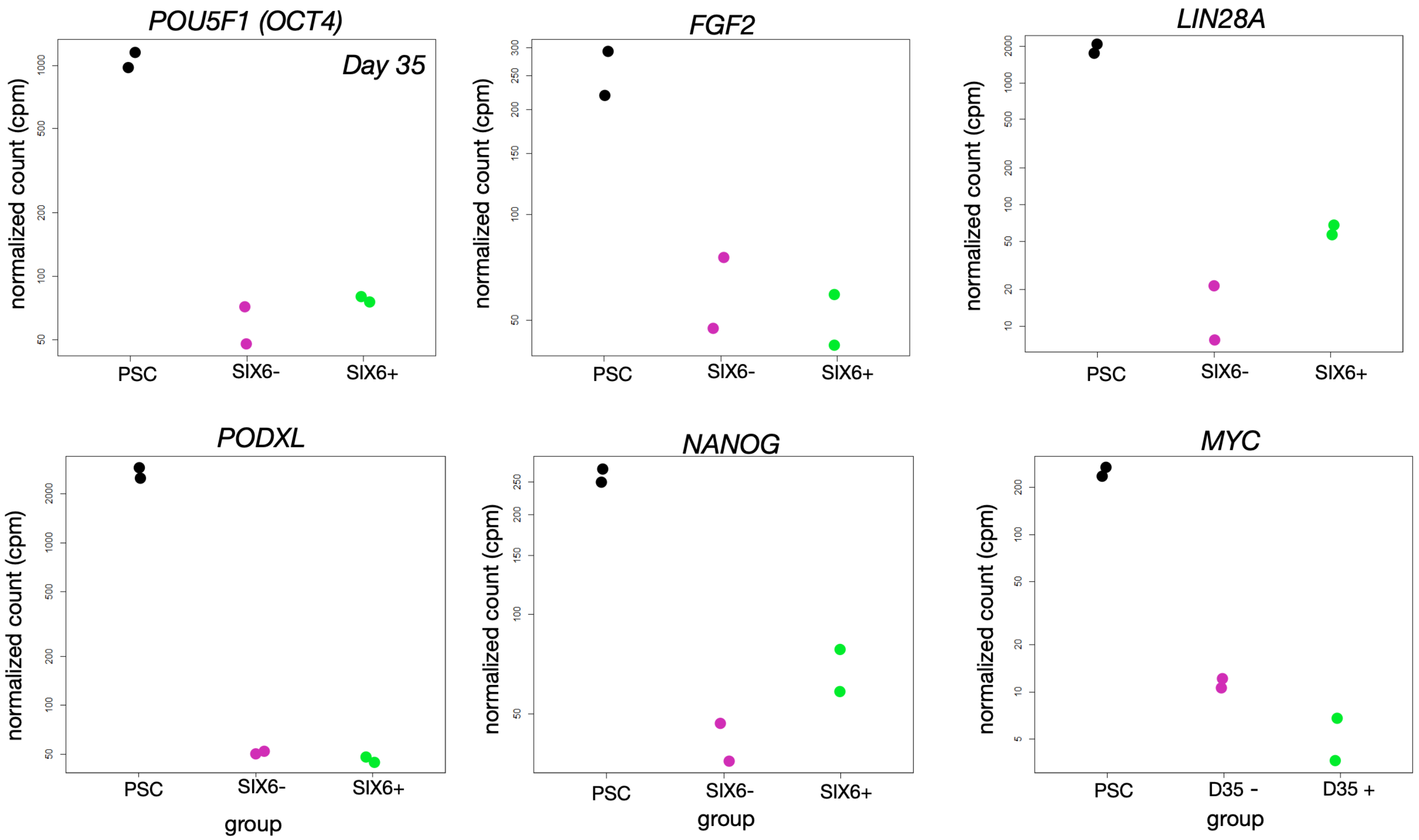

Fig. S4. Stem cell genes that are highly expressed in undifferentiated pluripotent stem cells (PSCs) relative to day 35 SIX6-GFP negative (SIX6-) and positive (SIX6+) organoids. Normalized counts from DESeq2 are displayed in counts per million.

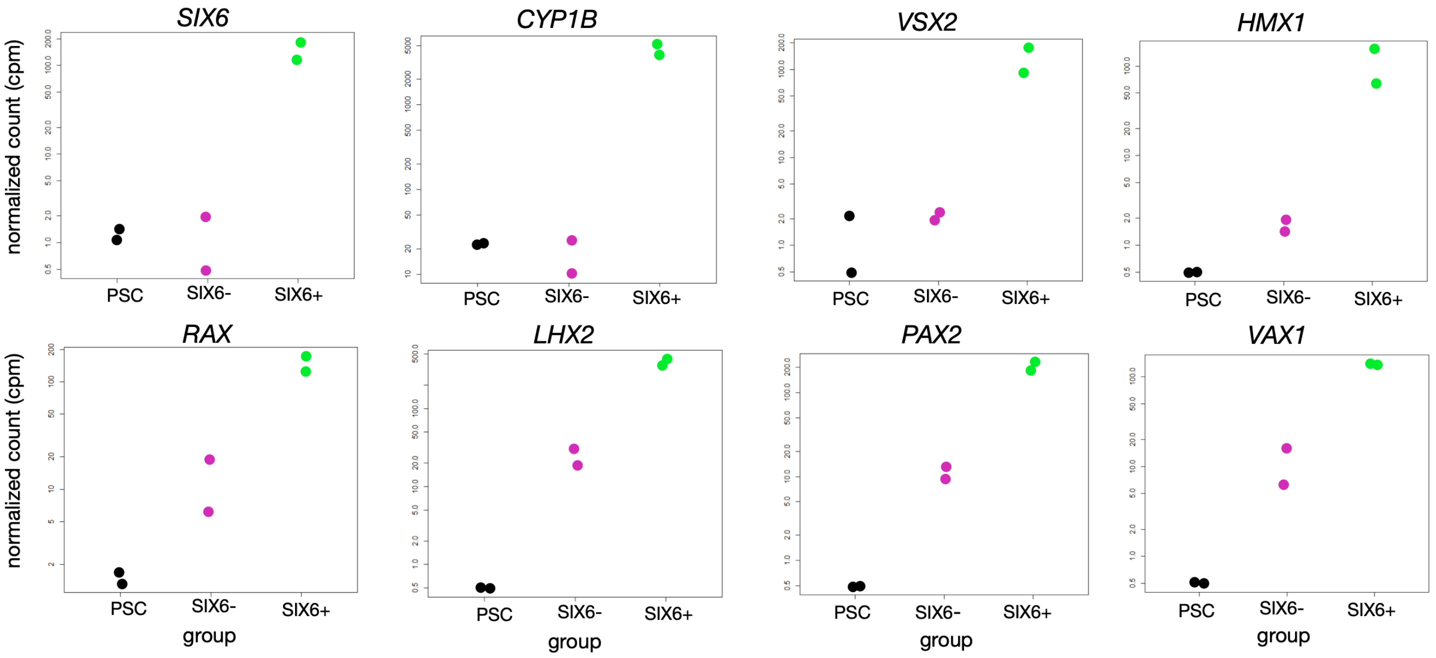

Fig. S5. Optic vesicle genes that are highly expressed in day 35 SIX6-GFP positive (SIX6+) early retinas relative to undifferentiated pluripotent stem cells (PSCs) and SIX6 negative (SIX6-) organoids. Normalized counts from DESeq2 are displayed in counts per million.

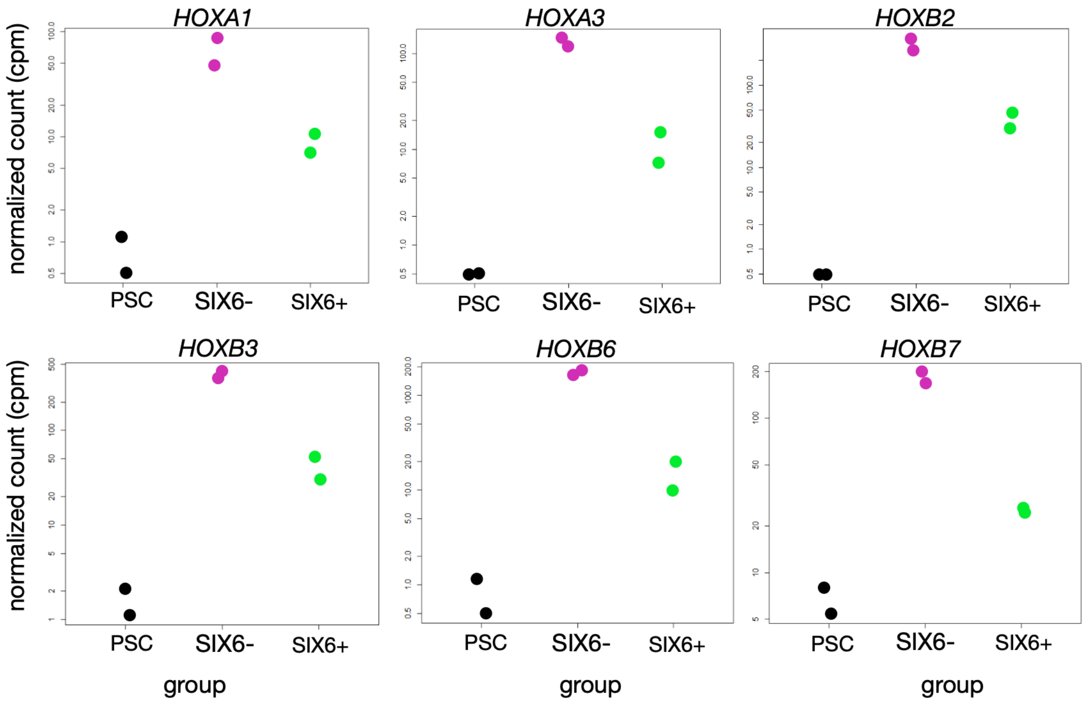

Fig. S6. Genes that are highly expressed in SIX6-GFP negative (SIX6-) organoids relative to undifferentiated pluripotent stem cells (PSCs) and day35 SIX6-GFP positive (SIX6+) organoids. Normalized counts from DESeq2 are displayed in counts per million.

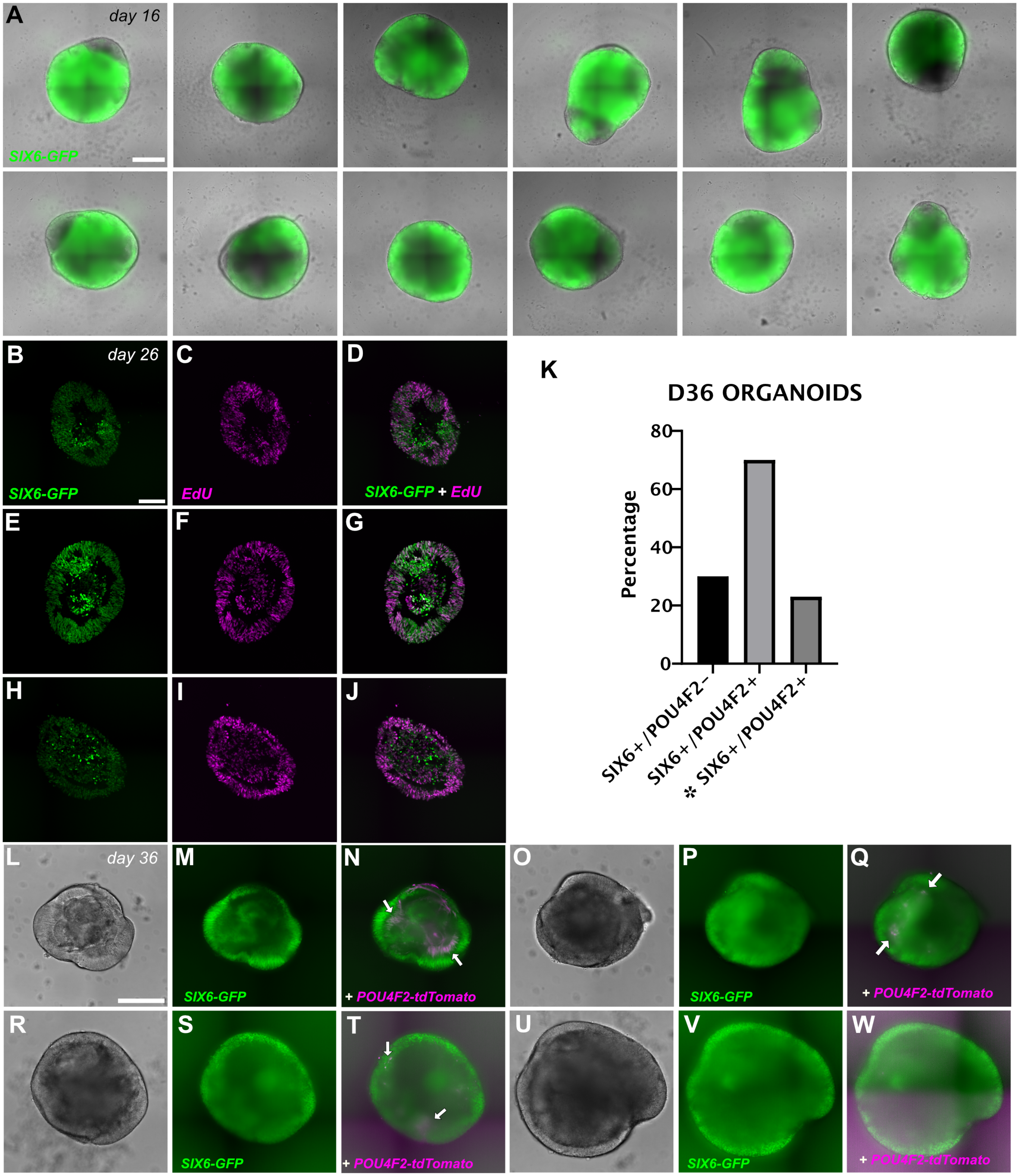

Fig. S7. Assessment of *SIX6-GFP*/*POU4F2*-tdTomato organoids. (A) Pre-cut replicate organoids with consistent *SIX6*-GFP expression across large areas of the organoids. (B-J) Three day 26 organoids processed for 18 hours with the Click-&-Go EdU 647 cell proliferation assay showing robust incorporation of EdU labeling across *SIX6*+ organoids. (K) Quantification of organoids expressing *POU4F2*-tdTomato that express *SIX6*-GFP and either lack *POU4F2*-tdTomato or

show *POU4F2*-tdTomato expression with high levels of organization (retinas) or low quality and low expression of POU4F2-tdTomato (retinal identity unknown). Representative images of (L-N) high-quality brightfield and fluorescence images in *SIX6*-GFP/POU4F2+ organoids, (O-T) low-quality *POU4F2+* organoids, and (U-W) organoids with non-existent levels of *POU4F2*-tdTomato. Scale (A) = 500 µm, (B-J) = 100 µm, (L-W) = 300 µm.

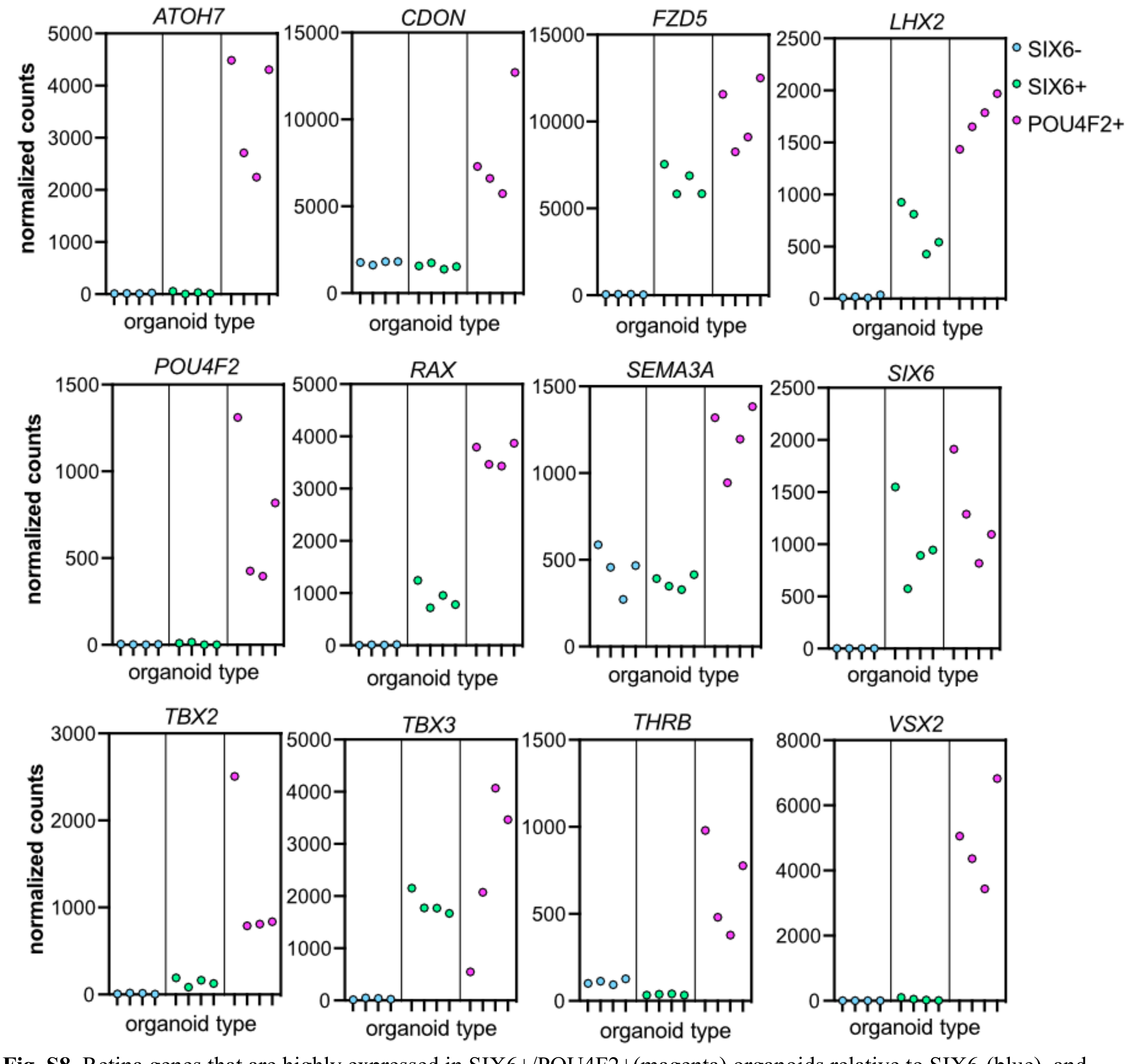

Fig. S8. Retina genes that are highly expressed in SIX6+/POU4F2+(magenta) organoids relative to SIX6-(blue), and SIX6+/POU4F2-(green) organoids. Normalized counts from DESeq2 are displayed in counts per million (cpm).

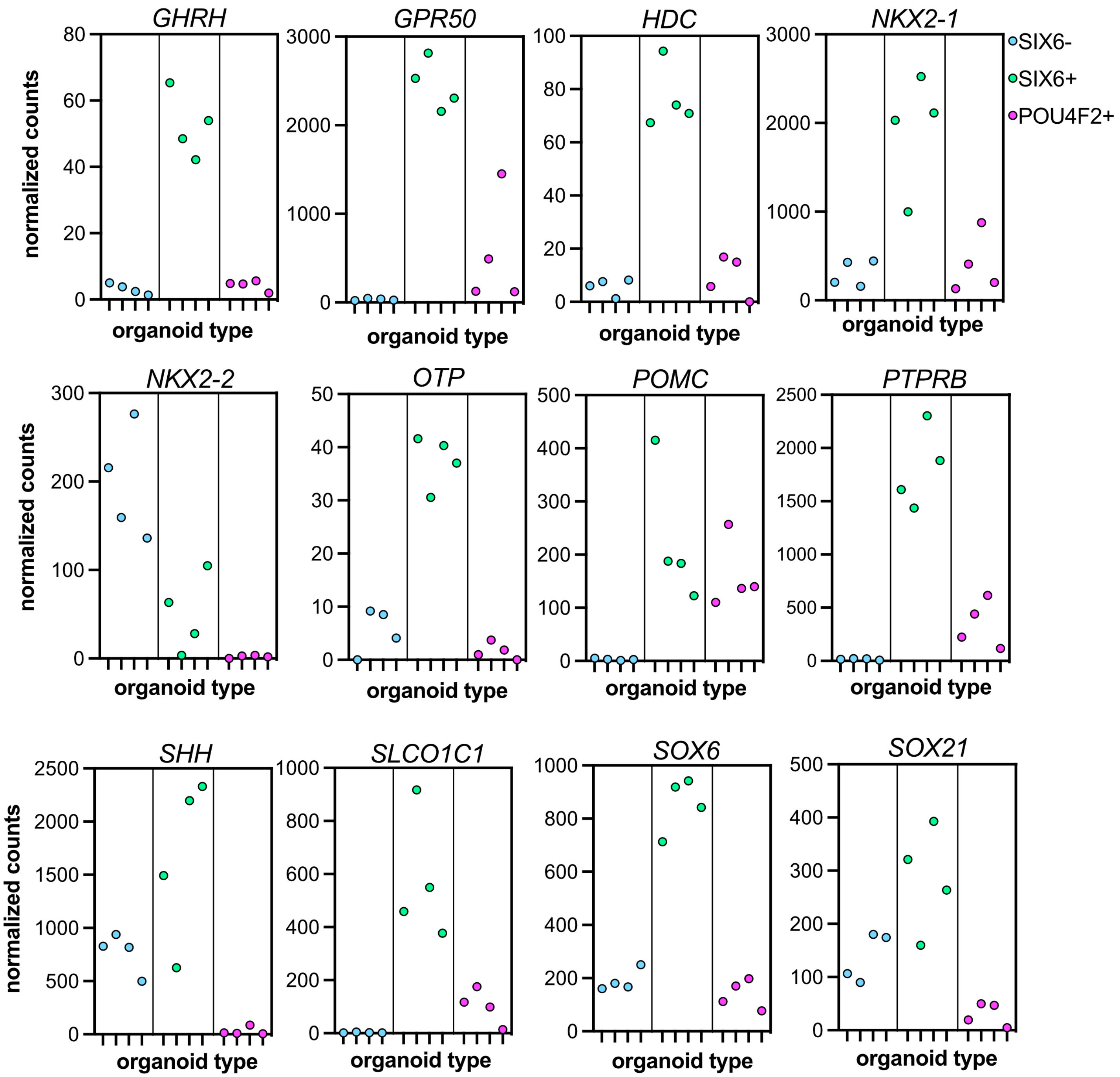

Fig. S9. Hypothalamic genes that are highly expressed in SIX6+/POU4F2-(green) organoids relative to SIX6-(blue) and SIX6+/POU4F2+(magenta) organoids. Normalized counts from DESeq2 are displayed in counts per million.

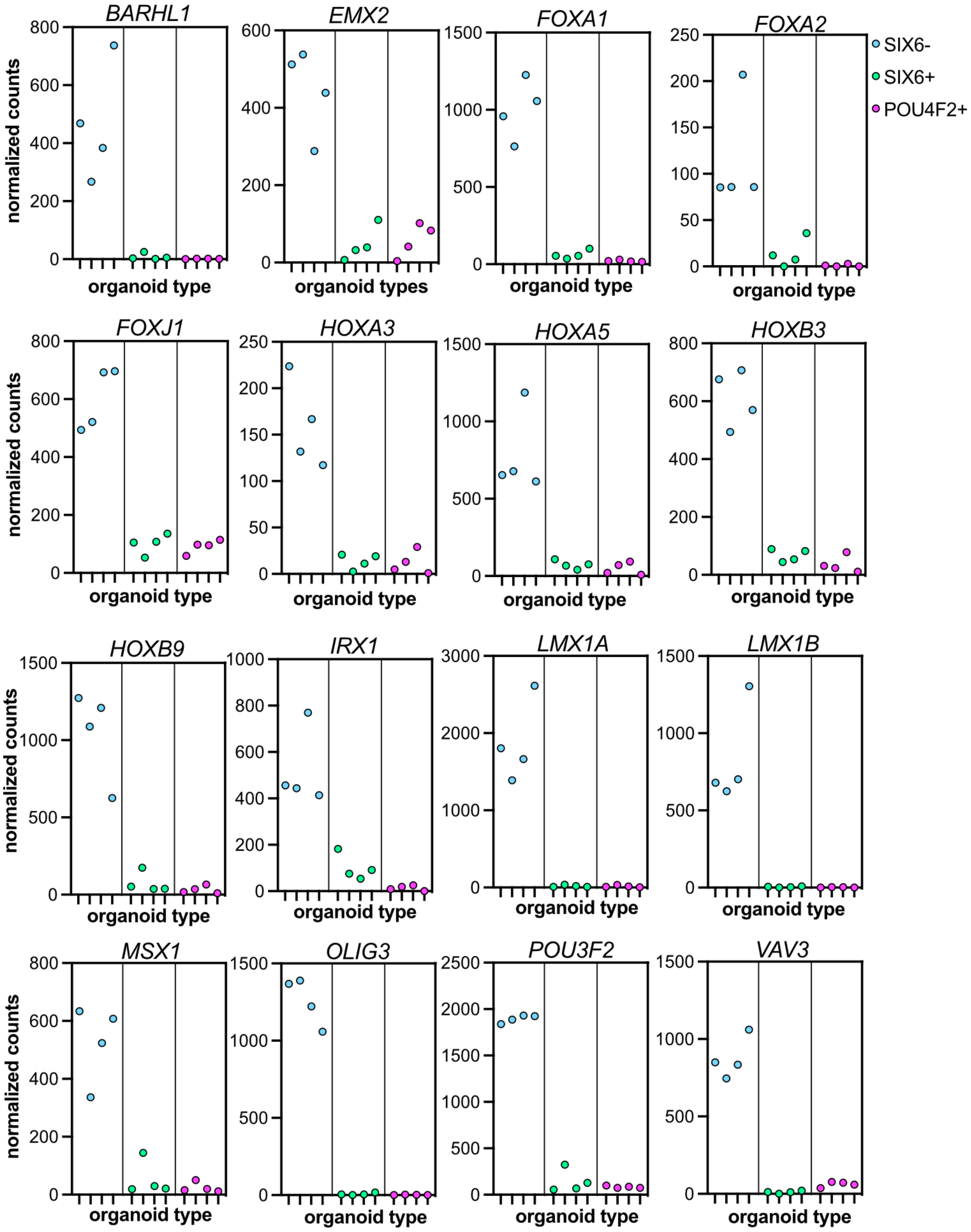

Fig. S10. Midbrain-hindbrain like genes that are expressed in SIX6-(blue) organoids relative to SIX6+(green), and SIX6+/POU4F2+(magenta) organoids. Normalized counts from DESeq2 are displayed in counts per million.

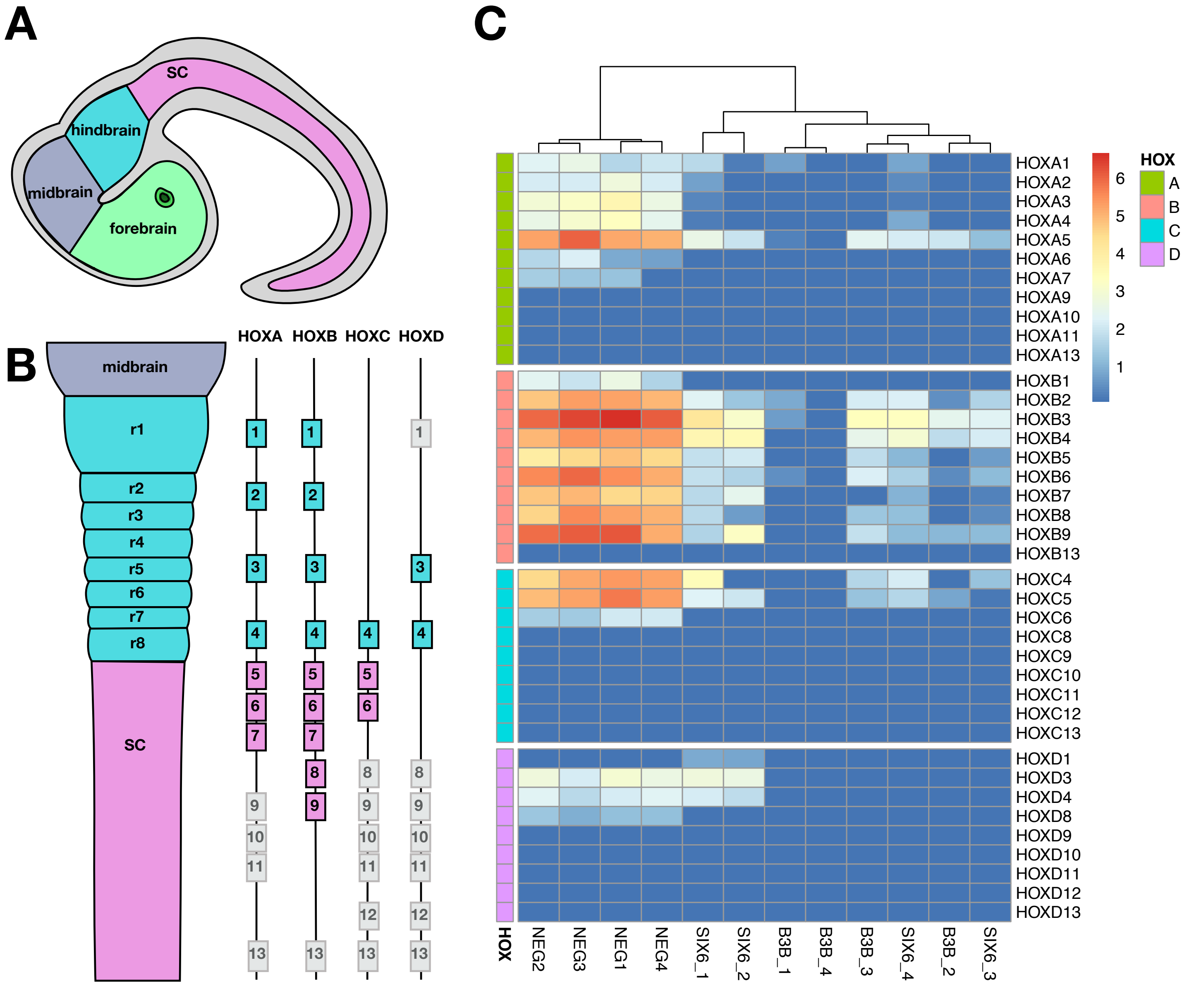

Fig. S11. HOX gene profiles in SIX6- organoids. (A) Structural organization of the developing embryo and (B) the relative spatial organization of the HOXA-D family in the developing human nervous system. (C) A clustered heatmap of HOX family genes expressed in SIX6- (NEG), SIX6+/POU4F2- (SIX6), and SIX6+/POU4F2+ (B3B) replicates.

Human 3D Retina Organoid Protocol v2.0

This protocol gives rise to hPSC derived optic vesicles that can be generated by 12 days. Retinal differentiation by the forced aggregates approach allows for development of all retinal types including photoreceptors. RPE tissues, anterior neural tissues (and cornea) can also be generated by this approach but requires manual excision. These structures can be maintained in excess of 300 days and likely well beyond.

Reagents

mTeSR1

- add 5X supplement (-80C) to 400ml's of basal medium (4C; from Stem cells tech) and aliquot 45 ml's of this solution into 50ml tubes. ** note- mix medium well.

E6 stock medium (50X) - 100ml (10 aliquots)

- add 36.2ml 7.5% **$NaHCO_3$ (7.5g/100ml)**
- add water to 100ml * not critical
- add 97mg **insulin** **
- add 53.5mg **holo-transferrin**
- add 320mg **L-ascorbic acid** (note- will become cloudy),
- add **sodium selenite** (5μl of 1,000,000x [14mg/ml] stock).
- will be filtered later
- aliquot 10ml into 15ml tubes.
- Store in designated box in -80°C.
- ** Insulin is soluble in water for Roche (check solubility if using another vendor).

OV1 medium (BE6.2)-250ml

- 10ml of **E6** stock
- 5.0ml **B27 (-Vit A)**
- 2.5 ml **Glutamax** (100X)
- 2.5 ml **NEAA** (100X)
- 2.5 ml **Pyruvate** (100X)
- bring to 250ml **DMEM (high glucose; Life Tech #11965)**;** filter sterilize
- keep fresh for up to 2 weeks.

***NOTE- Fluorobrite DMEM (#A18967) - has no glutamine so if using make sure to add 2 ml's 100X glutamine per 100ml's of medium.*

OV2 medium (LTR medium)-500ml

- 125 ml **F12 (cat#11765)**
- 50ml's 100% **FCS**.
- 10 ml's **B27** (regular)
- 5 ml's **Glutamax** (100X)
- 5 ml's **NEAA** (100X)
- 5 ml's **pyruvate** (100X)
- 500μl's **taurine** (1,000X - 1M stock)
- bring final volume to 500 with **DMEM - high glucose (cat#11965)** and filter sterilize.

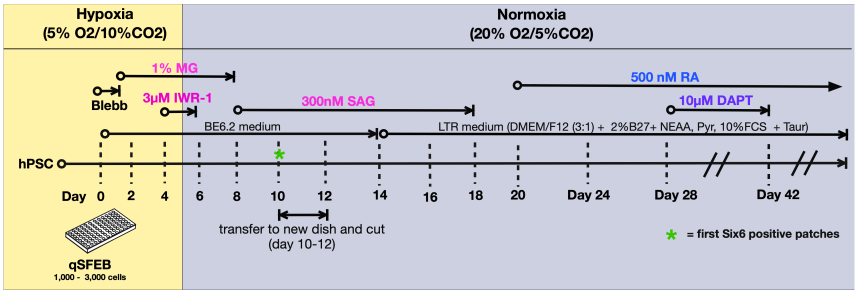

Stem Cell Maintenance:
Stem cells are maintained feeder free on 1% Matrigel-GFR (#354230; BD Biosciences) coated dishes at 37°C under hypoxia (10% CO_2/5%O_2) in mTeSR1 (Stem Cell Technologies). Cells were single cell passaged every 4-6 days with Accutase (#A6964; Sigma) and resuspended in mTeSR1 plus 5μM (-) blebbistatin (B; #B0560; Sigma) as previously described[8]. 5,000 cells are reseeded in mTeSR1 plus 5 μM (-) blebbistatin (B) into a 35 mm dish.

Priming for Differentiation: PSCs are prepared for differentiation by passaging cells at higher density so that resulting colonies are still rather small but more abundant. 20,000 cells per 35 mm dish are sufficient for this purpose. Allow cells to grow for ~3 days before initiating organic cultures.

✪ *note* - do not allow colonies to grow too large since Accutase will not work as well.

Forced Aggregate Differentiation:

(1) **Day 0 (day of passage)** - Passage PSCs in 1ml Accutase for 10 minutes.

Gently, triturate up/down with P1000 3X and transfer to 2ml mTeSR1 containing blebbistatin to quench. Spin at 80xg for 5 minutes and resuspend in mTeSR1 containing 5 μM blebbistatin(B). Add 1,000 cells in 50 μl's of mTeSR1+B (for each 96 well plate prepare 100,000 cells to 6 ml's of mTeSR1+B) into each well of a non-adherent round (U-Shaped) bottom **96-well plate (polystyrene U-bottom; Greiner)**. A Multi-channel pipette is ideal for this. **Place back in hypoxic incubator.**

✪ *note* - organoids should form a single sphere after 24 hours. To facilitate early sphere formation and prevent clumping tap the sides of the plate 10-15 times one hour after seeding cells into the well.

Reagents

mTeSR1

- add 5X supplement (-80C) to 400ml's of basal medium (4C; from Stem cells tech) and aliquot 45 ml's of this solution into 50ml tubes. ** note- mix medium well.

E6 stock medium (50X) - 100ml (10 aliquots)

- add 36.2ml 7.5% **$NaHCO_3$ (7.5g/100ml)**
- add water to 100ml * not critical
- add 97mg **insulin** **
- add 53.5mg **holo-transferrin**
- add 320mg **L-ascorbic acid** (note- will become cloudy),
- add **sodium selenite** (5µl of 1,000,000x [14mg/ml] stock).
- will be filtered later
- aliquot 10ml into 15ml tubes.
- Store in designated box in -80°C.
- ** Insulin is soluble in water for Roche (check solubility if using another vendor).

OV1 medium (BE6.2)-250ml

- 10ml of **E6** stock
- 5.0ml **B27 (-Vit A)**
- 2.5 ml **Glutamax** (100X)
- 2.5 ml **NEAA** (100X)
- 2.5 ml **Pyruvate** (100X)
- bring to 250ml **DMEM (high glucose; Life Tech #11965)****; filter sterilize
- keep fresh for up to 2 weeks.

***NOTE- Fluorobrite DMEM (#A18967) - has no glutamine so if using make sure to add 2 ml's 100X glutamine per 100ml's of medium.*

OV2 medium (LTR medium)-500ml

- 125 ml **F12 (cat#11765)**
- 50ml's 100% **FCS**.
- 10 ml's **B27** (regular)
- 5 ml's **Glutamax** (100X)
- 5 ml's **NEAA** (100X)
- 5 ml's **pyruvate** (100X)
- 500µl's **taurine** (1,000X - 1M stock)
- bring final volume to 500 with **DMEM - high glucose (cat#11965)** and filter sterilize.

100ml's of medium.

OV2 medium (LTR medium)-500ml

- 125 ml **F12 (cat#11765)**
- 50ml's 100% **FCS**.
- 10 ml's **B27** (regular)
- 5 ml's **Glutamax** (100X)
- 5 ml's **NEAA** (100X)
- 5 ml's **pyruvate** (100X)
- 1.25ml's **taurine** (400X - 0.4M stock)
- bring final volume to 500 with **DMEM - high glucose (cat#11965)** and filter sterilize.

✪ *note* - although organoids are maintained under hypoxia for the initial stages of differentiation, cells are exposed to ambient O2 for approximately 10-15 minutes during feeding.

(2) **Day 1 (24hrs)** - Add 50µl's of BE6.2 containing 2% (v/v) **matrigel** (final 1%) in **BE6.2**. Blebbistatin is no longer added. **Place cells back into hypoxic incubator.** You are now at 100 µl's of medium and with each feeding, the concentration of mTeSR1+B becomes reduced.

✪ *note* - Matrigel and IWR-1e are added at 2X concentration on day 1 since 50µl of media without these components are already in the well.

✪ *note* - make sure BE6.2 medium is ice-cold before adding Matrigel. Once Matrigel has been added then warm BE6.2 to 37C (10-15 minutes)

(3) **Day 2 (48hrs)** - Add 50µl's (now you are at 150µl) of BE6.2 containing 1% (v/v) **matrigel**.

(4) **Day 3 (72hrs)**- Add 50µl's (volume is now 200µl) of BE6.2 containing 1% (v/v) **matrigel**. You are now at 200 µl's.

✪ *note* - the high volume can makes it easy to splash on the surface of the plate. If liquid draws up to the surface, aspirate it away by vacuum.

(5) **Day 4- 50% daily exchange** - remove 100 µl's of medium and replace with 100 µl's fresh medium containing **matrigel** plus **6µM IWR-1e Wnt** inhibitor (final 3µM final after 50% dilution).

✪ *note* - During 50% exchanges it is important to mix the sample well. Using a multi-channel pipette, slowly draw up 100 µl's of media slowly and then expelling it with a little more force. Repeat this with all wells. Do not suck up organoids during mixing.

(6) **Day 5- 50% daily exchange** - remove 100 µl's of medium and replace with 100 µl's fresh medium containing **matrigel** plus **IWR-1e** Wnt inhibitor. **Transfer to normoxia (20%O2/5%CO2).**

(7) **Day 6- 50% daily exchange** - remove 100 µl's of medium and replace with 100 µl's fresh medium containing **matrigel**. No more IWR-1 treatment.

(8)**Day 7- 50% daily exchange** - remove 100 µl's and replace with 100 µl's BE6.2 + **matrigel**.

(9) **Day 8- 50% daily exchange** - remove 100 µl's of medium and replace with 100 µl's fresh medium. Add **600nM SAG on day 8 to the 100µl already in well (300nM SAG final).** No more Matrigel.

(10) **Day 10**- Using a wide-bore P1000 pipet tip transfer organoids to a 15 ml tube and rinse organoids 3X with 10ml's of DMEM to prevent sticking of organoids. Transfer organoids to a 35mm dish and then using an inverted microscope cut organoids using electrolytically sharpened tungsten needles. Isolated vesicles can

then be transferred to 10cm non-tissue culture treated polystyrene petri dishes in 20 ml BE6.2 plus **300nM SAG**.

✪ *note* - days 10-14: manually dissect out neural vesicles based on morphology. If vesicles are beginning to lose their morphology you should excise vesicles as soon as possible. For details on producing electrolytically sharpened tungsten needles refer to Supplemental materials in Wahlin et al, 2017 (https://doi.org/ 10.1038/s41598-017-00774-9).
✪ *note* - recommended -limit aggregates to improve results (range 24-36 organoids) per 10cm plate. Keeping this density low is very important - high density cultures typically have poor morphology.

(11) **Day 12** - 75% media exchange with BE6.2 + **300nM SAG**.

(12) **Day 14** - 50% media exchange with **LTR medium** + **300nM SAG** + **1mM Taurine**.
✪ *note - using a gradual 50% media exchange will improve overall health of organoids.*

(13) **Day 16 -** 75% media exchange in **LTR** + **300nM SAG** + **1mM Taurine.**
✪ *note - typical feeding for all steps forward are ~75% exchange.*

(14) **Day 18** - Transition to **LTR medium + 1mM Taurine** without SAG.

(15) **Day 20** - Transition to **LTR medium + 1mM Taurine** + **500nM all-trans retinoic acid (ATRA).**

✪✪ *note- feed every other day from here on with LTR+taurine+ATRA.*

(16) **Day 28** - Add **10µM DAPT** until Day 42.

✪✪ *note- recommended - discard aggregates that that appear disorganized or brown/necrotic.*

Aliquoting and Handling of Reagents:

All-trans retinoic acid (**ATRA**) (20,000x- 10 mM stock; Sigma #R2625-50MG) ✪ light sensitive.
For a 10 mM stock add 1.66 ml of DMSO to 5 mg of ATRA or 16ml's of DMSO to 50mg. Aliquot and store at -80C away from light for up to 4 months. Working concentration = 500nM: Add 2 µl's of 20,000x stock per 40 ml's of LTR medium.
note - ATRA is extremely sensitive to UV light, air, and oxidizing agents. Solutions of RA in pure organic solvents, when stored in the dark, are reasonably stable whereas aqueous solutions deteriorate quickly.

(–)-Blebbistatin (2000x-10mM stock; Sigma B0560-1MG). ✪ light sensitive.
Dissolve 1mg blebbistatin into 340 µl DMSO, aliquot into 5, 10 and 20µl aliquots (store at -80C). Working concentration = 5 µM.

DAPT - (1,000x-10mM stock; Calbiochem# 565770-5mg; FW= 432.5)
Add 5mg DAPT to 1.16 ml DMSO. Store aliquots at -80oC. ✪ protect from light. Working concentration = 10 µM.

IWR-1-endo Wnt Antagonist) (1,000x - 3 mM stock; EMD Millipore/Calbiochem #681669-10MG; **FW-409.4) ✪- light sensitive**).
To make, add 10 mg to 8.1ml DMSO. Stock solutions are stable for up to 3 months at -80C. * protect from light. Working concentration = 3µM.

Matrigel (GF reduced) - each lot has a different concentration so check with BD biosciences each time. Check here for the concentration of your lot: http://regdocs.bd.com/regdocs/searchCOA.do
✪ **critical-** handling of Matrigel is very important and the temperature should NEVER go above 4oC. To ensure that the temp is maintained at a cold temperature the following things can be done:

- **Aliquoting Matrigel stocks:**

(1) Thaw 10ml bottle overnight on wet ice @ 4oC before aliquoting. ✪✪ do not place Matrigel in 4oC since it will gel.
(2) Always keep tubes on ice and never let warm up. To keep pipet tips cold prior to dispensing materiel I recommend

keeping a tube of ice cold DMEM based basal media or PBS handy so you can pipet up/down cold media. Do this

directly before pipetting matrigel.
(3) Directly after dispensing MG aliquot 1mg (usually around 100µl's) into pre-chilled 1.5 ml screw cap tube add tube to

ice until aliquoting is done. Store at -80oC for long term.
- **To use for stem cell maintenance**: Thaw matrigel aliquot on wet ice (in cold water). Add 12 mL cold DMEM/F12 (or DMEM) to 15 ml conical tube and place on ice. When matrigel has thawed add the 1mg (~100ul's) sample to 12 ml ice cold DMEM/F12 (~0.1mg/ml final). To keep pipet tip cold while transferring, take up 1 ml of ice cold DMEM from the 12 ml tube, pipet up/down 3 times, dispense into the MG tube, then transfer it right back into the 12 ml tube. Invert mix 3x and dispense 1ml per well into 6 well plate (~.083µg/ml). Coat with matrigel for > 8hrs but no more than 2 days at 37C. Don't use plates older than 2 days.
- **To use for organoid formation**: For spheroids, use % vol/vol where 100 µl's of Matrigel is added to 10 ml's optic vesicle medium (BE6.2) to make a final 1%. Note- on the first day of this protocol you will add 200 µl's / 10ml since you are adding this to an equal volume of cell medium.

Smoothened agonist SAG (1,000X stock - 300µM; FW:599); 1MG vial
- dissolve 1MG into 5.56 ml DMSO. Make 100µl aliquots and store at -80C. Stock solutions are stable for up to 6 months at -20°C so should last longer in -80C.Working concentration = 300nM.

Sodium chloride (NaCl; 250X stock)

To make a 250x stock solution:
- add 21.9g NaCl to 100ml cell culture grade water. It will go into solution and be clear without heating. - note- cannot be filtered a this stage. Filter sterilize once it is added to the BE6.2 medium anyway.

Taurine - working 1mM (400X stock - 400mM; 50mg/ml)(Sigma - T-8691; MW=125.15l)

- add 500mg to 10ml's TC grade water. * - freeze these as 1.25ml aliquots and store in -80C. Use the entire 1.25ml aliquot per 500ml bottle of LTR.

Catalog #	Description	Size	Company
Cell culture			
A6964-100ml	accutase	100ml	Sigma
86.1252.01	aspirating pipets - 2ml	100/pk	Starstedt
17504044	B27 supplement	10ml	Gibco
12587010	B27 supplement w/out Vitamin A	10ml	Gibco
11765	F12, with Glutamine	500ml	LifeTech
11965-092 (1)<br>11965-118 (10)	D-MEM (1X), liquid (+ 4.5g/L glucose, + L-glutamine, no pyruvate, no HEPES, + phenol red);	500ml<br>10x500ml	LifeTech
11360-070	100X pyriuvate (100mM)	100ml	LifeTech
35050061	100X Glutamax	100ml	LifeTech
11140-050	100X MEM-NEAA	100ml	LifeTech
16140071	FBS - Heat inactivated (Qualified)	500ml	LifeTech
354230	Growth Factor Reduced BD Matrigel™ Matrix<br>* note- this is now offered through Thermofisher.	10ml	BD Biosciences
89107-632	100x25mm tall bacterial grade petri dishes for long term maintenance of retinal cups.	10cm	VWR

Catalog #	Description	Size	Company
#650180 / Item No.: 5665-0180 (USA Sci)	96-well polystyrene Cellstar clear TC plate with lid, U bottom, sterile, individual,100/cs	100/pk	Greiner
#656171 / Item No.: 5665-6171	Lid with condensation ring	100/pk	Greiner USA Scientific
#650161 / Item No.: 5665-0161 (USA Sci)	96-well PS plate, U bottom, sterile, 2/sleeve	100/pk	Greiner / USA Scientific
SCGP00525 or #506231 U.supply	Steriflip 50ml filters	25/pk	Millipore
#05850	mTeSR1	500ml	Stemcell Technologies
Growth factors and small molecules			
R2625-50MG	ATRA, Tretinoin, Vitamin A acid, all-*trans*-Retinoic acid	50mg	Sigma
B0560-1MG	(-)-blebbistatin	1mg	Sigma
565770-5mg<br>565770-10mg * more economical	DAPT<br>N-[N-(3,5-Difluorophenacetyl-L-alanyl)]-S-phenylglycine t-Butyl Ester- ‘gamma-secretase inhib IX’	5mg<br>or<br>10mg	calbiochem
681669	IWR-1-endo (Wnt Antagonist)	10mg	EMD Millipore
# 566660	SAG - Smoothened Agonist	1MG	EMD-Millipore
T-8691<br>T-0625	Taurine		Sigma

Table S1. Oligonucleotides for PCR detection of human retinal genes.

Gene name	Gene ID	Name	Role	Sequence	amplicon size (bp)
SIX6	4990	SIX6guideF	Guide RNA cloning	TTTCTTGGCTTTATATATCTTGTGGAAAGGACGAAA CACC GACATCTGAGTTGCCCATCC	N/A
SIX6	4990	SIX6guideR	Guide RNA cloning	GACTAGCCTTATTTTAACTTGCTATTTCTAGCTCTAA AAC GGATGGGCAACTCAGATGT C	N/A
POU4F2	5458	Brn3bgRNAswap_T2_rev	Guide RNA cloning	AGAGTCTTCTAAATGCCGGCGGTGTTTCGTCCTTTC CACA	N/A
POU4F2	5458	Brn3bgRNAswap_T2F	Guide RNA cloning	GCCGGCATTTAGAAGACTCTGTTTTAGAGCTAGAA ATAGCAA	N/A
POU4F2	5458	Brn3b_insert_rev	Homology arm cloning	CGCGCAAAGCTTGGTACCAAACTGAGTGTCGACTC ACAAATCAGAACAC	1,740
POU4F2	5458	Brn3b_insert_F	Homology arm cloning	CGATGTACGGGCCAGATATACGCCACCTACCACAC TATGAATACCA	-
SIX6	4990	hSIX6GenoT2763 F'set2'	1st pass genotyping	GCAACCGGACTGACCCCTAC	1,801
SIX6	4990	hSIX6GenoT_int R'set2'	1st pass genotyping	AGCTAGTGTACTTGGTAACTGCCTTAGTGC	-
SIX6	4990	hSIX6_GenoT_2763_F 'set1'	2nd pass genotyping	GCA ACC GGA CTG ACC CCT AC	3836 with insert / 2,763 without
SIX6	4990	hSIX6_GenoT_2763_R 'set1'	2nd pass genotyping	TCG AGG CCA TTA CCA GGA CA	-
SIX6	4990	SIX6Geno_het2 (1555)F	genotyping	ACTCCAGCAGCAGGTCCTGTCA	2,728 with insert / 1,555 without
SIX6	4990	SIX6Geno_het2(1555)R	genotyping	CCAGGACAATGAGGCCCTTGA	-
POU4F2	5458	Brn3b_2446_F	1st pass genotyping	CCTGCTGAGCGTAATGTGTGCCTTCTACTT	1,087
POU4F2	5458	Tomato_GT_rev	1st pass genotyping	GCGCATGAACTCTTTGATGACCTCCT	-
POU4F2	5458	Brn3b_2426_F	2nd pass genotyping	CCTGCTGAGCGTAATGTGTGCCTTCTACTT	3320 with insert / 1274 without
POU4F2	5458	Brn3b_2426_R	2nd pass genotyping	CAAACAGCCATCTCCACACTTCCTCTGAA	-
RPL27	6155	hRPL27_123F	qPCR	ATCGCCAAGAGATCAAAGATAA	123
RPL27	6155	hRPL27_123R	qPCR	TCTGAAGACATCCTTATTGACG	-
RPL30	6156	hRPL30_158F	qPCR	ACAGCATGCGGAAAATACTAC	158
RPL30	6156	RPL30_158R	qPCR	AAAGGAAAATTTTGCAGGTT	-
SIX6	4990	hSIX6 198F	qPCR	ACCCCTACGCAGGTGGGCAA	198
SIX6	4990	hSIX6 198R	qPCR	TGAAGTGGCCGCCTTGCTGG	-
SIX6	N/A	H2Bseq150F	qPCR	ACTAAGGCGCAGAAGAAGGA	Insert Sequencing
SIX6	N/A	H2Bseq150R	qPCR	GTCGTTGACGAAGGAGTTCA	Insert Sequencing

Table S2. Off-target site analysis.

Gene name	Off-target score	# Off-target Sites:	Target Sequence	Off-target Sequence	position
SIX6	97.85	3 (0 in CDS)	GACATCTGAGTTGCCCATCC		
	8.69%			AACCTCCGAGTTGCCCATCCTGG	chr1 (Homo sapiens - NC_000001) 181,213,353 -> 181,213,375
	1.71%			TACATCTGGGTTGCCAATCCAGG	chrX (Homo sapiens - NC_000023) 10,613,890 <- 10,613,912
	0.58%			GACTTCTGAGTTGGACATCCAGG	chr15 (Homo sapiens - NC_000015) 87,452,170 -> 87,452,192

TableS3. Upregulated Genes at Day 35 - Cellular Development.

Category	Function	Function Annotation	p-value	Molecules	# Molecules
Cellular Development	development	Development of neurons	4.37E-10	ANGPT1, APBA1, APOE, BLOC1S6, BMP7, CADPS2, CNTN4, CXCL12, EFNA5, EGFR, FEZF2, FLRT2, FLRT3, GFRA1, GJA1, GRN, HES1, LAMC1, LYN, MCF2, PTN, RELN, S1PR3, SEMA3D, SEMA3E, SGK1, SIX3, SLIT2, SMAD3, SOD2, SPARCL1, THBS1, THY1, TIAM1, UGT8, UTRN, VAX2	37
Cellular Development	morphogenesis	Morphogenesis of neurons	1.67E-08	ANGPT1, APBA1, APOE, BLOC1S6, BMP7, CNTN4, CXCL12, EFNA5, EGFR, FEZF2, FLRT3, GFRA1, GJA1, GRN, HES1, LAMC1, LYN, MCF2, PTN, RELN, S1PR3, SEMA3D, SEMA3E, SGK1, SLIT2, THY1, TIAM1, UGT8, VAX2	29
Cellular Development	differentiation	Differentiation of nervous system	4.01E-08	ANGPT1, BMP7, CDON, CNTN4, CXCL12, EFNA5, EGFR, ETV5, FEZF2, GFRA1, HES1, ITM2C, LAMC1, LHX2, LIN28A, PTN, RELN, RORB, S1PR3, SFRP2, SIX3, TCTN1, UGT8, VAX1, VSX2	25
Cellular Development	neuritogenesis	Neuritogenesis	4.85E-08	ANGPT1, APBA1, APOE, BLOC1S6, BMP7, CNTN4, CXCL12, EFNA5, EGFR, FEZF2, FLRT3, GFRA1, GJA1, GRN, LAMC1, LYN, MCF2, PTN, RELN, S1PR3, SEMA3D, SEMA3E, SGK1, SLIT2, THY1, TIAM1, UGT8, VAX2	28
Cellular Development	differentiation	Differentiation of neurons	5.99E-08	BMP7, CDON, CNTN4, CXCL12, EFNA5, EGFR, ETV5, FEZF2, GFRA1, HES1, ITM2C, LAMC1, LHX2, LIN28A, PTN, RORB, S1PR3, SFRP2, SIX3, TCTN1, VAX1, VSX2	22
Cellular Development	axonogenesis	Axonogenesis	0.0000073	ANGPT1, APOE, CNTN4, CXCL12, EFNA5, LAMC1, RELN, SEMA3D, SEMA3E, SLIT2, THY1, TIAM1, VAX2	13
Cellular Development	proliferation	Proliferation of neuronal cells	0.00000806	ANGPT1, APOE, BMP7, CD9, CFLAR, CXCL12, EGFR, ETV5, FRK, GFRA1, GJA1, GRN, MYH9, PTN, RELN, SEMA3D, SEMA5A, SGK1, SLIT2, THBS1, TIAM1, VAX1	22
Cellular Development	branching	Branching of axons	0.0000359	APOE, CXCL12, EFNA5, RELN, SEMA3D, SEMA3E, SLIT2	7
Cellular Development	differentiation	Differentiation of stem cells	0.0000363	ANGPT1, BMP7, ERVW-1, FBN1, FEZF2, GFRA1, HES1, LIN28A, LYN, S1PR3, SFRP2, SMAD3, THBS1	13
Cellular Development	assembly	Assembly of cells	0.0000489	ANGPT1, ANGPTL1, APOE, CD9, CDH13, CXCL12, EGFR, GJA1, GRN, PTN, RGS5, SEMA5A, SMAD3, SPARC, THBS1, THY1	16
Cellular Development	formation	Formation of retinal cells	0.0000992	CRB1, DIO3, RORB, SIX3, THY1, VSX2	6
Cellular Development	differentiation	Differentiation of eye cells	0.000342	HES1, RORB, SIX3, SMAD3, VSX2	5
Cellular Development	development	Development of retinal cone cells	0.000397	DIO3, RORB, THY1	3
Cellular Development	differentiation	Differentiation of neuroglia	0.000419	BMP7, EGFR, HES1, LAMC1, LIN28A, PTN, RELN, S1PR3, UGT8	9

TableS4. Up Regulated Genes at Day 35 - Disease and Function.

.Category	Function	Function Annotation	p-value	Molecules	# Molecules
Developmental Disorder	congenital anomaly of eye	Congenital anomaly of eye	2.12E-11	ALDH1A1, APOE, BLOC1S6, BMP7, FBN1, GPR143, HMX1, LHX2, MAB21L1, MAB21L2, MITF, PXDN, RAX, STRA6, THBS1, VAX1, VAX2, VSX2, WDPCP	19
Visual System Development and Function	morphology	Morphology of eye	2.13E-11	ALDH1A1, APOE, COL2A1, CRB1, CXCL12, DIO3, EFEMP1, GPR143, GRN, HES1, LAMC1, LHX2, MAB21L1, MAB21L2, MITF, SEMA5A, SIX3, SOD2, SPARC, STRA6, THBS1, VAX2, VSX2	23
Developmental Disorder	recessive syndromic microphthalmia	Recessive syndromic microphthalmia	9E-11	HMX1, MAB21L2, MITF, STRA6, VAX1	5
Ophthalmic Disease	anophthalmia or microphthalmia	Anophthalmia or microphthalmia	1.67E-10	BMP7, HMX1, LHX2, MAB21L1, MAB21L2, MITF, RAX, STRA6, THBS1, VAX1, VAX2, VSX2, WDPCP	13
Nervous System Development and Function	abnormal morphology	Abnormal morphology of eye	2.88E-10	ALDH1A1, APOE, COL2A1, CRB1, CXCL12, DIO3, EFEMP1, GPR143, GRN, HES1, LAMC1, LHX2, MAB21L1, MAB21L2, SEMA5A, SOD2, SPARC, STRA6, THBS1, VAX2	20

TableS5. Upregulated Genes - Embryonic Development.

Category	Function	Function Annotation	p-value	Molecules	# Molecules
Embryonic Development	formation	Formation of eye	1.64E-14	ALDH1A1, APOE, BMP7, COL2A1, CPAMD8, CRB1, CXCL12, DIO3, EFEMP1, EGFR, GPR143, GRN, HES1, LAMC1, LHX2, MAB21L1, MAB21L2, MITF, RAX, RORB, SEMA3E, SEMA5A, SIX3, SLIT2, SOD2, SPARC, STRA6, THBS1, THY1, VAX1, VAX2, VSX2	32
Embryonic Development	development	Development of sensory organ	2.46E-14	ALDH1A1, APOE, ATP6V1B1, BMP7, COL2A1, CPAMD8, CRB1, CXCL12, DIO3, EFEMP1, EGFR, GPR143, GRN, HES1, LAMC1, LHX2, MAB21L1, MAB21L2, MITF, PCSK5, PTPRQ, RAX,	36

				RORB, SEMA3E, SEMA5A, SIX3, SLIT2, SOD2, SPARC, STRA6, THBS1, THY1, VAX1, VAX2, VSX2, WDPCP	
Embryonic Development	development	Development of body axis	1.04E-12	ALDH1A1, APLP2, APOE, ATP6V1B1, BMP7, CDON, CFLAR, COL2A1, CPAMD8, CRB1, CXCL12, DIO3, EFEMP1, EFNA5, EGFR, FEZF2, FLRT3, GABRP, GJA1, GPR143, GRN, HES1, LAMC1, LHX2, LIN28A, MAB21L1, MAB21L2, MITF, PCSK5, PKDCC, PTPRQ, RAX, RELN, RORB, SEMA3E, SEMA5A, SFRP2, SIX3, SLIT2, SOD2, SPARC, STRA6, TCTN1, THBS1, THY1, VAX1, VAX2, VSX2, WDPCP	49
Embryonic Development	development	Development of head	1.28E-12	ALDH1A1, APLP2, APOE, ATP6V1B1, BMP7, CDON, COL2A1, CPAMD8, CRB1, CXCL12, DIO3, EFEMP1, EFNA5, EGFR, FEZF2, FLRT3, GABRP, GJA1, GPR143, GRN, HES1, LAMC1, LHX2, LIN28A, MAB21L1, MAB21L2, MITF, PCSK5, PKDCC, PTPRQ, RAX, RELN, RORB, SEMA3E, SEMA5A, SIX3, SLIT2, SOD2, SPARC, STRA6, TCTN1, THBS1, THY1, VAX1, VAX2, VSX2, WDPCP	47
Embryonic Development	formation	Formation of forebrain	0.000000069	APLP2, BMP7, COL2A1, EFNA5, FEZF2, HES1, LHX2, LIN28A, RAX, RELN, SEMA5A, SIX3, SLIT2, TCTN1, VAX1, VAX2	16
Embryonic Development	morphogenesis	Morphogenesis of eye	0.000000244	ALDH1A1, BMP7, MAB21L1, MAB21L2, MITF, SIX3, VAX1, VAX2, VSX2	9
Embryonic Development	formation	Formation of brain	0.000000249	APLP2, APOE, BMP7, CDON, COL2A1, CXCL12, DIO3, EFNA5, EGFR, FEZF2, HES1, LAMC1, LHX2, LIN28A, RAX, RELN, RORB, SEMA5A, SIX3, SLIT2, TCTN1, THBS1, VAX1, VAX2	24
Embryonic Development	development	Development of retina	0.000000256	CRB1, DIO3, LHX2, MITF, RORB, SEMA5A, SIX3, THBS1, THY1, VSX2	10
Embryonic Development	development	Development of body trunk	0.00000831	ALDH1A1, ANGPT1, APOE, BMP7, CD9, CFLAR, COL2A1, CXCL12, DUSP6, EGFR, EMP1, ETV5, FBN1, FGF19, FLRT2, FLRT3, FSTL1, GJA1, HAS2, HES1, IGF2R, KRT8, LAMC1, PCSK5, PKDCC, PTN, S1PR3, SLIT2, SMAD3, SOD2, SPARC, STRA6, THBS1, TPM1, UTRN	35
Embryonic Development	patterning	Patterning of eye	0.00000938	SIX3, VAX2, VSX2	3
Embryonic Development	branching	Branching of axons	0.0000359	APOE, CXCL12, EFNA5, RELN, SEMA3D, SEMA3E, SLIT2	7
Embryonic Development	formation	Formation of retinal cells	0.0000992	CRB1, DIO3, RORB, SIX3, THY1, VSX2	6
Embryonic Development	patterning	Patterning of retina	0.00029	VAX2, VSX2	2
Embryonic Development	development	Development of retinal cone cells	0.000397	DIO3, RORB, THY1	3
Embryonic Development	development	Development of diencephalon	0.000427	RAX, SEMA5A, SIX3, SLIT2	4
Embryonic Development	formation	Formation of telencephalon	0.000524	EFNA5, FEZF2, HES1, LHX2, SIX3, SLIT2, TCTN1, VAX1	8

TableS6. Upregulated Genes at Day 35 - Eye related.

Category	Function	Function Annotation	p-value	Molecules	# Molecules
Visual System Development and Function	formation	Formation of eye	1.64E-14	ALDH1A1, APOE, BMP7, COL2A1, CPAMD8, CRB1, CXCL12, DIO3, EFEMP1, EGFR, GPR143, GRN, HES1, LAMC1, LHX2, MAB21L1, MAB21L2, MITF, RAX, RORB, SEMA3E, SEMA5A, SIX3, SLIT2, SOD2, SPARC, STRA6, THBS1, THY1, VAX1, VAX2, VSX2	32
Visual System Development and Function	morphology	Morphology of eye	2.13E-11	ALDH1A1, APOE, COL2A1, CRB1, CXCL12, DIO3, EFEMP1, GPR143, GRN, HES1, LAMC1, LHX2, MAB21L1, MAB21L2, MITF, SEMA5A, SIX3, SOD2, SPARC, STRA6, THBS1, VAX2, VSX2	23
Visual System Development and Function	morphology	Morphology of retina	6.84E-11	APOE, CRB1, CXCL12, DIO3, GPR143, GRN, HES1, LAMC1, LHX2, MAB21L1, MAB21L2, MITF, SEMA5A, SIX3, SOD2, STRA6, VAX2, VSX2	18
Ophthalmic Disease	recessive syndromic microphthalmia	Recessive syndromic microphthalmia	9E-11	HMX1, MAB21L2, MITF, STRA6, VAX1	5
Ophthalmic Disease	anophthalmia or microphthalmia	Anophthalmia or microphthalmia	1.67E-10	BMP7, HMX1, LHX2, MAB21L1, MAB21L2, MITF, RAX, STRA6, THBS1, VAX1, VAX2, VSX2, WDPCP	13
Visual System Development and Function	abnormal morphology	Abnormal morphology of eye	2.88E-10	ALDH1A1, APOE, COL2A1, CRB1, CXCL12, DIO3, EFEMP1, GPR143, GRN, HES1, LAMC1, LHX2, MAB21L1, MAB21L2, SEMA5A, SOD2, SPARC, STRA6, THBS1, VAX2	20
Ophthalmic Disease	microphthalmia	Microphthalmia	4.96E-09	BMP7, HMX1, MAB21L1, MAB21L2, MITF, RAX, STRA6, THBS1, VAX1, VAX2, VSX2	11
Visual System Development and Function	abnormal morphology	Abnormal morphology of retina	8.49E-09	APOE, CRB1, CXCL12, DIO3, GPR143, GRN, HES1, LAMC1, LHX2, MAB21L1, MAB21L2, SEMA5A, SOD2, STRA6, VAX2	15
Ophthalmic Disease	abnormality	Abnormality of choroid	6.53E-08	ANGPT1, APOE, BMP7, GPR143, MAB21L1, MAB21L2, SOD2, STRA6	8

Visual System Development and Function	morphogenesis	Morphogenesis of camera-type eye	0.000000115	BMP7, MAB21L1, MAB21L2, MITF, SIX3, VAX1, VAX2, VSX2	8
Visual System Development and Function	abnormal morphology	Abnormal morphology of retinal layer	0.000000156	APOE, GPR143, GRN, HES1, MAB21L1, MAB21L2, SEMA5A, SOD2, STRA6, VAX2	10
Visual System Development and Function	morphogenesis	Morphogenesis of eye	0.000000244	ALDH1A1, BMP7, MAB21L1, MAB21L2, MITF, SIX3, VAX1, VAX2, VSX2	9
Visual System Development and Function	development	Development of retina	0.000000256	CRB1, DIO3, LHX2, MITF, RORB, SEMA5A, SIX3, THBS1, THY1, VSX2	10
Ophthalmic Disease	abnormal morphology	Abnormal morphology of neurosensory retina	0.00000101	GPR143, GRN, HES1, MAB21L1, MAB21L2, SEMA5A, SOD2, STRA6, VAX2	9
Ophthalmic Disease	hereditary eye disease	Hereditary Eye Disease	0.00000105	APOE, BLOC1S6, CA12, COL2A1, COL9A1, CPAMD8, CRB1, EFEMP1, FBN1, FBN2, GPR143, HMX1, MAB21L2, MITF, PXDN, RAX, STRA6, VAX1, VSX2	19
Visual System Development and Function	morphology	Morphology of eye cells	0.00000186	CRB1, CXCL12, DIO3, MAB21L2, MITF, SEMA5A, SIX3, SPARC, STRA6, VSX2	10
Ophthalmic Disease	coloboma	Coloboma	0.00000216	MAB21L2, MITF, STRA6, VAX1, VAX2, VSX2	6
Visual System Development and Function	morphology	Morphology of retinal cells	0.00000432	CRB1, CXCL12, DIO3, MAB21L2, MITF, SEMA5A, SIX3, STRA6, VSX2	9
Hereditary Disorder	autosomal recessive congenital eye anomaly	Autosomal recessive congenital eye anomaly	0.00000486	APOE, BLOC1S6, HMX1, MAB21L2, MITF, STRA6, VAX1	7
Visual System Development and Function	morphology	Morphology of retinal pigment epithelium	0.00000755	GPR143, MAB21L1, MAB21L2, MITF, SOD2, STRA6	6
Ophthalmic Disease	aphakia	Aphakia	0.0000116	BMP7, LHX2, MAB21L1, MAB21L2	4
Ophthalmic Disease	autosomal recessive ocular coloboma	Autosomal recessive ocular coloboma	0.0000324	MAB21L2, MITF, STRA6	3
Visual System Development and Function	abnormal morphology	Abnormal morphology of retinal pigment epithelium	0.0000505	GPR143, MAB21L1, MAB21L2, SOD2, STRA6	5
Visual System Development and Function	abnormal morphology	Abnormal morphology of thin inner nuclear layer	0.0000765	APOE, MAB21L1, SOD2	3
Visual System Development and Function	formation	Formation of retinal cells	0.0000992	CRB1, DIO3, RORB, SIX3, THY1, VSX2	6
Visual System Development and Function	abnormal morphology	Abnormal morphology of inner nuclear layer	0.000237	APOE, MAB21L1, SEMA5A, SOD2	4
Hereditary Disorder	isolated microphthalmia	Isolated microphthalmia	0.000253	RAX, STRA6, VSX2	3
Ophthalmic Disease	abnormality	Abnormality of uveal tract	0.000341	ALDH1A1, COL2A1, GPR143, MAB21L1	4
Visual System Development and Function	development	Development of retinal cone cells	0.000397	DIO3, RORB, THY1	3
Ophthalmic Disease	anophthalmia	Anophthalmia	0.000526	BMP7, LHX2, RAX, WDPCP	4

TableS7. Upregulated Genes - Nervous System Development and Function at Day 35.

Category	Function	Function Annotation	p-value	Molecules	# Molecules
Nervous System	formation	Formation of eye	1.64E-14	ALDH1A1, APOE, BMP7, COL2A1, CPAMD8, CRB1, CXCL12, DIO3, EFEMP1, EGFR, GPR143, GRN, HES1, LAMC1, LHX2,	32

Development and Function				MAB21L1, MAB21L2, MITF, RAX, RORB, SEMA3E, SEMA5A, SIX3, SLIT2, SOD2, SPARC, STRA6, THBS1, THY1, VAX1, VAX2, VSX2	
Nervous System Development and Function	development	Development of neurons	4.37E-10	ANGPT1, APBA1, APOE, BLOC1S6, BMP7, CADPS2, CNTN4, CXCL12, EFNA5, EGFR, FEZF2, FLRT2, FLRT3, GFRA1, GJA1, GRN, HES1, LAMC1, LYN, MCF2, PTN, RELN, S1PR3, SEMA3D, SEMA3E, SGK1, SIX3, SLIT2, SMAD3, SOD2, SPARCL1, THBS1, THY1, TIAM1, UGT8, UTRN, VAX2	37
Nervous System Development and Function	guidance	Guidance of axons	2.81E-08	BMP7, CNTN4, CXCL12, EFNA5, FEZF2, FLRT2, FLRT3, LHX2, RELN, SEMA5A, SLIT2, VAX1, VAX2	13
Nervous System Development and Function	neuritogenesis	Neuritogenesis	4.85E-08	ANGPT1, APBA1, APOE, BLOC1S6, BMP7, CNTN4, CXCL12, EFNA5, EGFR, FEZF2, FLRT3, GFRA1, GJA1, GRN, LAMC1, LYN, MCF2, PTN, RELN, S1PR3, SEMA3D, SEMA3E, SGK1, SLIT2, THY1, TIAM1, UGT8, VAX2	28
Nervous System Development and Function	development	Development of central nervous system	5.08E-08	ALDH5A1, ANGPT1, APLP2, APOE, BMP7, CDON, COL2A1, CXCL12, DIO3, EFNA5, EGFR, FEZF2, GSTP1, HES1, LAMC1, LHX2, LIN28A, PTN, RAX, RELN, RORB, SEMA5A, SIX3, SLIT2, TCTN1, THBS1, UGT8, VAX1, VAX2	29
Nervous System Development and Function	differentiation	Differentiation of neurons	5.99E-08	BMP7, CDON, CNTN4, CXCL12, EFNA5, EGFR, ETV5, FEZF2, GFRA1, HES1, ITM2C, LAMC1, LHX2, LIN28A, PTN, RORB, S1PR3, SFRP2, SIX3, TCTN1, VAX1, VSX2	22
Nervous System Development and Function	formation	Formation of forebrain	6.9E-08	APLP2, BMP7, COL2A1, EFNA5, FEZF2, HES1, LHX2, LIN28A, RAX, RELN, SEMA5A, SIX3, SLIT2, TCTN1, VAX1, VAX2	16
Nervous System Development and Function	morphogenesis	Morphogenesis of eye	2.44E-07	ALDH1A1, BMP7, MAB21L1, MAB21L2, MITF, SIX3, VAX1, VAX2, VSX2	9
Nervous System Development and Function	formation	Formation of brain	2.49E-07	APLP2, APOE, BMP7, CDON, COL2A1, CXCL12, DIO3, EFNA5, EGFR, FEZF2, HES1, LAMC1, LHX2, LIN28A, RAX, RELN, RORB, SEMA5A, SIX3, SLIT2, TCTN1, THBS1, VAX1, VAX2	24
Nervous System Development and Function	development	Development of retina	2.56E-07	CRB1, DIO3, LHX2, MITF, RORB, SEMA5A, SIX3, THBS1, THY1, VSX2	10
Nervous System Development and Function	growth	Growth of neurites	1.96E-06	ANGPT1, APOE, BMP7, CD9, CFLAR, CXCL12, EGFR, ETV5, FRK, GFRA1, GJA1, GRN, MYH9, PTN, RELN, SEMA3D, SEMA5A, SGK1, SLIT2, THBS1, TIAM1	21
Nervous System Development and Function	axonogenesis	Axonogenesis	7.3E-06	ANGPT1, APOE, CNTN4, CXCL12, EFNA5, LAMC1, RELN, SEMA3D, SEMA3E, SLIT2, THY1, TIAM1, VAX2	13
Nervous System Development and Function	proliferation	Proliferation of neuronal cells	8.06E-06	ANGPT1, APOE, BMP7, CD9, CFLAR, CXCL12, EGFR, ETV5, FRK, GFRA1, GJA1, GRN, MYH9, PTN, RELN, SEMA3D, SEMA5A, SGK1, SLIT2, THBS1, TIAM1, VAX1	22
Nervous System Development and Function	migration	Migration of neurons	1.34E-05	CADPS2, CXCL12, EFNA5, FLRT2, GJA1, LAMC1, MYH9, PTN, RELN, SIX3, SLIT2, TIAM1, VAX1	13
Nervous System Development and Function	innervation	Innervation of optic nerve	1.86E-05	GPR143, VAX1, VAX2	3
Nervous System Development and Function	branching	Branching of axons	3.59E-05	APOE, CXCL12, EFNA5, RELN, SEMA3D, SEMA3E, SLIT2	7
Nervous System Development and Function	extension	Extension of neurites	4.79E-05	APOE, CXCL12, LHX2, MYH9, RELN, RSU1, SEMA3E, SEMA5A, SLIT2, TIAM1	10
Nervous System Development and Function	formation	Formation of retinal cells	9.92E-05	CRB1, DIO3, RORB, SIX3, THY1, VSX2	6
Nervous System Development and Function	contact repulsion	Contact repulsion of axons	0.000109	SEMA3D, SEMA3E, SLIT2	3
Nervous System Development and Function	extension	Extension of axons	0.000261	APOE, CXCL12, LHX2, RELN, SEMA3E, SEMA5A, SLIT2	7

Nervous System Development and Function	innervation	Innervation	0.000335	CDH6, EFNA5, EGFR, GFRA1, GPR143, SLIT2, VAX1, VAX2	8
Nervous System Development and Function	fasciculation	Fasciculation of axons	0.000341	CNTN4, FEZF2, SEMA5A, SLIT2	4
Nervous System Development and Function	development	Development of retinal cone cells	0.000397	DIO3, RORB, THY1	3
Nervous System Development and Function	differentiation	Differentiation of neuroglia	0.000419	BMP7, EGFR, HES1, LAMC1, LIN28A, PTN, RELN, S1PR3, UGT8	9
Nervous System Development and Function	development	Development of diencephalon	0.000427	RAX, SEMA5A, SIX3, SLIT2	4
Nervous System Development and Function	formation	Formation of telencephalon	0.000524	EFNA5, FEZF2, HES1, LHX2, SIX3, SLIT2, TCTN1, VAX1	8

TableS8. Upregulated Genes - Tissue and Organ Development at Day35.

Category	Function	Function Annotation	p-value	Molecules	# Molecules
Organ Development	formation	Formation of eye	1.64E-14	ALDH1A1, APOE, BMP7, COL2A1, CPAMD8, CRB1, CXCL12, DIO3, EFEMP1, EGFR, GPR143, GRN, HES1, LAMC1, LHX2, MAB21L1, MAB21L2, MITF, RAX, RORB, SEMA3E, SEMA5A, SIX3, SLIT2, SOD2, SPARC, STRA6, THBS1, THY1, VAX1, VAX2, VSX2	32
Organismal Development	development	Development of body axis	1.04E-12	ALDH1A1, APLP2, APOE, ATP6V1B1, BMP7, CDON, CFLAR, COL2A1, CPAMD8, CRB1, CXCL12, DIO3, EFEMP1, EFNA5, EGFR, FEZF2, FLRT3, GABRP, GJA1, GPR143, GRN, HES1, LAMC1, LHX2, LIN28A, MAB21L1, MAB21L2, MITF, PCSK5, PKDCC, PTPRQ, RAX, RELN, RORB, SEMA3E, SEMA5A, SFRP2, SIX3, SLIT2, SOD2, SPARC, STRA6, TCTN1, THBS1, THY1, VAX1, VAX2, VSX2, WDPCP	49
Organismal Development	development	Development of head	1.28E-12	ALDH1A1, APLP2, APOE, ATP6V1B1, BMP7, CDON, COL2A1, CPAMD8, CRB1, CXCL12, DIO3, EFEMP1, EFNA5, EGFR, FEZF2, FLRT3, GABRP, GJA1, GPR143, GRN, HES1, LAMC1, LHX2, LIN28A, MAB21L1, MAB21L2, MITF, PCSK5, PKDCC, PTPRQ, RAX, RELN, RORB, SEMA3E, SEMA5A, SIX3, SLIT2, SOD2, SPARC, STRA6, TCTN1, THBS1, THY1, VAX1, VAX2, VSX2, WDPCP	47
Tissue Development	development	Development of neurons	4.37E-10	ANGPT1, APBA1, APOE, BLOC1S6, BMP7, CADPS2, CNTN4, CXCL12, EFNA5, EGFR, FEZF2, FLRT2, FLRT3, GFRA1, GJA1, GRN, HES1, LAMC1, LYN, MCF2, PTN, RELN, S1PR3, SEMA3D, SEMA3E, SGK1, SIX3, SLIT2, SMAD3, SOD2, SPARCL1, THBS1, THY1, TIAM1, UGT8, UTRN, VAX2	37
Organismal Development	morphogenesis	Morphogenesis of embryo	1.25E-08	ADAMTS16, BMP7, CDON, COL2A1, EGFR, FBN2, FLRT3, GJA1, HES1, LGR4, MAB21L2, PCSK5, PKDCC, SFRP2, SMAD3, THBS1, WDPCP	17
Organismal Development	morphogenesis	Morphogenesis of neurons	1.67E-08	ANGPT1, APBA1, APOE, BLOC1S6, BMP7, CNTN4, CXCL12, EFNA5, EGFR, FEZF2, FLRT3, GFRA1, GJA1, GRN, HES1, LAMC1, LYN, MCF2, PTN, RELN, S1PR3, SEMA3D, SEMA3E, SGK1, SLIT2, THY1, TIAM1, UGT8, VAX2	29
Organismal Development	neuritogenesis	Neuritogenesis	4.85E-08	ANGPT1, APBA1, APOE, BLOC1S6, BMP7, CNTN4, CXCL12, EFNA5, EGFR, FEZF2, FLRT3, GFRA1, GJA1, GRN, LAMC1, LYN, MCF2, PTN, RELN, S1PR3, SEMA3D, SEMA3E, SGK1, SLIT2, THY1, TIAM1, UGT8, VAX2	28
Tissue Development	differentiation	Differentiation of neurons	5.99E-08	BMP7, CDON, CNTN4, CXCL12, EFNA5, EGFR, ETV5, FEZF2, GFRA1, HES1, ITM2C, LAMC1, LHX2, LIN28A, PTN, RORB, S1PR3, SFRP2, SIX3, TCTN1, VAX1, VSX2	22
Organ Development	formation	Formation of forebrain	0.00000006 9	APLP2, BMP7, COL2A1, EFNA5, FEZF2, HES1, LHX2, LIN28A, RAX, RELN, SEMA5A, SIX3, SLIT2, TCTN1, VAX1, VAX2	16
Organ Development	morphogenesis	Morphogenesis of eye	0.00000024 4	ALDH1A1, BMP7, MAB21L1, MAB21L2, MITF, SIX3, VAX1, VAX2, VSX2	9
Organ Development	formation	Formation of brain	0.00000024 9	APLP2, APOE, BMP7, CDON, COL2A1, CXCL12, DIO3, EFNA5, EGFR, FEZF2, HES1, LAMC1, LHX2, LIN28A, RAX, RELN, RORB, SEMA5A, SIX3, SLIT2, TCTN1, THBS1, VAX1, VAX2	24
Organ Development	development	Development of retina	0.00000025 6	CRB1, DIO3, LHX2, MITF, RORB, SEMA5A, SIX3, THBS1, THY1, VSX2	10
Organismal Development	morphogenesis	Morphogenesis of head	0.00000084 5	ALDH1A1, ATP6V1B1, BMP7, COL2A1, EGFR, HES1, MAB21L1, MAB21L2, MITF, PTPRQ, SIX3, STRA6, VAX1, VAX2, VSX2	15

Tissue Development	growth	Growth of neurites	0.00000196	ANGPT1, APOE, BMP7, CD9, CFLAR, CXCL12, EGFR, ETV5, FRK, GFRA1, GJA1, GRN, MYH9, PTN, RELN, SEMA3D, SEMA5A, SGK1, SLIT2, THBS1, TIAM1	21
Tissue Development	axonogenesis	Axonogenesis	0.0000073	ANGPT1, APOE, CNTN4, CXCL12, EFNA5, LAMC1, RELN, SEMA3D, SEMA3E, SLIT2, THY1, TIAM1, VAX2	13
Organ Development	formation	Formation of retinal cells	0.0000992	CRB1, DIO3, RORB, SIX3, THY1, VSX2	6

TableS9. Downregulated Genes - Cellular Development at Day35.

Category	Function	Function Annotation	p-value	Molecules	# Molecules
Cellular Development	development	Development of neurons	9.07E-34	ACVR2A, ADGRL2, ADGRL3, APC, ARHGEF7, ARX, ASCL1, BASP1, BRSK2, BTBD3, CBLN1, CDH2, CDK5R1, CDK6, CELSR2, CHL1, CHN1, CKB, CLASP2, CNR1, CNTN1, CNTN2, CNTNAP2, CPEB4, CRMP1, CTNNA2, CUX2, CXCR4, DCC, DCLK1, DCX, DLX1, DPYSL2, DPYSL3, DPYSL5, EBF3, ECEL1, EFNB1, EFNB2, ELAVL4, ENC1, EPHA3, EPHA7, ERBB4, FGFR2, FGFR3, FNBP1L, FYN, GABRA5, GABRB3, GAP43, GDI1, GNAO1, GPM6A, GPRIN1, HDAC2, HES5, HEY1, HMGB1, HMGB2, HNRNPK, HOXA1, HS6ST1, ID1, IRX3, KIDINS220, KIF3A, L1CAM, LINGO1, LOX, LRRN3, LYPLA1, MAP1B, MAP2, MAP6, MAPK8, MAPT, MYCN, NCAM1, NCAN, NEFM, NEUROD1, NEUROD4, NEUROG1, NEUROG2, NFIA, NFIB, NIN, NOTCH1, NRXN1, NSG1, NTRK2, OTX2, PAK3, PAX6, PBX3, PDZRN3, PFN2, POU3F2, PPP2CA, PROX1, PTPRZ1, RAB10, RAB3A, RAPGEF2, RBPJ, RERE, RGS2, RND2, RNF165, ROBO2, ROBO3, RTN3, RTN4, RUFY3, SEZ6L, SKIL, SLC12A2, SLC1A3, SLC9A6, SOX11, SOX2, SOX4, SPTBN2, STAU2, STMN1, TOP2B, TPBG, TRIM9, UBE2V2, UBQLN2, VEGFA, WASF1, WNT3, YWHAG, ZBTB18, ZEB2, ZSWIM6	138
Cellular Development	outgrowth	Outgrowth of neurites	4.11E-15	APBA2, ARHGEF7, ARX, BASP1, BRSK2, CDH2, CDK5R1, CHL1, CHN1, CNR1, CSNK1E, DCC, DCX, DOK6, DPYSL2, DPYSL3, DPYSL5, EFNB1, EFNB2, ELAVL4, ERBB4, EXOC5, FEZF1, FGFR3, FZD3, GAP43, GDAP1, GNAO1, GNAQ, GNAS, GPM6A, GPRIN1, HMGB1, HMGCR, KIF3A, KIF3C, KRAS, L1CAM, MAP1B, MAP2, MAPK8, MAPT, MIB1, NCAM1, NFIA, NRXN1, NTRK2, PFN2, PTPRZ1, RND2, RTN4, SET, SKIL, SLC12A2, STMN2, TNFRSF19, TOP1, TUBA1A, VCAM1, VEGFA, YWHAZ, ZBTB18	62
Cellular Development	axonogenesis	Axonogenesis	6.88E-14	APC, ASCL1, BRSK2, CDH2, CHN1, CLASP2, CNTN2, CTNNA2, DCC, DCLK1, DPYSL2, FGFR2, GAP43, GDI1, L1CAM, MAP1B, MAP2, MAP6, MAPT, NFIA, NFIB, NIN, NOTCH1, NTRK2, PAK3, PAX6, POU3F2, PPP2CA, PTPRZ1, RAB10, RAB3A, RNF165, ROBO2, ROBO3, RTN4, RUFY3, SKIL, SLC9A6, STMN1, TOP2B, TRIM9, ZEB2	42
Cellular Development	differentiation	Differentiation of retina	1.32E-10	ASCL1, DLX1, EBF1, EBF3, HES6, KDM6B, NEUROD1, NEUROD4, NKD1, NOTCH1, OTX1, OTX2, PAX6, SOX9	14
Cellular Development	development	Development of neuroglia	2.86E-09	ASCL1, CDK6, DLL1, DLX1, ERBB4, FGFR2, FGFR3, HES5, HEY1, KRAS, NEUROD4, NEUROG2, NOTCH1, POU3F2, RBPJ, SOX11, SOX4, TCF7L2, WASF3	19
Cellular Development	developmental process	Developmental process of synapse	3.64E-09	ADGRL2, ADGRL3, ARHGEF7, CBLN1, CDH2, CNTN1, CUX2, DCC, DCLK1, EPHA3, EPHA7, ERBB4, FGFR2, FYN, GPM6A, HDAC2, LRRN3, MYCN, NCAM1, NCAN, NFIA, NRXN1, NTRK2, PDZRN3, RGS2, RTN4, SEZ6L, SLC12A2, SPTBN2, STAU2, TPBG, UBE2V2, YWHAG	33
Cellular Development	differentiation	Differentiation of brain cells	9.08E-09	ASCL1, CBLN1, CDK5R1, CNR1, HDAC2, HES5, ID1, ID3, MAPT, MIB1, NEUROD1, NEUROG2, NRXN1, PAX6, POU3F2, PROX1, SOX9, WNT4	18
Cellular Development	differentiation	Differentiation of eye cells	0.000000042	ASCL1, DLX1, EBF1, EBF3, HES6, KDM6B, NEUROD1, NEUROD4, NKD1, NOTCH1, OTX1, OTX2, SKIL, SOX9	14
Cellular Development	differentiation	Differentiation of oligodendrocytes	6.08E-08	ASCL1, CNTN1, CXCR4, DLX1, FGFR3, HDAC2, HES5, KHDRBS1, LINGO1, NEUROG1, NOTCH1, NTRK2, OTX2, PTPRZ1, QKI, RTN4, SOX9, TCF7L2, ZBTB18	19
Cellular Development	maturation	Maturation of neurons	7.65E-08	ASCL1, CDKN1C, CNR1, DCLK1, EFNB1, EPHA7, ERBB4, HDAC2, MAPT, NEUROD1, NFIA, NRXN1, RAB3A, SEZ6L, SLC12A2, STXBP1	16
Cellular Development	epithelial-mesenchymal transition	Epithelial-mesenchymal transition	0.000000123	ARHGAP21, CLIC4, EIF3E, EPHA3, FGFR2, FOXA1, FZD7, GPI, HEY1, ID1, IDH1, JAG1, KDM6B, KRAS, LEF1, MSX1, MYCN, NFYB, NOTCH1, NTRK2, PPP2CA, PTPRZ1, RBPJ, RLIM, SKIL, SOX2, SOX9, STMN1, VEGFA, WNT4, ZEB1, ZEB2	32
Cellular Development	differentiation	Differentiation of photoreceptors	0.000000165	ASCL1, DLX1, EBF1, EBF3, HES6, KDM6B, NKD1, NOTCH1, SOX9	9
Cellular Development	formation	Formation of brain cells	0.00000202	ARX, ASCL1, CHL1, CNR1, CXCR4, FGFR2, KIF3A, L1CAM, NCAM1, NEUROD1, NEUROG2, NOTCH1, POU3F2	13
Cellular Development	development	Development of astrocytes	0.00000238	ASCL1, CDK6, DLL1, ERBB4, HEY1, KRAS, NEUROG2, POU3F2, RBPJ	9

Table S10. Down Regulated Genes at Day 35 - Disease and Function.

Category	Function	Function Annotation	p-value	Molecules	# Molecules
Nervous System Development and Function	development	Development of central nervous system	4.12E-35	ARX, ASCL1, ATAT1, BHLHE22, BTBD3, CCND2, CDH2, CDK5R1, CDK6, CELSR2, CHL1, CITED2, CNBP, CNR1, CNTN1, CNTNAP2, CTNNA2, CXADR, CXCR4, DCLK1, DCX, DLL1, DLX1, DRAXIN, EBF3, EGLN1, EPHA7, ERBB4, FEZF1, FGFR2, FGFR3, FUBP1, FYN, FZD3, GAP43, GNAO1, HDAC2, HES5, HEY1, HNRNPK, HOXA1, HOXB1, HOXB2, HOXB8, IPMK, IRX3, KDM6B, KHDRBS1, KIF3A, KRAS, L1CAM, LEF1, LINGO1, MAP1B, MAP2, MAPK8, MAPT, MARCKSL1, MGEA5, MSX1, MYCN, MYT1, NCAM1, NEUROD1, NEUROD4, NEUROG1, NEUROG2, NFIA, NFIB, NHLH1, NHLH2, NIN, NOTCH1, NPAS3, NR2F2, NR3C1, NTRK2, OTX1, OTX2, PAX6, PBX3, PLCB1, POU3F2, POU3F3, PRDM8, PROX1, PTCH1, PTPRZ1, QKI, RAPGEF2, RARB, RBPJ, RERE, RFX4, ROBO2, ROBO3, RPL24, RTN4, S1PR1, SIM1, SLC1A2, SLC1A3, SLC4A7, SMAD9, SOX1, SOX11, SOX2, SOX4, SOX9, STXBP1, TAGLN3, TCF7L2, TDG, TOP2B, VEGFA, WASF3, WLS, YBX1, ZBTB18, ZEB1, ZEB2, ZIC1, ZIC4	123
Cellular Development	differentiation	Differentiation of nervous system	4.98E-31	ACSL4, ADNP2, ARHGEF2, ARX, ASCL1, BHLHE22, BRINP1, BRSK2, C8orf46, CBLN1, CDC25B, CDH2, CDK5R1, CNR1, CNTN1, CNTN2, CXCR4, CYP26B1, DLL1, DLL3, DLX1, DPYSL2, EBF1, EBF3, EFNB1, ELAVL2, ELAVL3, ELAVL4, ERBB4, FEZF1, FGFR2, FGFR3, FOXA1, GAP43, GPC2, HDAC2, HES5, HEY1, HNRNPK, HOXA1, HOXB1, HOXB2, HOXD3, ID1, ID3, INSM1, IRX3, JAG1, KDM6B, KHDRBS1, KIDINS220, LEF1, LINGO1, MAP1B, MAPK8, MAPT, MIB1, MMD, MYCN, NCAM1, NEUROD1, NEUROD4, NEUROG1, NEUROG2, NFIA, NFIB, NOTCH1, NREP, NRXN1, NTRK2, OLIG3, OTX2, PAX6, POU3F2, PROX1, PTPRZ1, QKI, RARB, RBPJ, RTN4, S1PR1, SALL3, SLC1A2, SLC1A3, SOX1, SOX11, SOX2, SOX4, SOX9, SPAG9, TCF12, TCF7L2, TRIM9, VEGFA, WNT3, WNT4, YWHAG, ZBTB18, ZEB1, ZNF536	100
Nervous System Development and Function	formation	Formation of brain	1.62E-29	ARX, ASCL1, ATAT1, BHLHE22, BTBD3, CCND2, CDH2, CDK5R1, CDK6, CELSR2, CHL1, CNBP, CNR1, CNTN1, CNTNAP2, CTNNA2, CXADR, CXCR4, DCLK1, DCX, DLL1, DLX1, DRAXIN, EBF3, EGLN1, EPHA7, ERBB4, FEZF1, FGFR2, FGFR3, FYN, FZD3, GAP43, GNAO1, HDAC2, HES5, HNRNPK, HOXA1, HOXB1, HOXB2, IPMK, IRX3, KDM6B, KHDRBS1, KIF3A, L1CAM, LEF1, MAP1B, MAPK8, MAPT, MARCKSL1, MGEA5, MSX1, MYCN, NCAM1, NEUROD1, NEUROD4, NEUROG2, NFIA, NFIB, NIN, NOTCH1, NPAS3, NR2F2, NTRK2, OTX1, OTX2, PAX6, PLCB1, POU3F2, POU3F3, PRDM8, PROX1, PTCH1, RAPGEF2, RARB, RERE, RFX4, ROBO2, ROBO3, S1PR1, SIM1, SLC1A2, SLC1A3, SLC4A7, SMAD9, SOX1, SOX2, SOX4, STXBP1, TDG, TOP2B, VEGFA, WLS, YBX1, ZBTB18, ZEB2, ZIC1, ZIC4	99
Cellular Movement	migration	Migration of neurons	9.84E-29	ADGRL3, APC, ARX, ASCL1, ATAT1, AUTS2, CDK5R1, CELSR2, CHL1, CNR1, CNTN2, CNTNAP2, CTNNA2, CXCR4, DCC, DCLK1, DCX, DLX1, EBF1, EBF3, EDNRB, EFNB1, EFNB2, FEZF1, FYN, FZD3, GPM6A, HMGB1, HOXA1, HOXB1, HOXB2, L1CAM, MAP1B, MAP2, MAPK8, MAPT, NDNF, NEUROD4, NEUROG1, NEUROG2, NFIA, NHLH2, NR2F2, NTRK2, PAX6, POU3F2, POU3F3, PTPRZ1, RAPGEF2, RERE, ROBO3, RTN4, SKIL, SLC12A2, SLC1A2, SLC1A3, SOX1, STMN1, TOP2B, TUBA1A, TUBB2B, VEGFA, ZBTB18	63
Tissue Development	differentiation	Differentiation of neurons	4.27E-28	ACSL4, ADNP2, ARHGEF2, ARX, ASCL1, BHLHE22, BRINP1, BRSK2, C8orf46, CBLN1, CDK5R1, CNR1, CNTN2, CYP26B1, DLL1, DLX1, DPYSL2, EBF1, EBF3, EFNB1, ELAVL2, ELAVL3, ELAVL4, ERBB4, FEZF1, FGFR2, FGFR3, FOXA1, GAP43, GPC2, HDAC2, HES5, HEY1, HNRNPK, HOXA1, HOXB1, HOXB2, HOXD3, ID3, INSM1, IRX3, JAG1, KDM6B, KIDINS220, LEF1, MAP1B, MAPK8, MAPT, MIB1, MMD, NCAM1, NEUROD1, NEUROD4, NEUROG1, NEUROG2, NOTCH1, NREP, NRXN1, NTRK2, OLIG3, OTX2, PAX6, POU3F2, PROX1, PTPRZ1, RARB, RBPJ, RTN4, S1PR1, SALL3, SLC1A2, SLC1A3, SOX1, SOX11, SOX2, SOX4, SPAG9, TCF12, TRIM9, VEGFA, WNT3, YWHAG, ZBTB18, ZEB1, ZNF536	85
Cell Morphology	morphogenesis	Morphogenesis of neurons	3.3E-23	ACVR2A, APC, ARHGEF7, ASCL1, BASP1, BRSK2, BTBD3, CDH2, CDK5R1, CELSR2, CHL1, CHN1, CKB, CLASP2, CNR1, CNTN1, CNTN2, CNTNAP2, CPEB4, CRMP1, CTNNA2, CUX2, DCC, DCLK1, DCX, DPYSL2, DPYSL3, DPYSL5, ECEL1, EFNB1, EFNB2, ELAVL4, ENC1, EPHA7, ERBB4, FGFR2, FNBP1L, FYN, GABRB3, GAP43, GDI1, GNAO1, GPM6A, GPRIN1, HDAC2, HMGB1, HMGB2, HNRNPK, ID1, KIDINS220, KIF3A, L1CAM, LINGO1, LOX, LYPLA1, MAP1B, MAP2, MAP6, MAPK8, MAPT, NCAM1, NEFM, NEUROD1, NFIA, NFIB, NIN, NOTCH1, NSG1, NTRK2, PAK3, PAX6, PFN2, POU3F2, PPP2CA, PTPRZ1, RAB10, RAB3A, RAPGEF2, RERE, RND2, RNF165, ROBO2, ROBO3, RTN3, RTN4, RUFY3, SKIL, SLC1A3, SLC9A6, SPTBN2, STAU2, STMN1, TOP2B, TPBG, TRIM9, UBQLN2, WASF1, WNT3, ZEB2, ZSWIM6	100
Nervous System Development and Function	neuritogenesis	Neuritogenesis	4.68E-23	ACVR2A, APC, ARHGEF7, ASCL1, BASP1, BRSK2, BTBD3, CDH2, CDK5R1, CELSR2, CHL1, CHN1, CKB, CLASP2, CNR1, CNTN1, CNTN2, CNTNAP2, CPEB4, CRMP1, CTNNA2, CUX2, DCC, DCLK1, DCX, DPYSL2, DPYSL3, DPYSL5, ECEL1, EFNB1, EFNB2, ELAVL4, ENC1, EPHA7, ERBB4, FGFR2, FNBP1L, FYN, GABRB3, GAP43, GDI1, GNAO1, GPM6A, GPRIN1, HDAC2, HMGB1, HMGB2, HNRNPK, ID1, KIDINS220, KIF3A, L1CAM, LINGO1, LOX, LYPLA1, MAP1B, MAP2, MAP6, MAPK8, MAPT, NCAM1, NEFM, NEUROD1, NFIA, NFIB, NIN, NOTCH1, NSG1,	99

				NTRK2, PAK3, PAX6, PFN2, POU3F2, PPP2CA, PTPRZ1, RAB10, RAB3A, RAPGEF2, RERE, RND2, RNF165, ROBO2, ROBO3, RTN3, RTN4, RUFY3, SKIL, SLC9A6, SPTBN2, STAU2, STMN1, TOP2B, TPBG, TRIM9, UBQLN2, WASF1, WNT3, ZEB2, ZSWIM6	
Cell Morphology	formation	Formation of cellular protrusions	5.63E-21	ACTR2, ACVR2A, AKAP12, APC, ARHGEF7, ASCL1, AUTS2, BASP1, BRSK2, BTBD3, CDC42EP4, CDH2, CDK5R1, CELSR2, CHL1, CHN1, CKB, CLASP2, CNR1, CNTN1, CNTN2, CNTNAP2, CPEB4, CRMP1, CTNNA2, CUX2, CXCR4, DCC, DCLK1, DCX, DPYSL2, DPYSL3, DPYSL5, ECEL1, EFNB1, EFNB2, ELAVL4, ENC1, EPHA3, EPHA7, ERBB4, EXOC5, FASN, FGFR2, FNBP1L, FYN, GABRB3, GAP43, GDI1, GNAO1, GPM6A, GPRIN1, HDAC2, HMGB1, HMGB2, HNRNPK, ID1, ITGB8, KIDINS220, KIF3A, KIF5B, KLF5, L1CAM, LINGO1, LOX, LYPLA1, MAP1B, MAP2, MAP6, MAPK8, MAPT, NCAM1, NEFM, NEUROD1, NFIA, NFIB, NIN, NOTCH1, NRXN1, NSG1, NTRK2, PAK2, PAK3, PAX6, PDPN, PFN2, POU3F2, PPP2CA, PRKACB, PTPRZ1, RAB10, RAB3A, RAPGEF2, RERE, RFX3, RFX4, RND2, RNF165, ROBO2, ROBO3, RTN3, RTN4, RUFY3, S1PR1, SKIL, SLC9A6, SNX10, SPTBN2, STAU2, STMN1, TOP2B, TPBG, TRIM9, TROVE2, TTYH1, UBQLN2, VANGL2, VEGFA, WASF1, WASF3, WASL, WNT3, WWTR1, ZEB2, ZSWIM6	125
Nervous System Development and Function	quantity	Quantity of neurons	8.81E-21	ARHGEF7, ARX, ASCL1, ATF2, CCND2, CDK5R1, CLASP2, CLCN3, CNTN2, CNTNAP2, CUX2, CXCR4, DCC, DCLK1, DLX1, DUSP1, EFNB1, EPHA7, ERBB4, FGFR2, FGFR3, FOXA1, FYN, GNAI2, GNAO1, GNAS, HES5, HOXA3, HOXB3, HSF2, INSM1, IRX3, JAG1, KHDRBS1, KIDINS220, KIF5C, L1CAM, LEF1, MAP1B, MAP6, MAPT, MT-ND6, NCAM1, NEFM, NEUROD1, NEUROD4, NEUROG1, NEUROG2, NHLH1, NHLH2, NOTCH1, NPTX2, NTRK2, OTX2, PAK3, PAX6, PCDH17, PFN2, PTPRZ1, RERE, RGMB, SIM1, SLC12A2, SLC1A3, SLC9A6, SNCG, SOX11, SOX9, SPTBN2, SUB1, VEGFA, WASF1, WNT4, ZBTB18	74
Cellular Development	proliferation	Proliferation of neuronal cells	1.72E-20	APBA2, ARHGEF7, ARX, ASCL1, BASP1, BRSK2, CDH2, CDK5R1, CHL1, CHN1, CLASP2, CNR1, CNTN2, CSNK1E, CXCR4, DCC, DCLK1, DCX, DLL1, DOK6, DPYSL2, DPYSL3, DPYSL5, EFNB1, EFNB2, ELAVL4, ERBB4, EXOC5, FEZF1, FGFR3, FZD3, GAP43, GDAP1, GNAO1, GNAQ, GNAS, GPM6A, GPRIN1, HMGB1, HMGCR, ID1, ID3, IRX3, JAG1, KIF3A, KIF3C, KRAS, L1CAM, LINGO1, MAP1B, MAP2, MAPK8, MAPT, MIB1, NCAM1, NCAN, NEDD4L, NES, NEUROD4, NFIA, NOTCH1, NRXN1, NSG1, NTRK2, OTX1, OTX2, PAX6, PFN2, PTCH1, PTP4A2, PTPRZ1, RBPJ, RND2, ROBO2, RTN4, SET, SKIL, SLC12A2, SOX2, STMN2, TNFRSF19, TOP1, TUBA1A, VCAM1, VEGFA, YWHAZ, ZBTB18	87

Table S11. Downregulated Genes at Day 35 - Embryonic Development.

Category	Function	Function Annotation	p-value	Molecules	# Molecules
Embryonic Development	formation	Formation of brain	1.62E-29	ARX, ASCL1, ATAT1, BHLHE22, BTBD3, CCND2, CDH2, CDK5R1, CDK6, CELSR2, CHL1, CNBP, CNR1, CNTN1, CNTNAP2, CTNNA2, CXADR, CXCR4, DCLK1, DCX, DLL1, DLX1, DRAXIN, EBF3, EGLN1, EPHA7, ERBB4, FEZF1, FGFR2, FGFR3, FYN, FZD3, GAP43, GNAO1, HDAC2, HES5, HNRNPK, HOXA1, HOXB1, HOXB2, IPMK, IRX3, KDM6B, KHDRBS1, KIF3A, L1CAM, LEF1, MAP1B, MAPK8, MAPT, MARCKSL1, MGEA5, MSX1, MYCN, NCAM1, NEUROD1, NEUROD4, NEUROG2, NFIA, NFIB, NIN, NOTCH1, NPAS3, NR2F2, NTRK2, OTX1, OTX2, PAX6, PLCB1, POU3F2, POU3F3, PRDM8, PROX1, PTCH1, RAPGEF2, RARB, RERE, RFX4, ROBO2, ROBO3, S1PR1, SIM1, SLC1A2, SLC1A3, SLC4A7, SMAD9, SOX1, SOX2, SOX4, STXBP1, TDG, TOP2B, VEGFA, WLS, YBX1, ZBTB18, ZEB2, ZIC1, ZIC4	99
Embryonic Development	development	Development of head	1.61E-28	ACVR2B, ARID5B, ARX, ASCL1, ATAT1, AXIN2, BHLHE22, BTBD3, CCND2, CDH2, CDK5R1, CDK6, CELSR2, CHL1, CLCN3, CLIC4, CNBP, CNR1, CNTN1, CNTNAP2, CTNNA2, CXADR, CXCR4, CYP26B1, DCLK1, DCX, DHRS3, DLL1, DLX1, DRAXIN, EBF1, EBF3, EGLN1, EPHA7, ERBB4, FEZF1, FGFR2, FGFR3, FYN, FZD3, GABRA5, GABRB3, GAP43, GNAO1, GNAQ, GPC3, HDAC2, HES5, HES6, HEY1, HNRNPK, HOXA1, HOXB1, HOXB2, HOXB3, HS6ST1, ID1, ID3, INSIG1, INSIG2, IPMK, IRX3, JAG1, KDM1A, KDM6B, KHDRBS1, KIF3A, KRAS, L1CAM, LEF1, LIN7A, MAP1B, MAP2, MAPK8, MAPT, MARCKSL1, MEIS2, MGEA5, MSX1, MYCN, NCAM1, NES, NEUROD1, NEUROD4, NEUROG1, NEUROG2, NFIA, NFIB, NIN, NKD1, NOTCH1, NPAS3, NR2F2, NR3C1, NRAS, NTRK2, OTX1, OTX2, PAX6, PHC2, PIK3R1, PLCB1, POU3F2, POU3F3, PRDM8, PROM1, PROX1, PSIP1, PTCH1, PYGO1, RAD23B, RAPGEF2, RARB, RBPJ, RERE, RFX4, ROBO2, ROBO3, S1PR1, SIM1, SLC1A2, SLC1A3, SLC4A7, SMAD9, SOX1, SOX11, SOX2, SOX4, SOX9, SSBP3, STAU2, STC1, STXBP1, TCF7L2, TDG, TOP2B, TSHZ1, VANGL2, VCAM1, VEGFA, WLS, WNT3, YBX1, ZBTB18, ZEB1, ZEB2, ZFAND5, ZIC1, ZIC4	149
Embryonic Development	development	Development of cerebral cortex	2.65E-20	ASCL1, ATAT1, BTBD3, CDH2, CDK5R1, CDK6, CHL1, CNR1, CXADR, DCX, DLL1, DLX1, ERBB4, FGFR2, FGFR3, FYN, GAP43, HDAC2, HNRNPK, KDM6B, KIF3A, L1CAM, LEF1, MAPT, MGEA5, NCAM1, NEUROD1, NEUROG2, NFIB, NTRK2, OTX1, OTX2,	42

				PAX6, PLCB1, POU3F2, POU3F3, PROX1, SLC1A2, SLC1A3, SOX2, ZBTB18, ZEB2	
Embryonic Development	formation	Formation of forebrain	1.02E-18	ARX, ASCL1, BHLHE22, CDH2, CDK5R1, CDK6, CNBP, CXCR4, DCLK1, DLX1, DRAXIN, EBF3, FEZF1, FGFR3, FYN, GAP43, GNAO1, HES5, IRX3, L1CAM, MAP1B, MSX1, MYCN, NCAM1, NEUROG2, NFIA, NFIB, NIN, NOTCH1, NR2F2, NTRK2, OTX1, OTX2, PAX6, POU3F2, POU3F3, PRDM8, PTCH1, RARB, RFX4, ROBO2, SIM1, SLC1A2, SOX1, SOX2, TDG, TOP2B, VEGFA, ZEB2, ZIC1	50
Embryonic Development	patterning	Patterning of rostrocaudal axis	6.58E-14	ACVR2A, ACVR2B, APC, HOXA1, HOXA3, HOXA5, HOXB1, HOXB2, HOXB3, HOXB4, HOXB5, HOXB6, HOXB7, HOXB8, HOXB9, HOXD3, KIF3A, MSX1, NEUROD1, NR2F2, OTX1, OTX2, PAX6, TSHZ1, VANGL2, WNT3	26
Embryonic Development	formation	Formation of telencephalon	7.26E-14	ARX, ASCL1, BHLHE22, CDH2, CDK5R1, CDK6, CXCR4, DLX1, EBF3, FEZF1, GAP43, GNAO1, HES5, L1CAM, MAP1B, NEUROG2, NFIA, NFIB, NIN, NTRK2, OTX2, PAX6, POU3F2, POU3F3, PRDM8, PTCH1, RARB, RFX4, ROBO2, SLC1A2, TDG, TOP2B, ZEB2	33
Embryonic Development	formation	Formation of rhombencephal on	4.88E-13	ASCL1, CCND2, CDK5R1, CNTN1, CXCR4, FGFR3, FYN, HOXA1, HOXB1, KHDRBS1, L1CAM, MGEA5, NEUROD1, NEUROD4, NFIA, NFIB, NPAS3, NTRK2, OTX1, OTX2, PAX6, POU3F2, POU3F3, RERE, RFX4, SLC4A7, SMAD9, SOX4, WLS, ZBTB18, ZIC1, ZIC4	32
Embryonic Development	patterning	Patterning of body axis	1.39E-12	ACVR2A, ACVR2B, APC, HOXA1, HOXA3, HOXA5, HOXB1, HOXB2, HOXB3, HOXB4, HOXB5, HOXB6, HOXB7, HOXB8, HOXB9, HOXD3, KIF3A, MSX1, NEUROD1, NR2F2, OTX1, OTX2, PAX6, RFX3, TSHZ1, VANGL2, WNT3	27
Embryonic Development	development	Development of sensory organ	1.62E-11	ASCL1, CLCN3, CLIC4, CYP26B1, DLL1, DLX1, EBF1, EBF3, FGFR2, FGFR3, FYN, FZD3, GNAO1, HES5, HES6, HEY1, HOXA1, HS6ST1, ID1, ID3, INSIG1, INSIG2, IRX3, JAG1, KDM6B, KRAS, LEF1, LIN7A, MAP1B, MAP2, MAPK8, MAPT, MARCKSL1, MEIS2, MSX1, MYCN, NCAM1, NES, NEUROD1, NEUROD4, NEUROG1, NEUROG2, NKD1, NOTCH1, NR3C1, NRAS, NTRK2, OTX1, OTX2, PAX6, PIK3R1, PROM1, PROX1, PSIP1, PYGO1, RAD23B, RARB, RBPJ, SLC1A3, SLC4A7, SOX11, SOX2, SOX4, SOX9, STAU2, STC1, TSHZ1, VANGL2, VCAM1, VEGFA, ZEB1	71
Embryonic Development	development	Development of metencephalon	6.33E-11	CCND2, CDK5R1, CNTN1, CXCR4, FGFR3, FYN, KHDRBS1, L1CAM, MGEA5, NEUROD1, NEUROD4, NFIA, NPAS3, NTRK2, OTX1, OTX2, PAX6, POU3F2, POU3F3, RERE, RFX4, SLC4A7, SOX4, ZBTB18, ZIC1, ZIC4	26
Embryonic Development	branching	Branching of neurites	1.1E-10	ARHGEF7, CDH2, CHN1, CLASP2, CNR1, CNTNAP2, CPEB4, CRMP1, CTNNA2, DCX, DPYSL2, DPYSL5, EFNB1, ELAVL4, ERBB4, GABRB3, GAP43, GNAO1, HMGB1, HMGB2, HNRNPK, KIDINS220, KIF3A, L1CAM, LYPLA1, MAP1B, MAPT, NCAM1, NEFM, NEUROD1, NOTCH1, NSG1, NTRK2, PFN2, RAPGEF2, RND2, ROBO2, RTN4, SKIL, SLC9A6, SPTBN2, STAU2, TPBG, TRIM9, UBQLN2	45
Embryonic Development	branching	Dendritic growth/branchin g	4.3E-10	ARHGEF7, CDH2, CHN1, CLASP2, CNR1, CNTNAP2, CPEB4, CRMP1, CTNNA2, DCX, DPYSL5, EFNB1, ELAVL4, ERBB4, GABRB3, GAP43, GNAO1, HNRNPK, KIDINS220, KIF3A, L1CAM, LYPLA1, MAPT, NEFM, NEUROD1, NOTCH1, NSG1, NTRK2, PFN2, RAPGEF2, RND2, ROBO2, RTN4, SLC9A6, SPTBN2, STAU2, TPBG, UBQLN2	38
Embryonic Development	morphogenesis	Morphogenesis of head	4.71E-10	ARID5B, BHLHE22, CDH2, CTNNA2, CYP26B1, FGFR2, FYN, FZD3, HOXA1, INSIG1, INSIG2, IRX3, JAG1, L1CAM, LEF1, MSX1, NEUROD1, NEUROD4, NEUROG1, NFIB, NIN, OTX1, OTX2, PAX6, PRDM8, RARB, RFX4, SOX2, SOX9, SSBP3, STAU2, TCF7L2, TSHZ1, VANGL2, VEGFA, WNT3, ZEB1, ZEB2	38
Embryonic Development	formation	Formation of cerebellum	1.21E-09	CCND2, CDK5R1, CNTN1, CXCR4, FGFR3, FYN, KHDRBS1, L1CAM, NEUROD1, NEUROD4, NFIA, NPAS3, NTRK2, OTX2, PAX6, POU3F2, POU3F3, RERE, RFX4, SLC4A7, SOX4, ZBTB18, ZIC1, ZIC4	24
Embryonic Development	formation	Formation of eye	4.97E-08	ASCL1, CLCN3, CLIC4, DLX1, EBF1, EBF3, FGFR2, FYN, HES5, HES6, HS6ST1, ID1, ID3, IRX3, JAG1, KDM6B, KRAS, LEF1, MAP1B, MAPK8, MAPT, MARCKSL1, MEIS2, NCAM1, NEUROD1, NEUROD4, NEUROG2, NKD1, NOTCH1, NR3C1, NRAS, NTRK2, OTX1, OTX2, PAX6, PIK3R1, PROM1, PSIP1, PYGO1, RAD23B, RARB, RBPJ, SLC1A3, SLC4A7, SOX11, SOX4, SOX9, STAU2, STC1, VCAM1, VEGFA, ZEB1	52
Embryonic Development	development	Development of spinal cord	0.000000111	ASCL1, DLL1, DRAXIN, HOXB8, NTRK2, PAX6, PBX3, PROX1, RFX4, SOX11, SOX4, TOP2B, ZIC1	13
Embryonic Development	differentiation	Differentiation of photoreceptors	0.000000165	ASCL1, DLX1, EBF1, EBF3, HES6, KDM6B, NKD1, NOTCH1, SOX9	9
Embryonic Development	development	Development of inner ear	0.00000042	DLL1, FGFR2, FGFR3, FZD3, HES5, HEY1, HOXA1, INSIG1, INSIG2, JAG1, LIN7A, MYCN, NEUROD1, NEUROG1, NOTCH1, OTX1, OTX2, PROX1, SOX2, SOX9, VANGL2, ZEB1	22
Embryonic Development	formation	Formation of cortical lamina	0.000000501	CDK5R1, FGFR2, FGFR3, GAP43, NTRK2, PAX6	6

Embryonic Development	morphogenesis	Morphogenesis of forebrain	0.00000054	BHLHE22, CDH2, IRX3, NFIB, NIN, OTX1, OTX2, PRDM8, SOX2, ZEB2	10
Embryonic Development	development	Development of rhombomere	0.000000858	FGFR2, FGFR3, FZD3, HOXA1, HOXB1, HOXB2, OTX2	7
Embryonic Development	size	Size of embryonic tissue	0.000000898	CXADR, EPHA3, ERBB4, FGFR3, GNAO1, HOXA1, HOXB1, MSX1, MYCN, NOTCH1, PDPN, PRKAR1A, PTCH1, ZEB2	14
Embryonic Development	morphogenesis	Morphogenesis of embryo	0.00000192	ACVR2B, ATF2, CDH4, CITED2, CYP26B1, DHRS3, FZD3, GNAQ, GNAS, GPC3, HDAC2, HEY1, IRX3, LEF1, LNPK, MSX1, MYCN, NOTCH1, PTCH1, RARB, RBPJ, RNF165, SALL3, SOX11, TCF7L2, VEGFA, WNT3, WNT4, ZEB1, ZEB2	30
Embryonic Development	formation	Formation of brain cells	0.00000202	ARX, ASCL1, CHL1, CNR1, CXCR4, FGFR2, KIF3A, L1CAM, NCAM1, NEUROD1, NEUROG2, NOTCH1, POU3F2	13

TableS12. Downregulated Genes at Day35 - Eye related.

Category	Function	Function Annotation	p-value	Molecules	# Molecules
Visual System Development and Function	differentiation	Differentiation of retinal cells	5.91E-10	ASCL1, DLX1, EBF1, EBF3, HES6, KDM6B, NEUROD1, NEUROD4, NKD1, NOTCH1, OTX1, OTX2, SOX9	13
Visual System Development and Function	formation	Formation of eye	4.97E-08	ASCL1, CLCN3, CLIC4, DLX1, EBF1, EBF3, FGFR2, FYN, HES5, HES6, HS6ST1, ID1, ID3, IRX3, JAG1, KDM6B, KRAS, LEF1, MAP1B, MAPK8, MAPT, MARCKSL1, MEIS2, NCAM1, NEUROD1, NEUROD4, NEUROG2, NKD1, NOTCH1, NR3C1, NRAS, NTRK2, OTX1, OTX2, PAX6, PIK3R1, PROM1, PSIP1, PYGO1, RAD23B, RARB, RBPJ, SLC1A3, SLC4A7, SOX11, SOX4, SOX9, STAU2, STC1, VCAM1, VEGFA, ZEB1	52
Visual System Development and Function	differentiation	Differentiation of photoreceptors	1.65E-07	ASCL1, DLX1, EBF1, EBF3, HES6, KDM6B, NKD1, NOTCH1, SOX9	9
Visual System Development and Function	development	Development of lacrimal gland	2.06E-06	FGFR2, HS6ST1, OTX1, OTX2, PAX6, SOX9	6
Visual System Development and Function	differentiation	Differentiation of retinal cells	5.91E-10	ASCL1, DLX1, EBF1, EBF3, HES6, KDM6B, NEUROD1, NEUROD4, NKD1, NOTCH1, OTX1, OTX2, SOX9	13
Visual System Development and Function	formation	Formation of eye	4.97E-08	ASCL1, CLCN3, CLIC4, DLX1, EBF1, EBF3, FGFR2, FYN, HES5, HES6, HS6ST1, ID1, ID3, IRX3, JAG1, KDM6B, KRAS, LEF1, MAP1B, MAPK8, MAPT, MARCKSL1, MEIS2, NCAM1, NEUROD1, NEUROD4, NEUROG2, NKD1, NOTCH1, NR3C1, NRAS, NTRK2, OTX1, OTX2, PAX6, PIK3R1, PROM1, PSIP1, PYGO1, RAD23B, RARB, RBPJ, SLC1A3, SLC4A7, SOX11, SOX4, SOX9, STAU2, STC1, VCAM1, VEGFA, ZEB1	52

Table S13. Downregulated Genes at Day35 - Nervous System Development and Function.

Category	Function	Function Annotation	p-value	Molecules	# Molecules
Nervous System Development and Function	development	Development of central nervous system	4.12E-35	ARX, ASCL1, ATAT1, BHLHE22, BTBD3, CCND2, CDH2, CDK5R1, CDK6, CELSR2, CHL1, CITED2, CNBP, CNR1, CNTN1, CNTNAP2, CTNNA2, CXADR, CXCR4, DCLK1, DCX, DLL1, DLX1, DRAXIN, EBF3, EGLN1, EPHA7, ERBB4, FEZF1, FGFR2, FGFR3, FUBP1, FYN, FZD3, GAP43, GNAO1, HDAC2, HES5, HEY1, HNRNPK, HOXA1, HOXB1, HOXB2, HOXB8, IPMK, IRX3, KDM6B, KHDRBS1, KIF3A, KRAS, L1CAM, LEF1, LINGO1, MAP1B, MAP2, MAPK8, MAPT, MARCKSL1, MGEA5, MSX1, MYCN, MYT1, NCAM1, NEUROD1, NEUROD4, NEUROG1, NEUROG2, NFIA, NFIB, NHLH1, NHLH2, NIN, NOTCH1, NPAS3, NR2F2, NR3C1, NTRK2, OTX1, OTX2, PAX6, PBX3, PLCB1, POU3F2, POU3F3, PRDM8, PROX1, PTCH1, PTPRZ1, QKI, RAPGEF2, RARB, RBPJ, RERE, RFX4, ROBO2, ROBO3, RPL24, RTN4, S1PR1, SIM1, SLC1A2, SLC1A3, SLC4A7, SMAD9, SOX1, SOX11, SOX2, SOX4, SOX9, STXBP1, TAGLN3, TCF7L2, TDG, TOP2B, VEGFA, WASF3, WLS, YBX1, ZBTB18, ZEB1, ZEB2, ZIC1, ZIC4	123
Nervous System Development and Function	formation	Formation of brain	1.62E-29	ARX, ASCL1, ATAT1, BHLHE22, BTBD3, CCND2, CDH2, CDK5R1, CDK6, CELSR2, CHL1, CNBP, CNR1, CNTN1, CNTNAP2, CTNNA2, CXADR, CXCR4, DCLK1, DCX, DLL1, DLX1, DRAXIN, EBF3, EGLN1, EPHA7, ERBB4, FEZF1, FGFR2, FGFR3, FYN, FZD3, GAP43, GNAO1, HDAC2, HES5, HNRNPK, HOXA1, HOXB1, HOXB2, IPMK, IRX3, KDM6B, KHDRBS1, KIF3A, L1CAM, LEF1, MAP1B, MAPK8, MAPT, MARCKSL1, MGEA5, MSX1, MYCN, NCAM1, NEUROD1, NEUROD4, NEUROG2, NFIA, NFIB,	99

				NIN, NOTCH1, NPAS3, NR2F2, NTRK2, OTX1, OTX2, PAX6, PLCB1, POU3F2, POU3F3, PRDM8, PROX1, PTCH1, RAPGEF2, RARB, RERE, RFX4, ROBO2, ROBO3, S1PR1, SIM1, SLC1A2, SLC1A3, SLC4A7, SMAD9, SOX1, SOX2, SOX4, STXBP1, TDG, TOP2B, VEGFA, WLS, YBX1, ZBTB18, ZEB2, ZIC1, ZIC4	
Nervous System Development and Function	differentiation	Differentiation of neurons	4.27E-28	ACSL4, ADNP2, ARHGEF2, ARX, ASCL1, BHLHE22, BRINP1, BRSK2, C8orf46, CBLN1, CDK5R1, CNR1, CNTN2, CYP26B1, DLL1, DLX1, DPYSL2, EBF1, EBF3, EFNB1, ELAVL2, ELAVL3, ELAVL4, ERBB4, FEZF1, FGFR2, FGFR3, FOXA1, GAP43, GPC2, HDAC2, HES5, HEY1, HNRNPK, HOXA1, HOXB1, HOXB2, HOXD3, ID3, INSM1, IRX3, JAG1, KDM6B, KIDINS220, LEF1, MAP1B, MAPK8, MAPT, MIB1, MMD, NCAM1, NEUROD1, NEUROD4, NEUROG1, NEUROG2, NOTCH1, NREP, NRXN1, NTRK2, OLIG3, OTX2, PAX6, POU3F2, PROX1, PTPRZ1, RARB, RBPJ, RTN4, S1PR1, SALL3, SLC1A2, SLC1A3, SOX1, SOX11, SOX2, SOX4, SPAG9, TCF12, TRIM9, VEGFA, WNT3, YWHAG, ZBTB18, ZEB1, ZNF536	85
Nervous System Development and Function	morphogenesis	Morphogenesis of neurons	3.3E-23	ACVR2A, APC, ARHGEF7, ASCL1, BASP1, BRSK2, BTBD3, CDH2, CDK5R1, CELSR2, CHL1, CHN1, CKB, CLASP2, CNR1, CNTN1, CNTN2, CNTNAP2, CPEB4, CRMP1, CTNNA2, CUX2, DCC, DCLK1, DCX, DPYSL2, DPYSL3, DPYSL5, ECEL1, EFNB1, EFNB2, ELAVL4, ENC1, EPHA7, ERBB4, FGFR2, FNBP1L, FYN, GABRB3, GAP43, GDI1, GNAO1, GPM6A, GPRIN1, HDAC2, HMGB1, HMGB2, HNRNPK, ID1, KIDINS220, KIF3A, L1CAM, LINGO1, LOX, LYPLA1, MAP1B, MAP2, MAP6, MAPK8, MAPT, NCAM1, NEFM, NEUROD1, NFIA, NFIB, NIN, NOTCH1, NSG1, NTRK2, PAK3, PAX6, PFN2, POU3F2, PPP2CA, PTPRZ1, RAB10, RAB3A, RAPGEF2, RERE, RND2, RNF165, ROBO2, ROBO3, RTN3, RTN4, RUFY3, SKIL, SLC1A3, SLC9A6, SPTBN2, STAU2, STMN1, TOP2B, TPBG, TRIM9, UBQLN2, WASF1, WNT3, ZEB2, ZSWIM6	100
Nervous System Development and Function	neuritogenesis	Neuritogenesis	4.68E-23	ACVR2A, APC, ARHGEF7, ASCL1, BASP1, BRSK2, BTBD3, CDH2, CDK5R1, CELSR2, CHL1, CHN1, CKB, CLASP2, CNR1, CNTN1, CNTN2, CNTNAP2, CPEB4, CRMP1, CTNNA2, CUX2, DCC, DCLK1, DCX, DPYSL2, DPYSL3, DPYSL5, ECEL1, EFNB1, EFNB2, ELAVL4, ENC1, EPHA7, ERBB4, FGFR2, FNBP1L, FYN, GABRB3, GAP43, GDI1, GNAO1, GPM6A, GPRIN1, HDAC2, HMGB1, HMGB2, HNRNPK, ID1, KIDINS220, KIF3A, L1CAM, LINGO1, LOX, LYPLA1, MAP1B, MAP2, MAP6, MAPK8, MAPT, NCAM1, NEFM, NEUROD1, NFIA, NFIB, NIN, NOTCH1, NSG1, NTRK2, PAK3, PAX6, PFN2, POU3F2, PPP2CA, PTPRZ1, RAB10, RAB3A, RAPGEF2, RERE, RND2, RNF165, ROBO2, ROBO3, RTN3, RTN4, RUFY3, SKIL, SLC9A6, SPTBN2, STAU2, STMN1, TOP2B, TPBG, TRIM9, UBQLN2, WASF1, WNT3, ZEB2, ZSWIM6	99
Nervous System Development and Function	proliferation	Proliferation of neuronal cells	1.72E-20	APBA2, ARHGEF7, ARX, ASCL1, BASP1, BRSK2, CDH2, CDK5R1, CHL1, CHN1, CLASP2, CNR1, CNTN2, CSNK1E, CXCR4, DCC, DCLK1, DCX, DLL1, DOK6, DPYSL2, DPYSL3, DPYSL5, EFNB1, EFNB2, ELAVL4, ERBB4, EXOC5, FEZF1, FGFR3, FZD3, GAP43, GDAP1, GNAO1, GNAQ, GNAS, GPM6A, GPRIN1, HMGB1, HMGCR, ID1, ID3, IRX3, JAG1, KIF3A, KIF3C, KRAS, L1CAM, LINGO1, MAP1B, MAP2, MAPK8, MAPT, MIB1, NCAM1, NCAN, NEDD4L, NES, NEUROD4, NFIA, NOTCH1, NRXN1, NSG1, NTRK2, OTX1, OTX2, PAX6, PFN2, PTCH1, PTP4A2, PTPRZ1, RBPJ, RND2, ROBO2, RTN4, SET, SKIL, SLC12A2, SOX2, STMN2, TNFRSF19, TOP1, TUBA1A, VCAM1, VEGFA, YWHAZ, ZBTB18	87
Nervous System Development and Function	development	Development of cerebral cortex	2.65E-20	ASCL1, ATAT1, BTBD3, CDH2, CDK5R1, CDK6, CHL1, CNR1, CXADR, DCX, DLL1, DLX1, ERBB4, FGFR2, FGFR3, FYN, GAP43, HDAC2, HNRNPK, KDM6B, KIF3A, L1CAM, LEF1, MAPT, MGEA5, NCAM1, NEUROD1, NEUROG2, NFIB, NTRK2, OTX1, OTX2, PAX6, PLCB1, POU3F2, POU3F3, PROX1, SLC1A2, SLC1A3, SOX2, ZBTB18, ZEB2	42
Nervous System Development and Function	formation	Formation of forebrain	1.02E-18	ARX, ASCL1, BHLHE22, CDH2, CDK5R1, CDK6, CNBP, CXCR4, DCLK1, DLX1, DRAXIN, EBF3, FEZF1, FGFR3, FYN, GAP43, GNAO1, HES5, IRX3, L1CAM, MAP1B, MSX1, MYCN, NCAM1, NEUROG2, NFIA, NFIB, NIN, NOTCH1, NR2F2, NTRK2, OTX1, OTX2, PAX6, POU3F2, POU3F3, PRDM8, PTCH1, RARB, RFX4, ROBO2, SIM1, SLC1A2, SOX1, SOX2, TDG, TOP2B, VEGFA, ZEB2, ZIC1	50
Nervous System Development and Function	guidance	Guidance of axons	2.72E-14	ANOS1, ARX, BHLHE22, CDH2, CDH4, CDK5R1, CHL1, CHN1, CNTN1, CNTN2, CXCR4, DCC, DCLK1, DCX, DPYSL5, DRAXIN, EFNB1, ERBB4, FEZF1, FYN, GAP43, HOXA1, KIF5C, L1CAM, NEUROG2, NRXN1, OTX2, PAX6, RNF165, ROBO2, ROBO3, SEMA3C, WNT3	33
Nervous System Development and Function	formation	Formation of telencephalon	7.26E-14	ARX, ASCL1, BHLHE22, CDH2, CDK5R1, CDK6, CXCR4, DLX1, EBF3, FEZF1, GAP43, GNAO1, HES5, L1CAM, MAP1B, NEUROG2, NFIA, NFIB, NIN, NTRK2, OTX2, PAX6, POU3F2, POU3F3, PRDM8, PTCH1, RARB, RFX4, ROBO2, SLC1A2, TDG, TOP2B, ZEB2	33
Nervous System	formation	Formation of rhombencephalon	4.88E-13	ASCL1, CCND2, CDK5R1, CNTN1, CXCR4, FGFR3, FYN, HOXA1, HOXB1, KHDRBS1, L1CAM, MGEA5, NEUROD1,	32

Development and Function				NEUROD4, NFIA, NFIB, NPAS3, NTRK2, OTX1, OTX2, PAX6, POU3F2, POU3F3, RERE, RFX4, SLC4A7, SMAD9, SOX4, WLS, ZBTB18, ZIC1, ZIC4	
Nervous System Development and Function	development	Development of metencephalon	6.33E-11	CCND2, CDK5R1, CNTN1, CXCR4, FGFR3, FYN, KHDRBS1, L1CAM, MGEA5, NEUROD1, NEUROD4, NFIA, NPAS3, NTRK2, OTX1, OTX2, PAX6, POU3F2, POU3F3, RERE, RFX4, SLC4A7, SOX4, ZBTB18, ZIC1, ZIC4	26
Nervous System Development and Function	branching	Branching of neurites	1.1E-10	ARHGEF7, CDH2, CHN1, CLASP2, CNR1, CNTNAP2, CPEB4, CRMP1, CTNNA2, DCX, DPYSL2, DPYSL5, EFNB1, ELAVL4, ERBB4, GABRB3, GAP43, GNAO1, HMGB1, HMGB2, HNRNPK, KIDINS220, KIF3A, L1CAM, LYPLA1, MAP1B, MAPT, NCAM1, NEFM, NEUROD1, NOTCH1, NSG1, NTRK2, PFN2, RAPGEF2, RND2, ROBO2, RTN4, SKIL, SLC9A6, SPTBN2, STAU2, TPBG, TRIM9, UBQLN2	45
Nervous System Development and Function	development	Sensory system development	2.32E-10	ASCL1, CDH2, CLCN3, CLIC4, CXCR4, DLX1, EBF1, EBF3, FGFR2, FGFR3, FYN, GNAO1, HES5, HES6, HOXA1, HOXA3, HOXB3, HS6ST1, ID1, ID3, IRX3, JAG1, KDM6B, KIF5C, KRAS, LEF1, MAP1B, MAPK8, MAPT, MARCKSL1, MEIS2, MIB1, NCAM1, NEUROD1, NEUROD4, NEUROG2, NKD1, NOTCH1, NR3C1, NRAS, NTRK2, OTX1, OTX2, PAX6, PIK3R1, PROM1, PSIP1, PYGO1, RAD23B, RARB, RBPJ, SLC12A2, SLC1A3, SLC4A7, SOX11, SOX4, SOX9, STAU2, STC1, VCAM1, VEGFA, ZEB1	62
Nervous System Development and Function	branching	Dendritic growth/branching	4.3E-10	ARHGEF7, CDH2, CHN1, CLASP2, CNR1, CNTNAP2, CPEB4, CRMP1, CTNNA2, DCX, DPYSL5, EFNB1, ELAVL4, ERBB4, GABRB3, GAP43, GNAO1, HNRNPK, KIDINS220, KIF3A, L1CAM, LYPLA1, MAPT, NEFM, NEUROD1, NOTCH1, NSG1, NTRK2, PFN2, RAPGEF2, RND2, ROBO2, RTN4, SLC9A6, SPTBN2, STAU2, TPBG, UBQLN2	38
Nervous System Development and Function	formation	Formation of cerebellum	1.21E-09	CCND2, CDK5R1, CNTN1, CXCR4, FGFR3, FYN, KHDRBS1, L1CAM, NEUROD1, NEUROD4, NFIA, NPAS3, NTRK2, OTX2, PAX6, POU3F2, POU3F3, RERE, RFX4, SLC4A7, SOX4, ZBTB18, ZIC1, ZIC4	24
Nervous System Development and Function	differentiation	Differentiation of neuroglia	1.69E-09	ASCL1, CDC25B, CDH2, CNTN1, CXCR4, DLL3, DLX1, FGFR3, GAP43, HDAC2, HES5, KHDRBS1, LINGO1, MYCN, NEUROD4, NEUROG1, NEUROG2, NFIA, NFIB, NOTCH1, NTRK2, OTX2, PAX6, PTPRZ1, QKI, RTN4, S1PR1, SOX9, TCF7L2, ZBTB18	30
Nervous System Development and Function	development	Development of neuroglia	2.86E-09	ASCL1, CDK6, DLL1, DLX1, ERBB4, FGFR2, FGFR3, HES5, HEY1, KRAS, NEUROD4, NEUROG2, NOTCH1, POU3F2, RBPJ, SOX11, SOX4, TCF7L2, WASF3	19
Nervous System Development and Function	growth	Growth of brain	7.08E-09	ARX, CNR1, CUX2, FGFR2, FGFR3, GAP43, GNAS, HLTF, ID1, ID3, KIF3A, MAPT, NEUROD1, NPAS3, PAX6, RTN4, SOX11, SOX2, VEGFA, ZBTB18, ZIC1, ZNF503	22
Nervous System Development and Function	development	Development of central nervous system cells	9.52E-09	ARX, ASCL1, CDK6, CHL1, CNR1, CXCR4, DLL1, ERBB4, FGFR2, HEY1, KIF3A, KRAS, L1CAM, NCAM1, NEUROD1, NEUROG2, NOTCH1, NTRK2, POU3F2, RBPJ	20
Nervous System Development and Function	agenesis	Agenesis of corpus callosum	0.000000012	ARX, BHLHE22, DCC, DCLK1, DRAXIN, EFNB1, L1CAM, MAP1B, MARCKSL1, NFIA, NFIB	11
Nervous System Development and Function	formation	Formation of dendrites	1.33E-08	BTBD3, CDH2, CELSR2, CKB, CTNNA2, CUX2, DCLK1, DCX, EFNB1, ELAVL4, FYN, HDAC2, ID1, KIDINS220, L1CAM, LOX, MAP1B, MAP2, MAP6, MAPK8, NFIA, NTRK2, PAK3, PTPRZ1, RAPGEF2, RERE	26
Nervous System Development and Function	formation	Formation of eye	4.97E-08	ASCL1, CLCN3, CLIC4, DLX1, EBF1, EBF3, FGFR2, FYN, HES5, HES6, HS6ST1, ID1, ID3, IRX3, JAG1, KDM6B, KRAS, LEF1, MAP1B, MAPK8, MAPT, MARCKSL1, MEIS2, NCAM1, NEUROD1, NEUROD4, NEUROG2, NKD1, NOTCH1, NR3C1, NRAS, NTRK2, OTX1, OTX2, PAX6, PIK3R1, PROM1, PSIP1, PYGO1, RAD23B, RARB, RBPJ, SLC1A3, SLC4A7, SOX11, SOX4, SOX9, STAU2, STC1, VCAM1, VEGFA, ZEB1	52
Nervous System Development and Function	differentiation	Differentiation of oligodendrocytes	6.08E-08	ASCL1, CNTN1, CXCR4, DLX1, FGFR3, HDAC2, HES5, KHDRBS1, LINGO1, NEUROG1, NOTCH1, NTRK2, OTX2, PTPRZ1, QKI, RTN4, SOX9, TCF7L2, ZBTB18	19
Nervous System Development and Function	proliferation	Proliferation of brain cells	7.07E-08	ARX, CNR1, CUX2, GAP43, GNAS, HLTF, ID1, ID3, KIF3A, MAPT, NEUROD1, NPAS3, PAX6, RTN4, SOX11, SOX2, VEGFA, ZBTB18, ZIC1, ZNF503	20
Nervous System Development and Function	maturation	Maturation of neurons	7.65E-08	ASCL1, CDKN1C, CNR1, DCLK1, EFNB1, EPHA7, ERBB4, HDAC2, MAPT, NEUROD1, NFIA, NRXN1, RAB3A, SEZ6L, SLC12A2, STXBP1	16
Nervous System Development and Function	development	Development of spinal cord	0.000000111	ASCL1, DLL1, DRAXIN, HOXB8, NTRK2, PAX6, PBX3, PROX1, RFX4, SOX11, SOX4, TOP2B, ZIC1	13

Nervous System Development and Function	differentiation	Differentiation of photoreceptors	0.000000165	ASCL1, DLX1, EBF1, EBF3, HES6, KDM6B, NKD1, NOTCH1, SOX9	9
Nervous System Development and Function	quantity	Quantity of sensory neurons	0.000000242	ASCL1, CXCR4, FGFR3, HES5, HOXA3, HOXB3, JAG1, KIF5C, NEUROD1, NEUROD4, NEUROG2, NOTCH1, NTRK2, SLC12A2, SLC1A3	15
Nervous System Development and Function	proliferation	Proliferation of neural precursor cells	0.000000242	ARX, CNR1, CUX2, GAP43, HLTF, ID1, ID3, KIF3A, NPAS3, PAX6, SOX11, SOX2, VEGFA, ZBTB18, ZNF503	15
Nervous System Development and Function	formation	Formation of cortical lamina	0.000000501	CDK5R1, FGFR2, FGFR3, GAP43, NTRK2, PAX6	6
Nervous System Development and Function	morphogenesis	Morphogenesis of forebrain	0.00000054	BHLHE22, CDH2, IRX3, NFIB, NIN, OTX1, OTX2, PRDM8, SOX2, ZEB2	10
Nervous System Development and Function	innervation	Innervation	0.00000057	CBLN1, CDK5R1, CHN1, DCC, ECEL1, ERBB4, FGFR3, GABRA5, GABRB3, GAP43, GNAQ, L1CAM, NEUROG1, NEUROG2, NPTX2, NTRK2, RNF165, ROBO2, SEZ6L, SMAD9, TOP2B, VCAM1	22
Nervous System Development and Function	outgrowth	Outgrowth of axons	0.000000675	BRSK2, CDK5R1, CHN1, DCC, DPYSL2, ELAVL4, FEZF1, GAP43, GDAP1, GPM6A, KIF3C, L1CAM, MAP1B, MAPT, NFIA, RTN4, VEGFA, ZBTB18	18
Nervous System Development and Function	foliation	Foliation of cerebellum	0.000000765	CCND2, CDK5R1, KHDRBS1, L1CAM, NFIA, NPAS3, POU3F2, POU3F3, ZBTB18, ZIC1, ZIC4	11
Nervous System Development and Function	formation	Formation of brain cells	0.00000202	ARX, ASCL1, CHL1, CNR1, CXCR4, FGFR2, KIF3A, L1CAM, NCAM1, NEUROD1, NEUROG2, NOTCH1, POU3F2	13

Table S14. Downregulated Genes at D35 - Tissue and Organ Development.

Category	Function	Function Annotation	p-value	Molecules	# Molecules
Tissue Development	formation	Formation of brain	1.62E-29	ARX, ASCL1, ATAT1, BHLHE22, BTBD3, CCND2, CDH2, CDK5R1, CDK6, CELSR2, CHL1, CNBP, CNR1, CNTN1, CNTNAP2, CTNNA2, CXADR, CXCR4, DCLK1, DCX, DLL1, DLX1, DRAXIN, EBF3, EGLN1, EPHA7, ERBB4, FEZF1, FGFR2, FGFR3, FYN, FZD3, GAP43, GNAO1, HDAC2, HES5, HNRNPK, HOXA1, HOXB1, HOXB2, IPMK, IRX3, KDM6B, KHDRBS1, KIF3A, L1CAM, LEF1, MAP1B, MAPK8, MAPT, MARCKSL1, MGEA5, MSX1, MYCN, NCAM1, NEUROD1, NEUROD4, NEUROG2, NFIA, NFIB, NIN, NOTCH1, NPAS3, NR2F2, NTRK2, OTX1, OTX2, PAX6, PLCB1, POU3F2, POU3F3, PRDM8, PROX1, PTCH1, RAPGEF2, RARB, RERE, RFX4, ROBO2, ROBO3, S1PR1, SIM1, SLC1A2, SLC1A3, SLC4A7, SMAD9, SOX1, SOX2, SOX4, STXBP1, TDG, TOP2B, VEGFA, WLS, YBX1, ZBTB18, ZEB2, ZIC1, ZIC4	99
Organismal Development	morphogenesis	Morphogenesis of neurons	3.3E-23	ACVR2A, APC, ARHGEF7, ASCL1, BASP1, BRSK2, BTBD3, CDH2, CDK5R1, CELSR2, CHL1, CHN1, CKB, CLASP2, CNR1, CNTN1, CNTN2, CNTNAP2, CPEB4, CRMP1, CTNNA2, CUX2, DCC, DCLK1, DCX, DPYSL2, DPYSL3, DPYSL5, ECEL1, EFNB1, EFNB2, ELAVL4, ENC1, EPHA7, ERBB4, FGFR2, FNBP1L, FYN, GABRB3, GAP43, GDI1, GNAO1, GPM6A, GPRIN1, HDAC2, HMGB1, HMGB2, HNRNPK, ID1, KIDINS220, KIF3A, L1CAM, LINGO1, LOX, LYPLA1, MAP1B, MAP2, MAP6, MAPK8, MAPT, NCAM1, NEFM, NEUROD1, NFIA, NFIB, NIN, NOTCH1, NSG1, NTRK2, PAK3, PAX6, PFN2, POU3F2, PPP2CA, PTPRZ1, RAB10, RAB3A, RAPGEF2, RERE, RND2, RNF165, ROBO2, ROBO3, RTN3, RTN4, RUFY3, SKIL, SLC1A3, SLC9A6, SPTBN2, STAU2, STMN1, TOP2B, TPBG, TRIM9, UBQLN2, WASF1, WNT3, ZEB2, ZSWIM6	100
Tissue Development	neuritogenesis	Neuritogenesis	4.68E-23	ACVR2A, APC, ARHGEF7, ASCL1, BASP1, BRSK2, BTBD3, CDH2, CDK5R1, CELSR2, CHL1, CHN1, CKB, CLASP2, CNR1, CNTN1, CNTN2, CNTNAP2, CPEB4, CRMP1, CTNNA2, CUX2, DCC, DCLK1, DCX, DPYSL2, DPYSL3, DPYSL5, ECEL1, EFNB1, EFNB2, ELAVL4, ENC1, EPHA7, ERBB4, FGFR2, FNBP1L, FYN, GABRB3, GAP43, GDI1, GNAO1, GPM6A, GPRIN1, HDAC2, HMGB1, HMGB2,	99

				HNRNPK, ID1, KIDINS220, KIF3A, L1CAM, LINGO1, LOX, LYPLA1, MAP1B, MAP2, MAP6, MAPK8, MAPT, NCAM1, NEFM, NEUROD1, NFIA, NFIB, NIN, NOTCH1, NSG1, NTRK2, PAK3, PAX6, PFN2, POU3F2, PPP2CA, PTPRZ1, RAB10, RAB3A, RAPGEF2, RERE, RND2, RNF165, ROBO2, ROBO3, RTN3, RTN4, RUFY3, SKIL, SLC9A6, SPTBN2, STAU2, STMN1, TOP2B, TPBG, TRIM9, UBQLN2, WASF1, WNT3, ZEB2, ZSWIM6	
Tissue Development	development	Development of cerebral cortex	2.65E-20	ASCL1, ATAT1, BTBD3, CDH2, CDK5R1, CDK6, CHL1, CNR1, CXADR, DCX, DLL1, DLX1, ERBB4, FGFR2, FGFR3, FYN, GAP43, HDAC2, HNRNPK, KDM6B, KIF3A, L1CAM, LEF1, MAPT, MGEA5, NCAM1, NEUROD1, NEUROG2, NFIB, NTRK2, OTX1, OTX2, PAX6, PLCB1, POU3F2, POU3F3, PROX1, SLC1A2, SLC1A3, SOX2, ZBTB18, ZEB2	42
Organismal Development	patterning	Patterning of rostrocaudal axis	6.58E-14	ACVR2A, ACVR2B, APC, HOXA1, HOXA3, HOXA5, HOXB1, HOXB2, HOXB3, HOXB4, HOXB5, HOXB6, HOXB7, HOXB8, HOXB9, HOXD3, KIF3A, MSX1, NEUROD1, NR2F2, OTX1, OTX2, PAX6, TSHZ1, VANGL2, WNT3	26
Tissue Development	axonogenesis	Axonogenesis	6.88E-14	APC, ASCL1, BRSK2, CDH2, CHN1, CLASP2, CNTN2, CTNNA2, DCC, DCLK1, DPYSL2, FGFR2, GAP43, GDI1, L1CAM, MAP1B, MAP2, MAP6, MAPT, NFIA, NFIB, NIN, NOTCH1, NTRK2, PAK3, PAX6, POU3F2, PPP2CA, PTPRZ1, RAB10, RAB3A, RNF165, ROBO2, ROBO3, RTN4, RUFY3, SKIL, SLC9A6, STMN1, TOP2B, TRIM9, ZEB2	42
Tissue Development	formation	Formation of telencephalon	7.26E-14	ARX, ASCL1, BHLHE22, CDH2, CDK5R1, CDK6, CXCR4, DLX1, EBF3, FEZF1, GAP43, GNAO1, HES5, L1CAM, MAP1B, NEUROG2, NFIA, NFIB, NIN, NTRK2, OTX2, PAX6, POU3F2, POU3F3, PRDM8, PTCH1, RARB, RFX4, ROBO2, SLC1A2, TDG, TOP2B, ZEB2	33
Tissue Development	formation	Formation of rhombencephalon	4.88E-13	ASCL1, CCND2, CDK5R1, CNTN1, CXCR4, FGFR3, FYN, HOXA1, HOXB1, KHDRBS1, L1CAM, MGEA5, NEUROD1, NEUROD4, NFIA, NFIB, NPAS3, NTRK2, OTX1, OTX2, PAX6, POU3F2, POU3F3, RERE, RFX4, SLC4A7, SMAD9, SOX4, WLS, ZBTB18, ZIC1, ZIC4	32
Organismal Development	patterning	Patterning of body axis	1.39E-12	ACVR2A, ACVR2B, APC, HOXA1, HOXA3, HOXA5, HOXB1, HOXB2, HOXB3, HOXB4, HOXB5, HOXB6, HOXB7, HOXB8, HOXB9, HOXD3, KIF3A, MSX1, NEUROD1, NR2F2, OTX1, OTX2, PAX6, RFX3, TSHZ1, VANGL2, WNT3	27
Tissue Development	development	Development of sensory organ	1.62E-11	ASCL1, CLCN3, CLIC4, CYP26B1, DLL1, DLX1, EBF1, EBF3, FGFR2, FGFR3, FYN, FZD3, GNAO1, HES5, HES6, HEY1, HOXA1, HS6ST1, ID1, ID3, INSIG1, INSIG2, IRX3, JAG1, KDM6B, KRAS, LEF1, LIN7A, MAP1B, MAP2, MAPK8, MAPT, MARCKSL1, MEIS2, MSX1, MYCN, NCAM1, NES, NEUROD1, NEUROD4, NEUROG1, NEUROG2, NKD1, NOTCH1, NR3C1, NRAS, NTRK2, OTX1, OTX2, PAX6, PIK3R1, PROM1, PROX1, PSIP1, PYGO1, RAD23B, RARB, RBPJ, SLC1A3, SLC4A7, SOX11, SOX2, SOX4, SOX9, STAU2, STC1, TSHZ1, VANGL2, VCAM1, VEGFA, ZEB1	71
Organismal Development	development	Development of sensory organ	1.62E-11	ASCL1, CLCN3, CLIC4, CYP26B1, DLL1, DLX1, EBF1, EBF3, FGFR2, FGFR3, FYN, FZD3, GNAO1, HES5, HES6, HEY1, HOXA1, HS6ST1, ID1, ID3, INSIG1, INSIG2, IRX3, JAG1, KDM6B, KRAS, LEF1, LIN7A, MAP1B, MAP2, MAPK8, MAPT, MARCKSL1, MEIS2, MSX1, MYCN, NCAM1, NES, NEUROD1, NEUROD4, NEUROG1, NEUROG2, NKD1, NOTCH1, NR3C1, NRAS, NTRK2, OTX1, OTX2, PAX6, PIK3R1, PROM1, PROX1, PSIP1, PYGO1, RAD23B, RARB, RBPJ, SLC1A3, SLC4A7, SOX11, SOX2, SOX4, SOX9, STAU2, STC1, TSHZ1, VANGL2, VCAM1, VEGFA, ZEB1	71
Tissue Development	development	Development of metencephalon	6.33E-11	CCND2, CDK5R1, CNTN1, CXCR4, FGFR3, FYN, KHDRBS1, L1CAM, MGEA5, NEUROD1, NEUROD4, NFIA, NPAS3, NTRK2, OTX1, OTX2, PAX6, POU3F2, POU3F3, RERE, RFX4, SLC4A7, SOX4, ZBTB18, ZIC1, ZIC4	26
Tissue Development	formation	Formation of hippocampus	2.33E-10	ATAT1, CDK5R1, CDK6, CNR1, CXADR, DCX, DLX1, FGFR3, FYN, HDAC2, KDM6B, KIF3A, L1CAM, LEF1, MAPT, NCAM1, NEUROD1, NFIB, OTX1, OTX2, PROX1, ZBTB18, ZEB2	23
Organismal Development	formation	Formation of hippocampus	2.33E-10	ATAT1, CDK5R1, CDK6, CNR1, CXADR, DCX, DLX1, FGFR3, FYN, HDAC2, KDM6B, KIF3A, L1CAM, LEF1, MAPT, NCAM1, NEUROD1, NFIB, OTX1, OTX2, PROX1, ZBTB18, ZEB2	23
Organ Development	formation	Formation of hippocampus	2.33E-10	ATAT1, CDK5R1, CDK6, CNR1, CXADR, DCX, DLX1, FGFR3, FYN, HDAC2, KDM6B, KIF3A, L1CAM, LEF1, MAPT, NCAM1, NEUROD1, NFIB, OTX1, OTX2, PROX1, ZBTB18, ZEB2	23

Tissue Development	branching	Dendritic growth/branching	4.3E-10	ARHGEF7, CDH2, CHN1, CLASP2, CNR1, CNTNAP2, CPEB4, CRMP1, CTNNA2, DCX, DPYSL5, EFNB1, ELAVL4, ERBB4, GABRB3, GAP43, GNAO1, HNRNPK, KIDINS220, KIF3A, L1CAM, LYPLA1, MAPT, NEFM, NEUROD1, NOTCH1, NSG1, NTRK2, PFN2, RAPGEF2, RND2, ROBO2, RTN4, SLC9A6, SPTBN2, STAU2, TPBG, UBQLN2	38
Tissue Development	differentiation	Differentiation of retinal cells	5.91E-10	ASCL1, DLX1, EBF1, EBF3, HES6, KDM6B, NEUROD1, NEUROD4, NKD1, NOTCH1, OTX1, OTX2, SOX9	13
Tissue Development	proliferation	Proliferation of epithelial cells	6.93E-10	ACVR2A, APC, ATF2, BIRC2, CCND2, CCNG2, CDC25B, CDK6, CDKN1C, CITED2, CNR1, CXCR4, DCC, EDNRB, EFNB2, ERBB4, FGFR2, FGFR3, FYN, FZD7, GPC3, HES5, HEY1, HOXA5, HOXB7, ID1, ITGB8, KCNK2, KDM5B, KIF3A, KLF5, KRAS, MAPK8, NEUROD1, NFE2L2, NFIB, NOTCH1, NR3C1, NRAS, PAK2, PAQR3, PAX6, POU3F2, PROX1, PTCH1, PTPRZ1, RBPJ, RGS4, SOX11, SOX2, SOX4, SOX9, STMN1, TCF7L2, TFRC, TOB1, TRIM24, USP11, USP7, VEGFA, ZFP36L1	61
Tissue Development	formation	Formation of cerebellum	1.21E-09	CCND2, CDK5R1, CNTN1, CXCR4, FGFR3, FYN, KHDRBS1, L1CAM, NEUROD1, NEUROD4, NFIA, NPAS3, NTRK2, OTX2, PAX6, POU3F2, POU3F3, RERE, RFX4, SLC4A7, SOX4, ZBTB18, ZIC1, ZIC4	24
Organismal Development	formation	Formation of cerebellum	1.21E-09	CCND2, CDK5R1, CNTN1, CXCR4, FGFR3, FYN, KHDRBS1, L1CAM, NEUROD1, NEUROD4, NFIA, NPAS3, NTRK2, OTX2, PAX6, POU3F2, POU3F3, RERE, RFX4, SLC4A7, SOX4, ZBTB18, ZIC1, ZIC4	24
Organ Development	formation	Formation of cerebellum	1.21E-09	CCND2, CDK5R1, CNTN1, CXCR4, FGFR3, FYN, KHDRBS1, L1CAM, NEUROD1, NEUROD4, NFIA, NPAS3, NTRK2, OTX2, PAX6, POU3F2, POU3F3, RERE, RFX4, SLC4A7, SOX4, ZBTB18, ZIC1, ZIC4	24
Tissue Development	development	Development of neuroglia	2.86E-09	ASCL1, CDK6, DLL1, DLX1, ERBB4, FGFR2, FGFR3, HES5, HEY1, KRAS, NEUROD4, NEUROG2, NOTCH1, POU3F2, RBPJ, SOX11, SOX4, TCF7L2, WASF3	19
Organismal Development	formation	Formation of dendrites	1.33E-08	BTBD3, CDH2, CELSR2, CKB, CTNNA2, CUX2, DCLK1, DCX, EFNB1, ELAVL4, FYN, HDAC2, ID1, KIDINS220, L1CAM, LOX, MAP1B, MAP2, MAP6, MAPK8, NFIA, NTRK2, PAK3, PTPRZ1, RAPGEF2, RERE	26
Organismal Development	formation	Formation of eye	4.97E-08	ASCL1, CLCN3, CLIC4, DLX1, EBF1, EBF3, FGFR2, FYN, HES5, HES6, HS6ST1, ID1, ID3, IRX3, JAG1, KDM6B, KRAS, LEF1, MAP1B, MAPK8, MAPT, MARCKSL1, MEIS2, NCAM1, NEUROD1, NEUROD4, NEUROG2, NKD1, NOTCH1, NR3C1, NRAS, NTRK2, OTX1, OTX2, PAX6, PIK3R1, PROM1, PSIP1, PYGO1, RAD23B, RARB, RBPJ, SLC1A3, SLC4A7, SOX11, SOX4, SOX9, STAU2, STC1, VCAM1, VEGFA, ZEB1	52
Organ Development	proliferation	Proliferation of brain cells	7.07E-08	ARX, CNR1, CUX2, GAP43, GNAS, HLTF, ID1, ID3, KIF3A, MAPT, NEUROD1, NPAS3, PAX6, RTN4, SOX11, SOX2, VEGFA, ZBTB18, ZIC1, ZNF503	20
Tissue Development	maturation	Maturation of neurons	7.65E-08	ASCL1, CDKN1C, CNR1, DCLK1, EFNB1, EPHA7, ERBB4, HDAC2, MAPT, NEUROD1, NFIA, NRXN1, RAB3A, SEZ6L, SLC12A2, STXBP1	16
Organismal Development	development	Development of spinal cord	0.000000111	ASCL1, DLL1, DRAXIN, HOXB8, NTRK2, PAX6, PBX3, PROX1, RFX4, SOX11, SOX4, TOP2B, ZIC1	13
Organ Development	development	Development of spinal cord	0.000000111	ASCL1, DLL1, DRAXIN, HOXB8, NTRK2, PAX6, PBX3, PROX1, RFX4, SOX11, SOX4, TOP2B, ZIC1	13
Tissue Development	differentiation	Differentiation of photoreceptors	0.000000165	ASCL1, DLX1, EBF1, EBF3, HES6, KDM6B, NKD1, NOTCH1, SOX9	9
Tissue Development	proliferation	Proliferation of neural precursor cells	0.000000242	ARX, CNR1, CUX2, GAP43, HLTF, ID1, ID3, KIF3A, NPAS3, PAX6, SOX11, SOX2, VEGFA, ZBTB18, ZNF503	15
Organ Development	development	Development of ear	0.000000506	DLL1, FGFR2, FGFR3, FZD3, HES5, HEY1, HOXA1, INSIG1, INSIG2, JAG1, LIN7A, MSX1, MYCN, NEUROD1, NEUROG1, NOTCH1, OTX1, OTX2, PROX1, SOX2, SOX9, TSHZ1, VANGL2, VEGFA, ZEB1	25
Tissue Development	morphogenesis	Morphogenesis of forebrain	0.00000054	BHLHE22, CDH2, IRX3, NFIB, NIN, OTX1, OTX2, PRDM8, SOX2, ZEB2	10
Organ Development	foliation	Foliation of cerebellum	0.000000765	CCND2, CDK5R1, KHDRBS1, L1CAM, NFIA, NPAS3, POU3F2, POU3F3, ZBTB18, ZIC1, ZIC4	11
Tissue Development	formation	Formation of brain cells	0.00000202	ARX, ASCL1, CHL1, CNR1, CXCR4, FGFR2, KIF3A, L1CAM, NCAM1, NEUROD1, NEUROG2, NOTCH1, POU3F2	13
Organ Development	development	Development of lacrimal gland	0.00000206	FGFR2, HS6ST1, OTX1, OTX2, PAX6, SOX9	6
Tissue Development	development	Development of astrocytes	0.00000238	ASCL1, CDK6, DLL1, ERBB4, HEY1, KRAS, NEUROG2, POU3F2, RBPJ	9

Table S15. Gene counts for POU4F2+ organoids (corresponds to Fig. 7)

GENEID	NEG1	NEG2	NEG3	NEG4	SIX6_1	SIX6_2	SIX6_3	SIX6_4	B3B_1	B3B_2	B3B_3	B3B_4
ALDH1A3	1.00282224	3.8289743	4.86875659	1.36221165	1706.60327	2746.44637	24.3672074	12.6997693	5336.70244	6580.60827	1011.5035	1171.14382
ATOH7	10.0282224	15.3158972	12.1718915	21.7953864	55.4670825	0	30.9276093	9.52482696	4485.57376	2708.89057	2241.76099	4306.97726
CACNG5	29.0818448	16.8474869	13.3890806	17.7087515	1.98096723	0	0	0	166.168143	57.1574974	44.8726136	82.599564
CCDC152	39.1100672	64.3267682	109.547023	55.8506777	455.622464	561.507681	331.7689	329.135687	691.723198	505.984403	436.573137	576.230291
CDON	1767.9756	1613.52977	1816.04621	1814.46592	1568.92605	1745.61508	1387.05642	1529.26388	7298.83905	6604.03347	5727.80216	12709.5162
CHRNA3	62.1749786	38.289743	37.7328635	44.9529845	61.4099842	129.37137	58.1064175	93.1316414	1140.95684	640.913578	386.091446	526.080556
CGN	44.1241784	52.0740504	47.4703767	47.6774078	338.745397	336.904608	391.749718	311.144347	1078.16074	1049.44913	979.718731	1185.89374
CLSTN2	177.499536	147.032613	198.401831	134.858953	498.213259	517.485478	530.45536	263.520213	692.689292	784.275825	597.366669	842.712218
CP	49.1382896	60.4977939	43.8188093	42.2285612	122.819968	158.120563	89.9712272	116.414552	1927.35724	713.063205	331.870372	766.012623
CNTN2	2579.25879	2589.15242	1220.84071	2017.43545	541.794538	793.298051	572.629373	632.871836	11259.8239	5010.1826	4034.79584	8385.8224
COL25A1	84.2370678	80.4084602	62.0766465	96.7170272	1571.8975	1478.78663	1562.31287	1222.35279	2955.28109	2447.4653	3683.2937	3949.04582
DCT	48.1354673	44.4161018	51.1219441	27.244233	1277.72387	7781.14883	1193.05596	2049.95443	3896.25651	11246.9089	3129.8648	4325.6605
DIO2	1.00282224	6.12635887	10.9547023	9.53548155	419.965053	238.079257	388.938117	233.887418	277.268936	269.858348	708.613357	132.749299
DIO3	129.364068	170.772254	169.189291	121.236837	1062.78892	715.136182	537.015762	613.822182	1765.05347	1354.91379	903.996195	271.398567
DAPL	1.00282224	1.53158972	1.21718915	0	88.1530418	61.0920356	58.1064175	23.2829104	2599.75856	2744.49688	1940.74054	3368.88222
EYA2	133.375357	127.887742	81.5516728	141.670012	260.497191	418.660127	587.624577	422.267329	441.50489	794.582915	936.715809	1125.91072
EGFR	52.1467563	76.5794859	38.9500527	68.1105825	329.831044	187.768168	115.275635	139.697462	1178.6345	908.89791	620.737822	1507.44204
FEZF2	44.1241784	212.890971	14.6062698	72.1972175	447.698595	607.326707	341.140903	295.269636	950.63635	1089.74048	769.378354	1539.89187
FAM111A	120.338668	138.60887	133.890806	175.725303	443.73666	696.269524	363.63371	319.61086	908.128221	755.228572	693.655819	839.762234
FHIP1A	23.0649114	50.5424607	37.7328635	28.6064447	120.839001	265.031625	158.386848	182.030026	495.606146	446.95289	615.128745	986.278127
FOS (AP-1)	189.533403	221.314714	292.125395	204.331748	983.550231	986.456693	739.451023	826.543317	4659.47065	1315.55945	1753.77132	628.346683
GRIA4	432.216384	490.10871	548.952305	313.30868	116.877067	57.4983865	90.9084274	109.006353	970.924321	857.362461	521.644133	1609.70817
HAS2	59.1665119	97.2559471	85.2032402	42.2285612	255.544773	308.155415	226.802469	122.764436	802.823991	1227.48068	1681.78816	2064.9891
LEFTY2	3.00846671	0	3.65156744	1.36221165	636.880965	300.069705	466.725741	324.902431	538.114276	1018.52786	1311.5891	298.931755
LHX2	10.0282224	16.8474869	7.30313488	35.4175029	925.111698	811.266297	427.363329	541.856823	1434.64937	1651.00837	1787.42578	1969.60627
LHX4	8.02257789	8.42374345	3.65156744	5.4488466	3.96193447	0	3.74880113	3.17494232	28.9828156	17.8031549	12.1529995	22.6165473
MGARP	36.1016005	53.6056401	47.4703767	47.6774078	56.4575661	97.0285272	51.5460156	76.1986157	1186.36325	829.252217	575.865208	1069.86102
ME3	94.2652902	108.74287	102.243888	89.9059689	246.63042	442.917258	162.135649	149.222289	476.284269	525.661575	445.921598	622.446714
OPTC	0	0	0	0	2.97145085	0	0.93720028	0	59.8978188	73.086636	15.892384	54.0830478
PAX2	5.01411118	0	7.30313488	2.7244233	78.2482057	101.520589	1.87440057	7.40819875	134.287045	46.8504077	8.41361505	1.96665629
PAX6	1927.42434	1031.52568	590.336736	4180.62756	285.259282	935.247193	89.9712272	113.239609	8914.14797	6137.40341	5244.48672	9172.48491
POU4F2	4.01128894	1.53158972	1.21718915	2.7244233	9.90483616	15.2730089	0.93720028	0	1310.98936	425.401702	395.439907	818.129014
RAX	4.01128894	10.721128	4.86875659	13.6221165	1243.05694	720.526656	956.881489	781.035811	3792.88446	3464.11915	3430.88525	3867.42958
RBP3	0	0	0	0	2.97145085	0	1.87440057	0	294.658625	238.000071	202.861607	708.979591
SEMA3A	191.539047	167.709074	131.456428	174.363091	599.242588	455.49503	425.488928	501.640887	1510.97079	1470.16579	1402.26918	2243.95482
SEMA3F	586.651008	457.179531	271.43318	467.238596	392.231512	348.583968	328.020099	414.85913	1319.6842	944.50422	1195.66818	1382.55937
SIX3	9.02540012	4.59476916	6.08594573	31.330868	717.110138	296.476055	605.431383	673.087772	494.640052	438.519816	695.525511	520.180587
SIX6	0	0	0	0	1548.12589	574.983865	892.214669	944.016183	1911.89973	1288.38621	817.055506	1095.42755
SHISA2	35.0987783	36.7581532	38.9500527	32.6930796	783.47254	838.218665	456.416538	348.185341	950.63635	1519.82723	1118.07596	1761.1407
SFRP1	539.518363	1019.27296	335.944204	709.71227	2022.56754	2343.95766	1636.35169	1739.86839	3570.68288	3247.67026	3201.84795	3512.44812

**SFRP2**	576.622786	546.777529	433.319336	968.532484	680.462244	864.272622	360.822109	446.608553	12084.868	9688.66432	3955.33392	7451.66066
**SFRP5**	2.00564447	19.9106663	6.08594573	8.1732699	24.7620904	7.18729831	98.4060297	146.047347	26.084534	383.236335	300.085604	370.71471
**S1PR3**	36.1016005	41.3529224	64.5110248	59.9373126	711.167236	389.910933	106.840832	209.546193	3573.58116	1738.15013	636.630206	796.495795
**STRA6**	112.31609	99.5533317	48.6875659	76.2838524	945.911854	1206.5677	870.659063	775.74424	2283.84587	1820.60684	1802.38331	1619.54145
**TBX2**	6.01693341	16.081692	13.3890806	2.7244233	192.153822	82.6539306	164.01005	125.939379	2505.08136	788.023858	808.641891	835.828921
**TBX3**	16.0451558	49.010871	38.9500527	24.5198097	2152.3209	1772.56745	1769.43413	1667.90303	545.843026	2073.59905	4070.31999	3464.26505
**TFAP2D**	0	0	0	0	0.99048362	0	0	0	86.9484467	29.0472528	22.4363068	36.3831413
**THRB**	100.282224	113.337639	92.5063751	126.685684	33.676443	37.7333161	40.2996122	32.8077373	979.619166	480.685183	378.612677	776.829232
**TLE1**	697.964276	592.725221	474.703767	817.32699	558.63276	399.793469	459.228139	432.85047	1217.27825	1067.25229	937.650655	1126.89405
**VAX1**	2.00564447	2.29738458	3.65156744	4.08663495	782.482057	729.510779	333.643301	512.224028	319.777065	299.842609	93.4846117	1.96665629
**VAX2**	44.1241784	47.4792813	38.9500527	49.0396194	38.628861	6.28888602	16.8696051	21.1662821	548.741308	542.527721	277.649297	503.464009
**VSX1**	3.00846671	1.53158972	4.86875659	1.36221165	3.96193447	0	0.93720028	3.17494232	200.947521	126.496101	93.4846117	125.866002
**VSX2**	2.00564447	0.76579486	4.86875659	2.7244233	101.029329	53.006325	23.4300071	8.46651286	5055.56913	4365.52099	3437.42917	6826.26396

Table S16. Gene counts for SIX6+ organoids (corresponds to Fig. 7)

**GENEID**	**NEG1**	**NEG2**	**NEG3**	**NEG4**	**SIX6_1**	**SIX6_2**	**SIX6_3**	**SIX6_4**	**B3B_1**	**B3B_2**	**B3B_3**	**B3B_4**
**CYSLYR2**	0	6.89215373	0	2.7244233	272.382995	159.018975	252.106876	239.178988	114.965168	197.708721	349.632448	245.832036
**FEZF2**	44.1241784	212.890971	14.6062698	72.1972175	447.698595	607.326707	341.140903	295.269636	950.63635	1089.74048	769.378354	1539.89187
**FOXG1**	1.00282224	0.76579486	1.21718915	1.36221165	329.831044	396.199819	683.219006	149.222289	52.169068	74.0236442	13.0878456	14.7499221
**FZD5**	41.1157117	56.6688196	59.6422682	36.7797146	7537.58032	5826.20369	6886.54768	5838.71893	11565.1095	8253.16782	9111.9451	12504.0007
**GHRH**	5.01411118	3.8289743	2.43437829	1.36221165	65.3719187	48.5142636	42.1740127	53.9740195	4.83046926	4.68504077	5.6090767	1.96665629
**GPR50**	21.059267	45.1818967	37.7328635	25.8820214	2529.69516	2812.92888	2155.56065	2307.12475	126.558295	490.992273	1451.81602	121.93269
**GPR158**	37.1044227	56.6688196	73.0313488	49.0396194	272.382995	132.965019	325.208498	278.33661	126.558295	67.4645871	61.6998437	32.4498287
**HAP1**	73.2060232	74.2821013	82.7688619	64.0239476	450.670045	238.079257	263.35328	380.993079	189.354395	135.866182	143.031456	59.9830167
**HDC**	6.01693341	7.65794859	1.21718915	8.1732699	67.3528859	94.3332903	74.0388224	70.9070452	5.79656311	16.8661468	14.9575379	0
**ICAM1**	11.0310446	16.8474869	19.4750263	1.36221165	118.858034	288.390345	134.956841	188.379911	126.558295	123.685076	107.507303	87.5162047
**N4BP2L1**	30.0846671	21.4422561	36.5156744	43.5907728	120.839001	245.266555	172.444852	209.546193	126.558295	136.803191	259.887221	294.015115
**NKX2-1**	204.575736	429.610916	159.451778	442.718786	2032.47238	999.034465	2522.94316	2113.45327	131.388764	407.598547	876.885658	201.582269
**NKX2-4**	96.2709346	152.393177	103.461077	204.331748	160.458346	186.869756	179.005254	219.07102	0	14.9921305	68.2437665	13.766594
**OLIG1**	83.2342456	101.850716	17.0406481	88.5437573	205.030109	39.5301407	36.550811	66.6737887	2.89828156	13.1181142	14.0226918	2.94998443
**OLIG2**	230.649114	202.935638	85.2032402	227.489346	357.564586	142.847554	111.526834	168.271943	79.2196959	99.3228644	108.44215	145.532565
**OTP**	0	9.18953831	8.52032402	4.08663495	41.6003119	30.5460178	40.2996122	37.0409937	0.96609385	3.74803262	1.86969223	0
**POMC**	5.01411118	3.06317944	1.21718915	2.7244233	415.012635	187.768168	183.691255	122.764436	110.134699	256.740234	136.487533	139.632596
**POU3F1**	184.519291	115.635024	177.709615	149.843282	484.346488	104.215826	500.464951	473.066406	171.964706	103.070897	198.187377	265.498598
**PTPRB**	16.0451558	22.2080509	20.6922155	8.1732699	1608.54539	1437.45966	2302.7011	1881.68248	224.133774	440.393833	616.063591	117.016049
**RAB37**	32.0903115	24.5054355	20.6922155	24.5198097	136.686739	31.4444301	106.840832	101.598154	15.4575016	44.0393832	41.1332291	15.7332503
**SHH**	828.331167	938.864497	816.733917	498.569464	1492.65881	625.294953	2196.79746	2329.34935	12.5592201	10.3070897	86.9406889	5.89996885
**SLCO1C1**	1.00282224	4.59476916	2.43437829	1.36221165	458.593914	917.278947	549.199366	376.759822	116.897356	175.220525	99.0936884	13.766594
**SPRY4**	28.0790226	14.5501023	36.5156744	5.4488466	349.640717	202.142765	200.560861	250.820443	375.810508	234.252039	259.887221	239.932067
**SOCS3**	58.1636897	46.7134864	54.7735116	72.1972175	500.194226	624.396541	447.044535	341.835457	658.876007	782.401809	783.401046	491.664071
**SOX2**	4018.3087	4501.34218	5429.88078	5094.67157	6905.65177	4794.82639	6030.88382	6507.57344	3876.93463	3161.46551	3576.72124	3480.98162
**SOX6**	160.451558	180.727587	166.754913	250.646944	713.148204	919.075771	941.886284	842.418029	112.066887	170.535484	198.187377	76.6995951

**SOX21**	106.299157	89.5979985	180.143994	174.363091	320.916692	159.917387	392.686919	263.520213	19.321877	49.6614322	46.7423058	4.91664071
**SST**	97.2737569	54.371435	18.2578372	81.732699	74.2862712	50.3110882	65.6040198	61.3822182	59.8978188	28.1102446	28.0453835	5.89996885

Table S17. Gene counts for SIX6 – organoids (NEG; corresponds to Fig. 7)

GENEID	NEG1	NEG2	NEG3	NEG4	SIX6_1	SIX6_2	SIX6_3	SIX6_4	B3B_1	B3B_2	B3B_3	B3B_4
**ARX**	211.595492	130.950921	136.325184	264.26906	92.1149763	10.7809475	29.0532088	40.2159361	5.79656311	6.55905708	18.6969223	4.91664071
**ABCA8**	100.282224	236.630612	233.700316	156.65434	10.8953198	9.88253518	13.120804	27.5161668	3.86437541	12.181106	6.54392282	4.91664071
**BARHL1**	468.317984	267.262406	383.414581	736.956503	2.97145085	25.1555441	0.93720028	5.29157053	0	1.87401631	1.86969223	0.98332814
**CRH**	22.0620892	88.0664088	57.2078899	54.488466	2.97145085	0	4.68600141	7.40819875	0	3.74803262	3.73938447	0
**DBX1**	121.341491	136.311485	25.5609721	108.976932	0	0	6.56040198	10.5831411	15.4575016	6.55905708	14.0226918	22.6165473
**EBF3**	832.342456	598.85158	842.294889	1288.65222	28.7240249	208.431651	19.6812059	22.2245962	821.179774	242.685112	121.529995	87.5162047
**EMX2**	512.442162	537.587991	288.473828	438.632152	6.93338531	32.3428424	39.3624119	110.064667	3.86437541	41.2283588	101.898227	82.599564
**FERD3L**	50.1411118	47.4792813	82.7688619	39.5041379	1.98096723	0	1.87440057	0	0	0	0	0
**FOXA1**	957.695235	763.497475	1225.70947	1057.07624	54.4765989	35.9364916	54.3576164	100.53984	20.2879709	29.0472528	17.7620762	15.7332503
**FOXA2**	85.23989	85.7690242	206.922155	85.819334	11.8858034	0	7.49760226	35.9826796	0.96609385	0	2.80453835	0
**FOXB1**	61.1721564	94.1927677	73.0313488	57.2128893	2.97145085	0	4.68600141	4.23325643	0.96609385	2.81102446	0	0
**FOXD1**	317.894649	271.857175	233.700316	324.206373	97.0673944	203.041177	72.1644218	215.896078	56.0334434	125.559093	69.1786127	109.149424
**FOXJ1**	493.38854	520.740504	692.580624	696.090153	104.991263	53.006325	107.778033	135.464206	58.931725	97.448848	95.3543039	114.066065
**GABRG2**	270.762004	290.236252	304.297287	280.6156	131.734321	72.7713954	142.454443	118.53118	24.1523463	23.4252039	31.784768	13.766594
**GRM8**	193.544692	191.448715	268.998801	284.702235	113.905616	137.45708	136.831241	166.155315	17.3896893	14.9921305	9.34846117	5.89996885
**HOXA3**	223.629359	131.716716	166.754913	117.150202	20.8001559	2.69523687	11.2464034	19.0496539	4.83046926	13.1181142	28.9802296	0.98332814
**HOXB2**	675.902187	493.937684	707.186894	569.40447	89.1435255	44.0222022	53.4204161	82.5485003	30.9150033	23.4252039	78.5270738	10.8166096
**HOXB9**	1273.58424	1088.1945	1209.88601	625.255148	52.4956317	174.291984	37.4880113	38.0993079	17.3896893	35.6063099	65.4392282	9.83328142
**KCNQ3**	1397.9342	1350.86213	1252.48763	1206.91952	316.954757	430.339486	345.826904	400.042732	280.167217	133.992166	161.728378	123.899346
**LMX1A**	1803.07438	1389.15187	1662.68037	2614.08416	9.90483616	36.8349038	17.8068054	9.52482696	7.72875082	32.7952854	13.0878456	2.94998443
**LMX1B**	679.913476	624.888605	702.318137	1304.99876	5.9429017	0	2.81160085	8.46651286	0	2.81102446	3.73938447	0.98332814
**LOC400499**	372.047049	353.797225	555.038251	237.024827	83.2006238	61.9904479	80.5992243	102.656468	9.66093852	12.181106	16.8272301	7.86662514
**LRAT**	184.519291	192.980305	73.0313488	223.402711	42.5907955	50.3110882	58.1064175	91.0150132	17.3896893	18.7401631	19.6317685	7.86662514
**MASP1**	1372.86364	1394.51244	1598.16935	1381.28261	249.601871	157.222151	373.005713	371.468252	24.1523463	17.8031549	69.1786127	19.6665629
**NEUROG2**	1120.15244	945.756651	826.47143	1349.95175	10.8953198	146.441203	2.81160085	21.1662821	261.811434	171.472492	151.445071	313.681677
**NHLH2**	678.910654	498.532453	662.150896	482.222924	48.5336972	198.549116	28.1160085	32.8077373	498.504428	177.094541	85.0709966	35.3998131
**NKX2-2**	215.606781	159.285331	276.301936	136.221165	63.3909514	3.59364916	28.1160085	104.773097	0	2.81102446	3.73938447	1.96665629
**NRN1**	6948.55527	4546.52408	4604.62654	5756.70644	888.463804	895.717052	841.605854	888.98385	994.110574	609.992308	953.543039	604.746808
**OLIG3**	1367.84953	1389.91767	1222.0579	1058.43845	3.96193447	0	4.68600141	16.9330257	0	2.81102446	1.86969223	0
**PID1**	209.589847	130.185126	216.659668	238.387039	104.991263	27.850781	99.34323	96.3065837	14.4914078	15.9291386	43.9377675	0.98332814
**PITX2**	1723.85142	1665.60382	1147.80937	2314.39759	2.97145085	0	8.43480255	28.5744809	4.83046926	6.55905708	4.67423058	1.96665629
**PITX3**	47.1326451	28.3344098	30.4297287	21.7953864	0	10.7809475	1.87440057	0	4.83046926	1.87401631	0.93484612	0
**POU3F2**	1838.17316	1886.91853	1929.2448	1924.80506	54.4765989	324.326836	67.4784204	128.056007	99.5076668	73.086636	86.0058428	74.7329388
**POU3F3**	424.193806	433.43989	449.142795	371.883781	34.6669266	56.5999742	15.9324048	42.3325643	0	6.55905708	10.2833073	0
**POU3F4**	218.615247	247.35174	166.754913	185.260785	1.98096723	0	14.9952045	16.9330257	0	0.93700815	2.80453835	0

**PCSK1**	2563.21363	1660.24326	1788.05086	1404.44021	175.3156	37.7333161	235.237271	159.80543	102.405948	91.8267991	42.0680753	29.4998443
**RSPO2**	1152.24275	1334.01465	1222.0579	2220.40499	8.91435255	17.9682458	16.8696051	24.3412245	0.96609385	14.9921305	26.1756913	12.7832659
**SETBP1**	2209.21739	2147.28879	1879.34004	2198.6096	605.18549	619.006067	600.745381	641.338349	528.453337	339.196952	347.762756	396.281241
**SIM1**	2679.54101	1791.95997	1609.12405	765.562948	11.8858034	70.9745708	21.5556065	26.4578527	8.69484467	11.2440979	15.892384	8.84995328
**SP5**	265.747893	241.225381	182.578372	298.324352	19.8096723	0	53.4204161	80.4318721	7.72875082	35.6063099	28.9802296	23.5998754
**UNCX**	210.59267	120.995588	163.103346	143.032223	0	8.98412289	0	2.11662821	1.9321877	0	0.93484612	0
**VAV3**	849.390434	745.118398	833.774565	1061.16288	11.8858034	0	10.3092031	20.107968	36.7115664	76.8346687	71.983151	59.9830167
